# The influence of intraannular templates on the liquid crystallinity of shape-persistent macrocycles

**DOI:** 10.3762/bjoc.10.89

**Published:** 2014-04-23

**Authors:** Joscha Vollmeyer, Ute Baumeister, Sigurd Höger

**Affiliations:** 1 Kekulé-Institut für Organische Chemie und Biochemie, Rheinische Friedrich-Wilhelms-Universität Bonn, Gerhard-Domagk-Str. 1, 53121 Bonn, Germany; 2 Institut für Chemie, Physikalische Chemie, Martin-Luther-Universität Halle-Wittenberg, Von-Danckelmann-Platz 4, 06120 Halle (Saale), Germany

**Keywords:** discotic liquid crystals, shape-persistent macrocycles, templates, X-ray scattering

## Abstract

A series of shape-persistent phenylene–ethynylene–naphthylene–butadiynylene macrocycles with different extraannular alkyl groups and intraannular bridges is synthesized by oxidative Glaser-coupling of the appropriate precursors. The intraannular bridges serve in this case as templates that reduce the oligomerization even when the reaction is not performed under pseudo high-dilution conditions. The extraannular as well as the intraannular substituents have a strong influence on the thermal behavior of the compounds. With branched alkyl chains at the periphery, the macrocycles exhibit liquid crystalline (lc) phases when the interior is empty or when the length of the alkyl bridge is just right to cross the ring. With a longer alkyl or an oligoethylene oxide bridge no lc phase is observed, most probably because the mesogene is no longer planar.

## Introduction

The supramolecular chemistry of shape-persistent macrocycles has enormously expanded during the past several years [1–6]. It covers the non-covalent interaction between the compound molecules and also the interaction between the macrocycles and appropriate partners. For example, the 2D organization of shape-persistent macrocycles at suitable surfaces leads to long-range ordered patterns with nanoscale lattice parameters and, moreover, even to the epitaxial absorption of appropriate guest molecules on this macrocycle template [7–10]. In solution, shape-persistent macrocycles aggregate into defined dimers or up to μm long fibers that can form gels (in solution) or can be casted to yield efficient sensor materials [11–23]. Amphiphilic macrocycles in aqueous solution have been shown to be able to form vesicles [24–25]. In the bulk state, most of the macrocycles crystallize and some could be explored by single-crystal X-ray analysis [26–28]. Heating crystalline macrocycles above the melting point does not always lead immediately to an isotropic melt, but thermotropic mesophases are observed when the macrocycles have an appropriately substituted rim [29–35]. If the macrocycles pack on top of each other, hexagonal columnar or rectangular columnar phases can be observed in which the (empty) interior is able to accommodate small guest molecules [36–39]. In some cases, however, macrocycles with a filled interior seem to exhibit more stable mesophases compared to the compounds alike but with an empty interior [40]. Moreover, it has also been observed that even macrocycles with a flexible interior only, lacking the flexible rim, can form stable mesophases (macrocycles with an inverse structure) [41–43].

**Figure 1:**
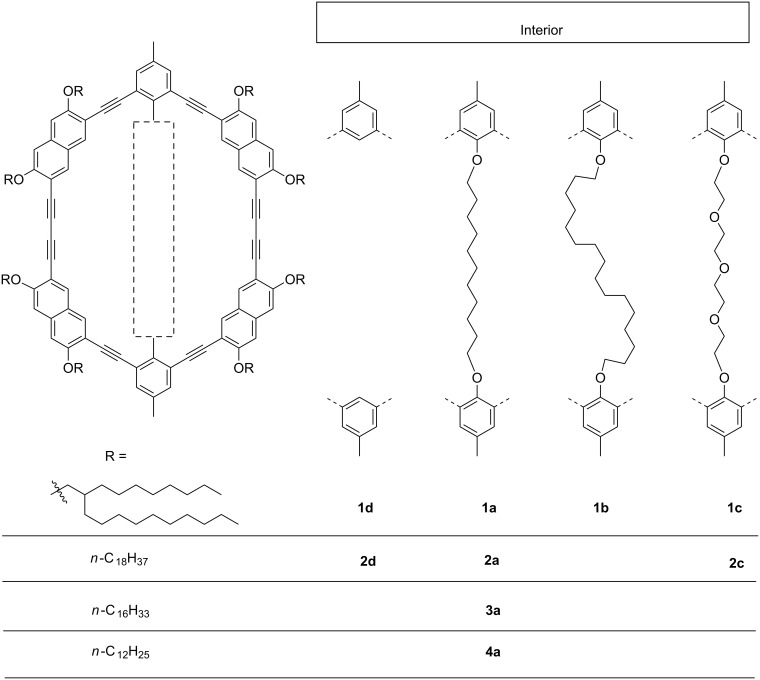
Shape-persistent macrocycles with different peripheral side groups and intraannular templates.

Recently, we presented a series of gel forming macrocycles that have an identical periphery but bear different intraannular substituents [11]. We were able to show that these substituents influence the thermal stability of the gel. As pointed out before, the ring interior can also have a dramatic effect on the mesophase stability of thermotropic liquid crystalline shape-persistent macrocycles [40,43–45]. However, more detailed studies on that issue are still scarcely found in the literature. Here, we designed and synthesized macrocycles **1**–**4** with flexible extraannular alkyl groups and a fixed intraannular chain that crosses the ring interior (Figure 1) and acts during the synthesis as a template. We studied the influence of the extra- and intraannular substituents on the molecule’s ability to form liquid crystalline phases. The macrocycles are based on a phenylene–ethynylene–naphthylene–butadiynylene backbone. Naphthylene units at the four corners are expected to have a higher mesomorphic tendency compared with compounds solely based on phenylene units [46]. Moreover, the naphthylene corners allow an efficient surrounding of the macrocycles with an alkyl fringe, according to the general design principle for discotic liquid crystals [47]. In addition to the macrocycles with intraannular bridges, we also synthesized and investigated a corresponding compound with an empty interior (**1d**).

## Results and Discussion

### Synthesis

#### Template-based macrocycles

The synthesis of the macrocycle **1** follows our often used strategy to dimerize appropriate rigid bisacetylenes oxidatively [48]. This coupling reaction can be performed statistically or template supported, where the latter is either non-covalently or covalently bound to the bisacetylenes [45,49–50]. The template does not necessarily only support the desired cyclization, it can also take over an active function in the final target structure. Covalently attached templates have the advantage over most of the supramolecular templates of being robust against solvent or temperature changes and will still be applicable at elevated temperatures. The bisacetylenes can be prepared independently and attached to the template just prior to the cyclization reaction or, and this is done here, the (template bound) oligoacetylene is prepared at the template [51].

**Scheme 1:**
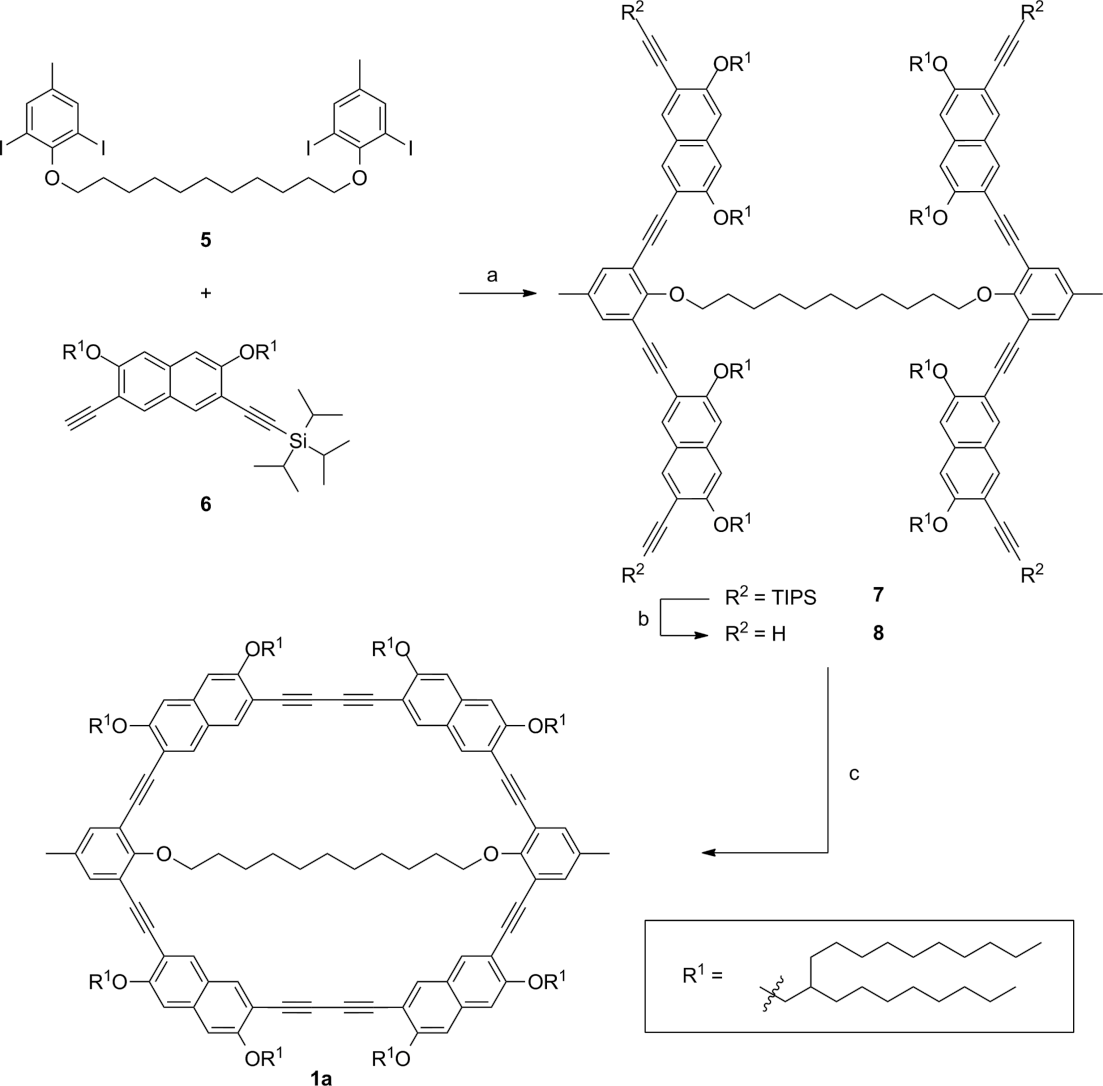
Synthesis of macrocycle **1a** with an intraannular undecanedioxy bridge. a: Pd(PPh_3_)_2_Cl_2_, PPh_3_, CuI, piperidine, 84%; b: TBAF, THF, 94%; c: Pd(PPh_3_)_2_Cl_2_, CuI, 1,4-benzoquinone, piperidine, THF, 49%.

Scheme 1 shows the general synthetic approach towards the macrocycles with an intraannular flexible bridge. The tetraiodide **5**, which contains the two phenylene ring corners and the flexible alkyl template, as well as the naphthylene units **6** are synthesized independently (see Supporting Information File 1). Then **6** is attached to **5** in a fourfold Sonogashira–Hagihara reaction. To compensate the acetylene dimerization side reaction, the acetylene is added in 25% excess. Fluoride-induced removal of the silyl protecting groups yields the precursor **7**. With Pd(PPh_3_)_2_Cl_2_ and CuI as catalysts and 1,4-benzoquinone as oxidant, the precursor is finally intramolecular cyclized in THF/piperidine under high-dilution conditions by slowly adding (48 hours) a solution of the tetraacetylene to the reaction media.

**Figure 2:**
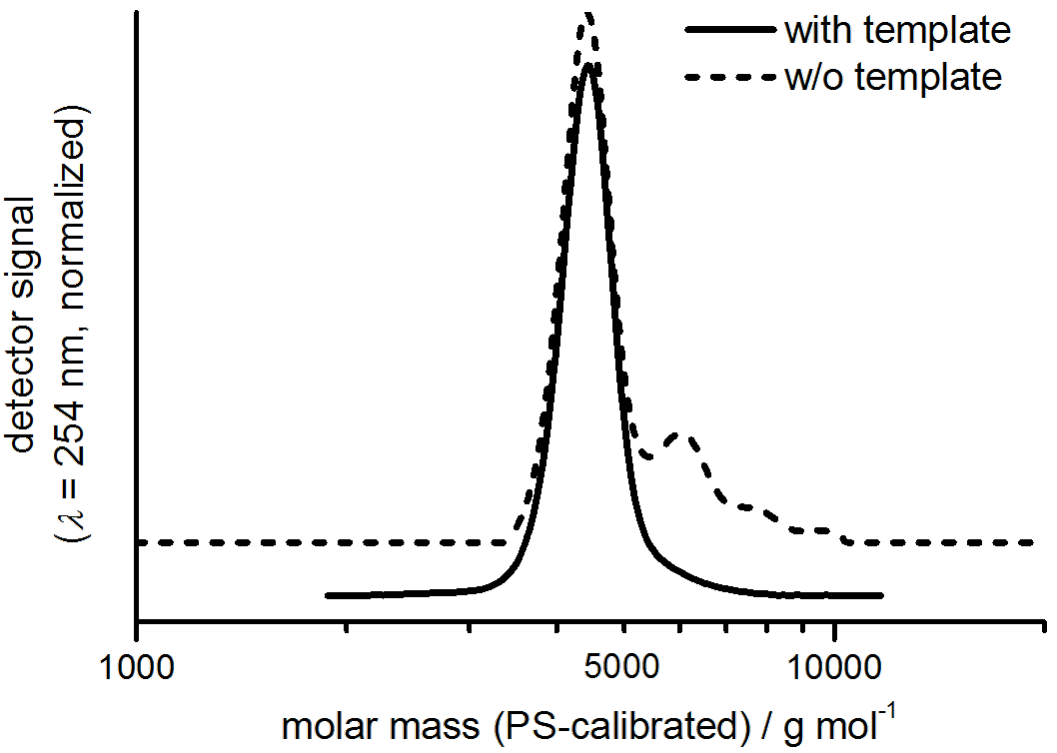
GPC elugrams of the crude product of the cyclization reaction of **1a** (—) and **1d** (- - -), respectively. For a better view the curves are vertically shifted.

Gel permeation chromatography (GPC) analysis of the crude product indicates that only few oligomeric byproducts are formed (Figure 2). With the aid of recycling GPC (recGPC) these impurities could be removed and **1a** is obtained in 49% yield. Following this synthetic route we synthesized the macrocycles **1a–c** as well as the macrocycles **3a** and **4a** with different side chains (Figure 1, see Supporting Information File 1 for experimental details and reference [11] for the preparation of **2a–d**).

#### Statistical macrocycle synthesis

**Scheme 2:**
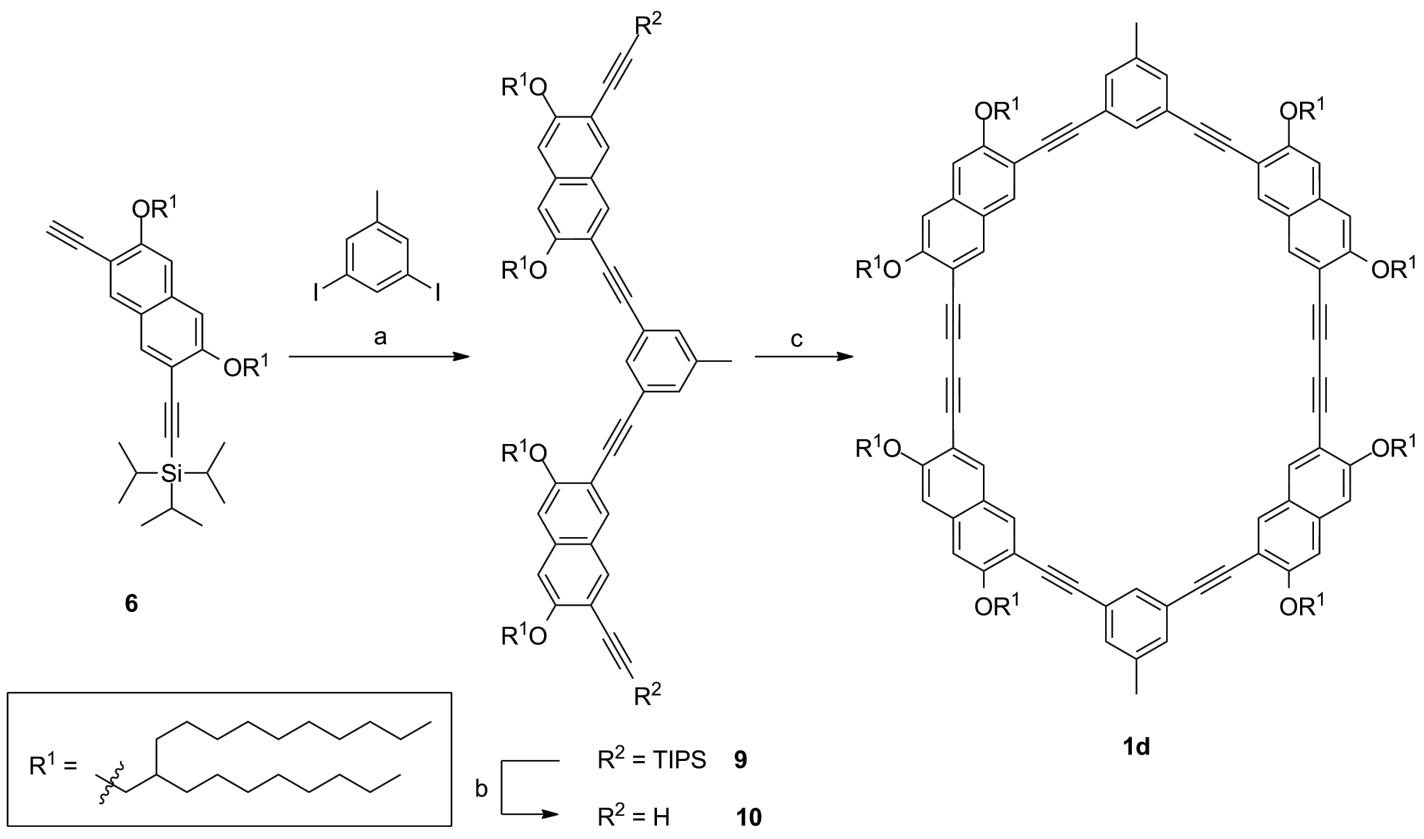
Synthesis of the template free macrocycle **1d**. a: Pd(PPh_3_)_2_Cl_2_, PPh_3_, CuI, piperidine, 98%; b: TBAF, THF, quant.; c: Pd(PPh_3_)_2_Cl_2_, CuI, 1,4-benzoquinone, piperidine, THF, 50 °C, 57%.

Macrocycle **1d** without intraannular substituents is obtained via statistical dimerization of the halfring **10** (Scheme 2). The half ring synthesis follows the above described approach and precursor **10** is cyclized under the same conditions as described for the template-mediated reaction. The GPC trace of the crude product shows significant amounts of oligomeric byproducts (Figure 2). Nevertheless, after purification by means of recGPC, macrocycle **1d** is obtained in a yield of 57%.

The comparison of the GPC traces of the crude products of **1a** and **1d** shows that in the intramolecular reaction less oligomers are formed than in the intermolecular reaction. However, the yields of the cyclization reactions do not differ significantly. That indicates that in the template-mediated cyclization side reactions cannot be completely suppressed. In the statistical half ring dimerization, the most important side reaction is the oligomerization of the half rings. Beside the desired dimers also trimers, tetramers, and other oligomers are formed, which can undergo further oligomerization reactions or may cyclize. These cyclic oligomers are still soluble and therefore they can be detected by GPC. In case of the template connected half rings, we assume that the oligomers formed through an intermolecular reaction cross-link, most likely form insoluble polymers, and are therefore not detected in the GPC analysis. The template has therefore two effects in the cyclization: (1) The terminal acetylenes are hold in proximity, thus, an *intra*molecular reaction is favored over an intermolecular reaction. (2) If an intermolecular coupling has occurred, the template leads to easily separable (insoluble) byproducts. However, the unexpected moderate yield in the template-directed synthesis suggests that the material may slowly decompose under the cyclization condition. Since other protocols (e.g., CuCl/CuCl_2_ in pyridine) did not give reproducible results, we tested whether high-dilution conditions can be omitted. For this purpose, we performed the cyclization towards macrocycle **1c** not under pseudo high-dilution conditions but by stirring a solution of the complete starting material of **1c** at once in THF, piperidine, Pd(PPh_3_)Cl_2_ and CuI as catalysts and 1,4-benzoquinone as oxidant for 3 h at 60 °C and obtained **1c** in 56% yield (after purification, see Supporting Information File 1). This result additionally emphasizes the potential of template-mediated reactions, which not only can be more efficient in terms of reducing byproducts but also paves the way towards a fast coupling protocol.

### Phase behavior

#### Thermal properties

**Figure 3:**
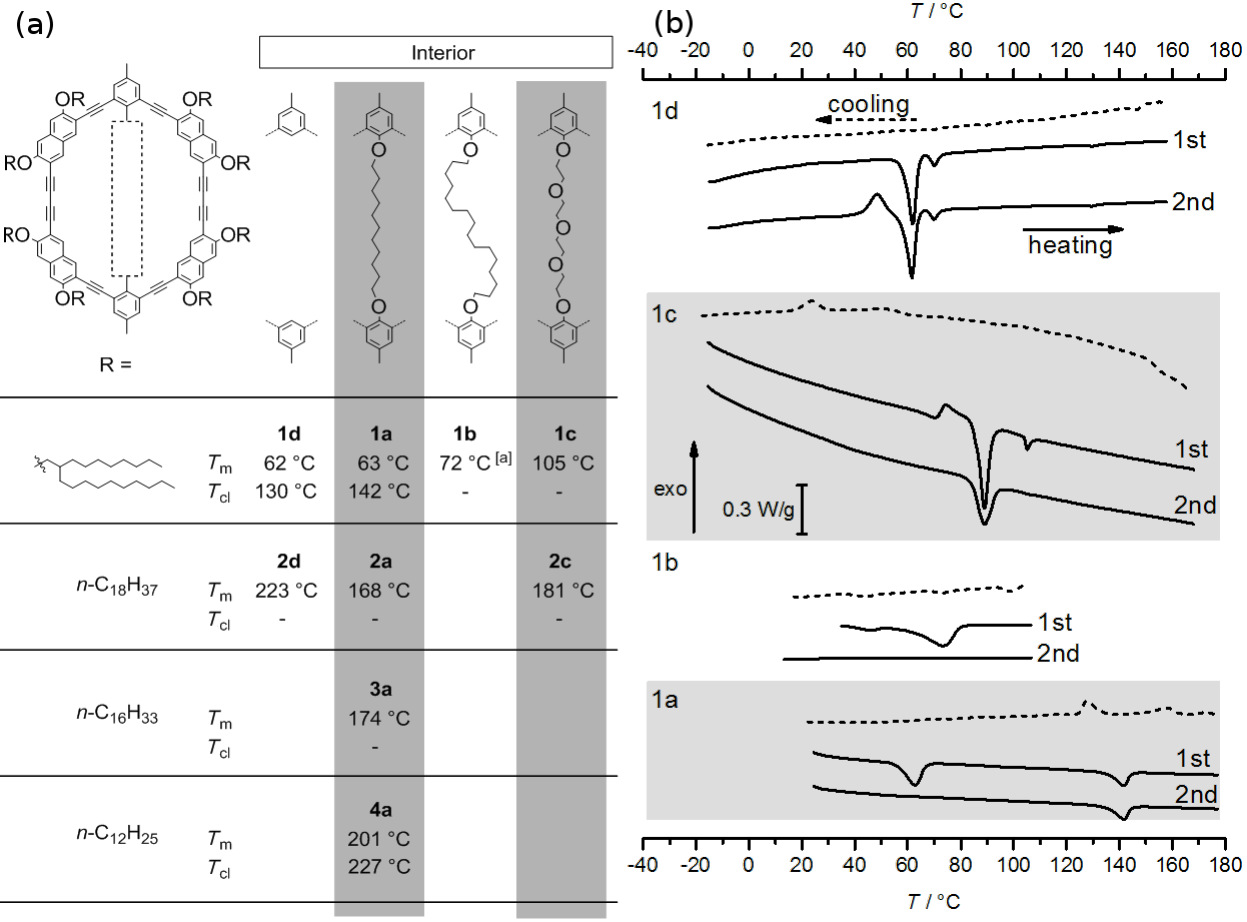
(a) Melting points (*T*_m_) and clearing points (*T*_cl_) of macrocycles with different interior. ^[a]^First heating. (b) DSC heat-flux curves of **1a–d** (10 K/min).

By means of polarized optical microscopy (POM) and differential scanning calorimetry (DSC) we investigated the thermal properties of the macrocycles **1–4**. The transition temperatures are shown in Figure 3 and listed in Table 1.

**Table 1:** Phase transitions and corresponding enthalpies of the discussed macrocycles.

Macrocycle (template)^a^	Transition temperatures [°C] (enthalpies [kJ/mol])^b^

**1a** (C11)	C 63 (20.4) Col_r_ 143 (10.5) I
**1b** (C16)	C 72 (33.8) I ^c^
**1c** (4EG)	C_1_ 89 (45.8) I,C_2_ 105 (3.1) I ^d^
**1d** (0)	C 62 (33.8) N_1_ 70 (4.3) N_2_ 130 (0.6) I ^e^
**2a** (C11)	C_1_ 51 (53.0) C_2_ 168 (38.5) I
**2c** (4EG)	C_1_ 66 (84.2) C_2_ 130 (1.1) C_3_181 (44.0) I
**2d** (0)	C_1_ 74 (128) C_2_ 223 (68.3) I
**3a** (C11)	C_1_ 51 (59) C_2_ 174 (40.9) I
**4a** (C11)	C 199 (40.8) LC 216 (0.4) I ^f^

^a^0 = no intraannular substituent; C11 = undecyl diether (–O(CH_2_)_11_O–); C16 = hexadecyl diether (–O(CH_2_)_16_O–); 4EG = tetraethylene glycol (–O(CH_2_CH_2_O)_4_–). ^b^Upon heating. C, C_2_, C_3_ = crystalline phase, I = isotropic phase, LC = liquid crystalline phase, N_1_, N_2_ = discotic nematic phase, Col_r_ = rectangular columnar phase. ^c^Only in the first scan. No crystallization upon cooling. ^d^The sample melts isotropic at 89 °C, except for few crystallites, which melt at 105 °C. ^e^In the second and following heating scans, right before the first transition a cold crystallization exotherm is observed (48 °C, 4.6 kJ/mol). ^f^The observed Schlieren-texture strongly indicated the formation of a nematic phase (see the Supporting Information File 1).

#### Extraannular substitution

It is well known that the periphery of discotic molecules generally dominates their thermal behavior. Shortening the side chains usually increases the melting point, whereas longer side chains or branched alkyl groups have the opposite effect [47,52–53]. However, when the side groups become too long or bulky, the compound melts isotropically and does not exhibit a mesophase [47]. By comparing the macrocycles **1a**, **2a**, **3a**, and **4a**, all with the same intraannular alkyl template the melting points decrease with increasing length of the extraannular alkyl chains. The lowest transition temperature is observed for **1a**, with branched side chains (Figure 3a), as it is also observed for other discotics. However, only two of the studied compounds (**1a** and **4a**) are able to form liquid crystalline (lc) mesophases indicating for the other compounds an unfavorable ratio of the core to the periphery size [54].

#### Intraannular substitution

The lc phase stability of **1a** within a wide temperature range (63 °C to 142 °C) stimulated the investigation of the derivatives **1b–d** to elucidate the influence of the intraannular substitution on the phase behavior. In addition, we addressed the question whether an interior change could lead to liquid crystallinity in **2**.

From the DSC and POM investigations in combination with the chemical structure of the compounds the following observations can be summarized: Although **1d** has an empty lumen it has a similar melting point (62 °C) as **1a** (63 °C), whose cavity crosses an alkyl bridge. Prolonging the intraannular alkyl chain length raises this transition temperature towards 72 °C (**1b**). If a polar template (**1c**) is used instead, the melting point reaches 89–105 °C (there are most probably two polymorphs, which melt at different temperatures). For comparison, compounds **2a–d** melt at 168 °C (**2a**), 181 °C (**2c**), and 223 °C (**2d**), respectively (Figure 3, Table 1).

**Figure 4:**
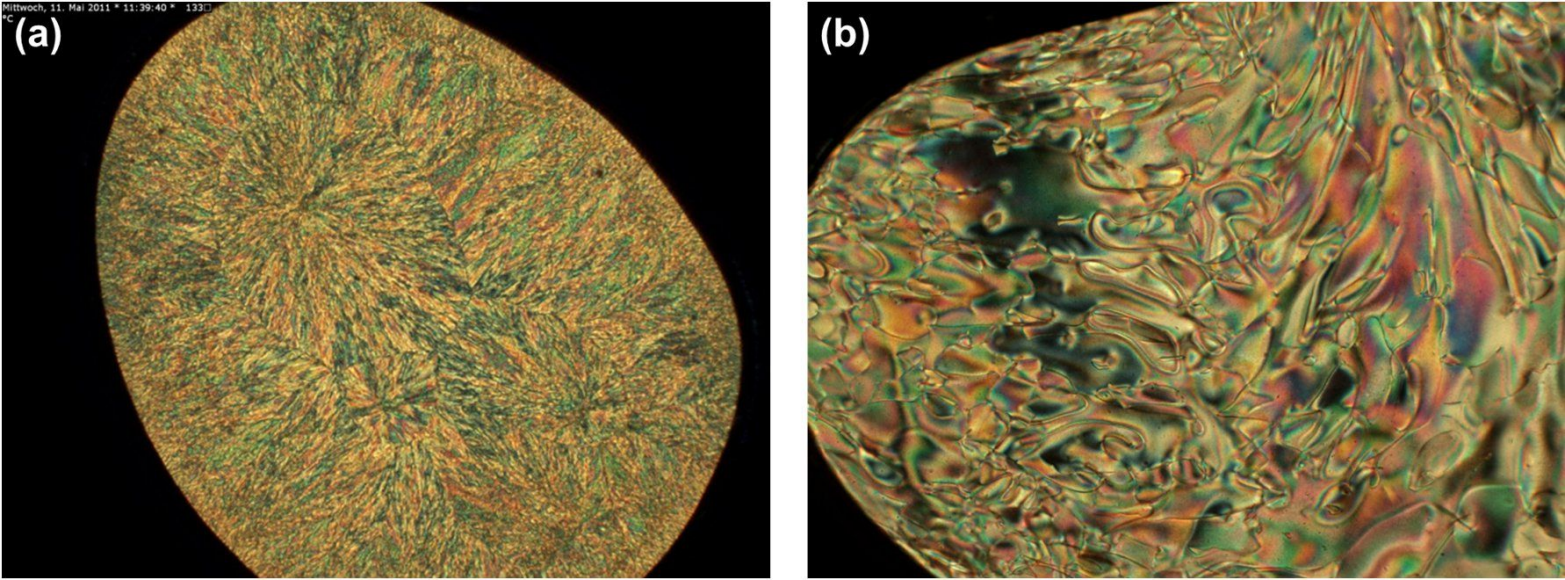
POM images of (a) **1a** (20×, 133 °C, upon cooling); (b) **1d** (20×, 84 °C, upon heating).

POM investigations indicate that **1a** and **1d** exhibit lc phases. Above the melting point, the sample of **1a** exhibits a fan shaped texture under the POM and shear tests indicate a wax-like viscosity of that phase (Figure 4a). The melt becomes isotropic when heated above 148 °C and the lc phase reappears upon cooling below 140 °C. **1d** forms in the temperature interval between 70 °C and 130 °C a birefringent lc phase with a characteristic Schlieren-texture (Figure 4b). Here, too, the lc phase reappears upon cooling from the isotropic melt (123 °C).

At lower temperatures, the mesophases of both, **1a** and **1d**, first solidify and slowly crystallize. The DSC results nicely confirm the POM observations. Corresponding endotherms for the melting and clearing points are visible in the thermograms of **1a** as well as **1d** (Figure 3b, Table 1). The sample of **1d** seems to melt at 62 °C (33 kJ/mol) into a nematic phase (N_1_) followed by a transition into another nematic phase (N_2_) at 70 °C (4.3 kJ/mol). The X-ray data of both phases are alike (see below). Upon cooling, no crystallization can be observed, either for **1a** or **1d**. However, for **1d** an exotherm followed by an endotherm is observed upon the second heating indicating crystallization and melting during the experiment. Clear hints on a stable mesophase could be obtained neither for **1b** nor for **1c**. From the DSC it seems that also **1c** exhibits an lc phase between 89 °C and 105 °C. However, from the POM and X-ray data we assume that **1c** forms at least two polymorphs which have different melting points. Unfortunately, their formation during the heating runs does not occur systematically but randomly.

These observations clearly show that also the intraannular substitution has a considerable influence on the thermal behavior of the macrocycles. The melting point increases in the order **1d** ≈ **1a** < **1b** < **1c**, showing the contribution of the intraannular template on the thermal behavior of the compounds. While the melting points of **1a** and **1d** are similar, the longer template in **1b** increases the melting point slightly and the additional interactions provided by the polar template in **1c** increase the melting point even further. However, the latter two compounds are not liquid crystalline. For **1b** can be assumed that the template is longer than the ring diameter and this leads to a loop in the molecule preventing the formation of an lc phase. For **1c**, the length of the template seems to be similar to the alkyl template in **1a**. However, the tendency of oligoethylene oxides to obtain a helical conformation [55] may fold the arylene–acetylene backbone into a boat conformation which is no longer a discotic mesogen. For the macrocycles **2a–d**, the melting points are rather high and clearly above the isotropization temperatures observed for **1a** and **1d**. The high melting point of **2d** might be explained by an interlocking of the molecules as a result of the empty interior of the rigid backbone [41]. A similar observation was also been made earlier on arylene–acetylene macrocycles. The fact that **1d** has a low melting point similar to **1a** although the interior is empty, is remarkable and prompted us to investigate **1a** and **1d** in more detail by X-ray diffraction to gain deeper insight into the structure of their liquid crystalline phases.

### X-ray diffraction

A sample of **1d** was kept in a glass capillary (Ø 1 mm) in a temperature-controlled heating stage and partially aligned in a magnetic field, another one and that of **1a** were surface aligned at the sample – air interface on a glass plate on a temperature controlled heating stage, all on slow cooling (~0.1 K/min) from the isotropic liquid. 2D patterns were recorded by an area detector HIStar (Siemens/Bruker) using Ni-filtered Cu Kα radiation.

**Figure 5:**
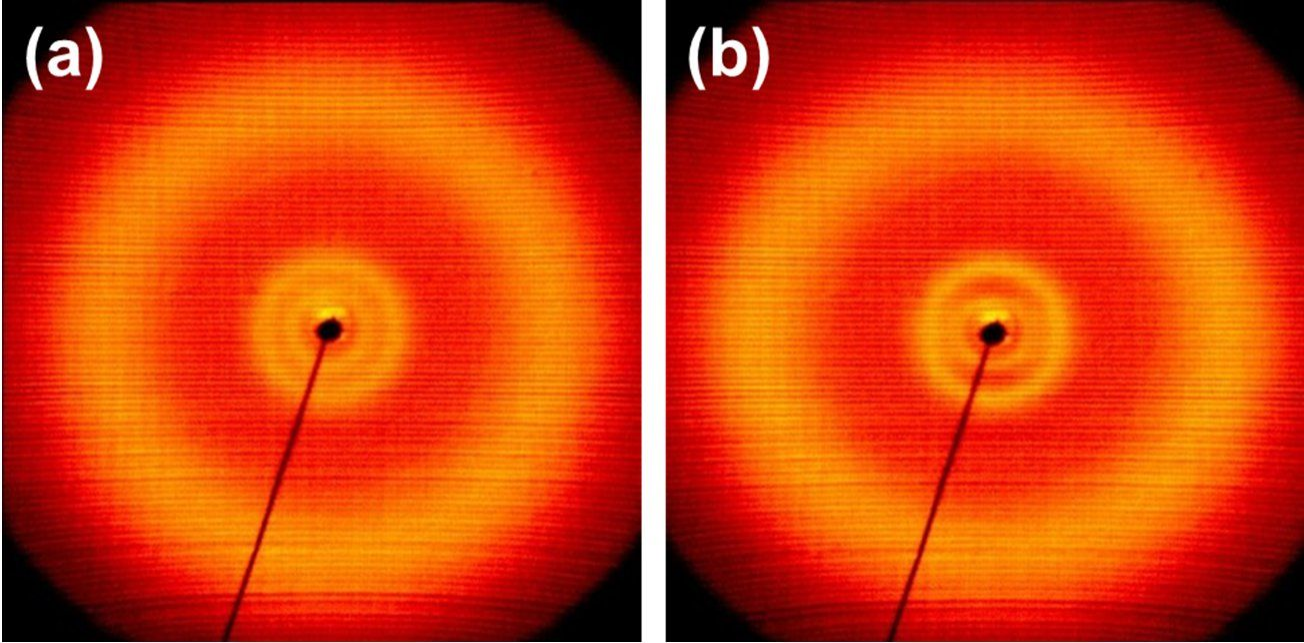
2D X-ray patterns for **1d:** (a) isotropic liquid at 160 °C, (b) partially aligned liquid crystalline phase at 120 °C on cooling. The magnetic field is parallel to the meridian of the pattern.

The patterns of **1d**, the compound without intraannular substitution, show in the isotropic liquid at 160 °C (Figure 5a) the usual outer diffuse scattering at about 4.6 Å characteristic for the average distance between the molecules along their short axes and between the side chains. In the small angle region there are two diffuse rings. These can be an indication of molecular aggregates which are already formed in the isotropic liquid [38]. The pattern slightly changes on cooling at the transition to the liquid crystalline phase and the sample becomes partially aligned in the magnetic field (Figure 5b and Supporting Information File 1 Figure S1 and Table S1). All reflections remain diffuse. Hence, it is a phase without long-range positional order and should be a kind of a nematic phase, in agreement with the optical textures. Obviously, similar clusters as in the isotropic phase are observed in the nematic phase. No changes of the X-ray pattern indicating a phase transition could be detected on heating above or on cooling below 70 °C (see Figure S1 in Supporting Information File 1) in contrast to those found for the nematic discotic (N_D_)–nematic columnar (N_C_) transition in liquid crystalline polymers [56] and for the N_D_–nematic lateral (N_L_) transition in liquid crystalline charge transfer complexes [57]. Neither magnetic nor surface alignment of the samples was sufficient to get evidence for or against a uniaxial nematic (N_u_)–biaxial nematic (N_b_) transition which has been extensively discussed in literature (see, e.g., [58]). The texture of the sample in the POM investigations did also not show significant changes like those observed for the N–N_x_ transition of liquid crystalline dimers and bent-core liquid crystals which has recently been identified as a nematic–twist bent nematic (N_TB_) transition (see, e.g., [59–60]). Therefore the nature of the phase change indicated by the DSC measurements could not be clarified yet.

**Figure 6:**
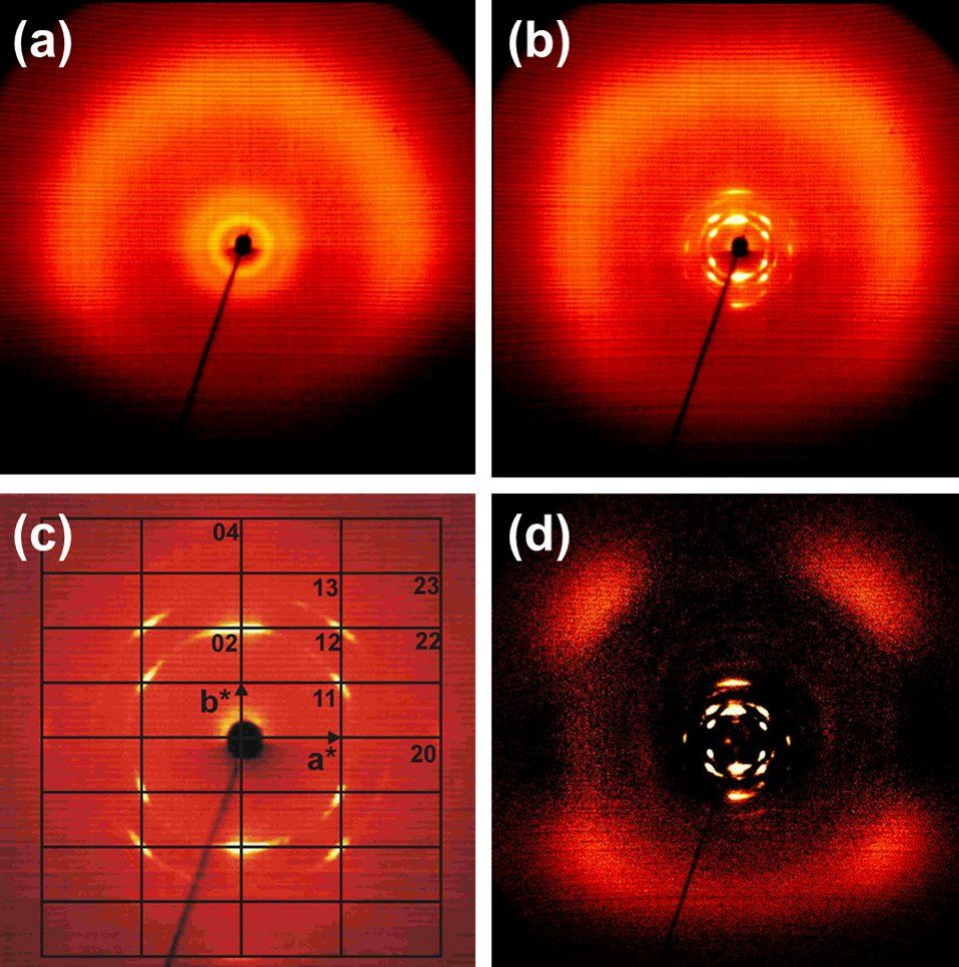
2D X-ray patterns for **1a:** (a) isotropic liquid at 150 °C, (b) columnar mesophase at 100 °C, surface aligned on cooling, (c) small angle region at 100 °C with reciprocal axes and indices for the 2D lattice of the columnar phase, (d) scattering at 150 °C subtracted from that at 100 °C to enhance the effect of the anisotropic distribution of the diffuse scattering.

The X-ray pattern for the isotropic liquid of **1a** (Figure 6a) closely resembles that of **1d**, only the intensity ratio for the two inner rings differs. The changes at the phase transitions are more dramatic as in case of **1d** (Figure 6b). Indeed, the outer scattering also shows a ring-like part and one with four maxima, but the latter are found 45° above and below the equator and the inner scattering splits into Bragg reflections (Figure 6d) which can be indexed on a rectangular two-dimensional lattice (plane group *p*2*gg*, reflections *h*0 only observed for *h* = 2*n* and 0*k* for *k* = 2*n*, cp. Figure 6c) with cell parameters *a* = 28.9 Å, *b* = 52.0 Å at 100 °C (Supporting Information File 1, Table S2) similar, for instance, to the 2D symmetry of the columnar lc phases of macrocycles reported in [40].

**Figure 7:**
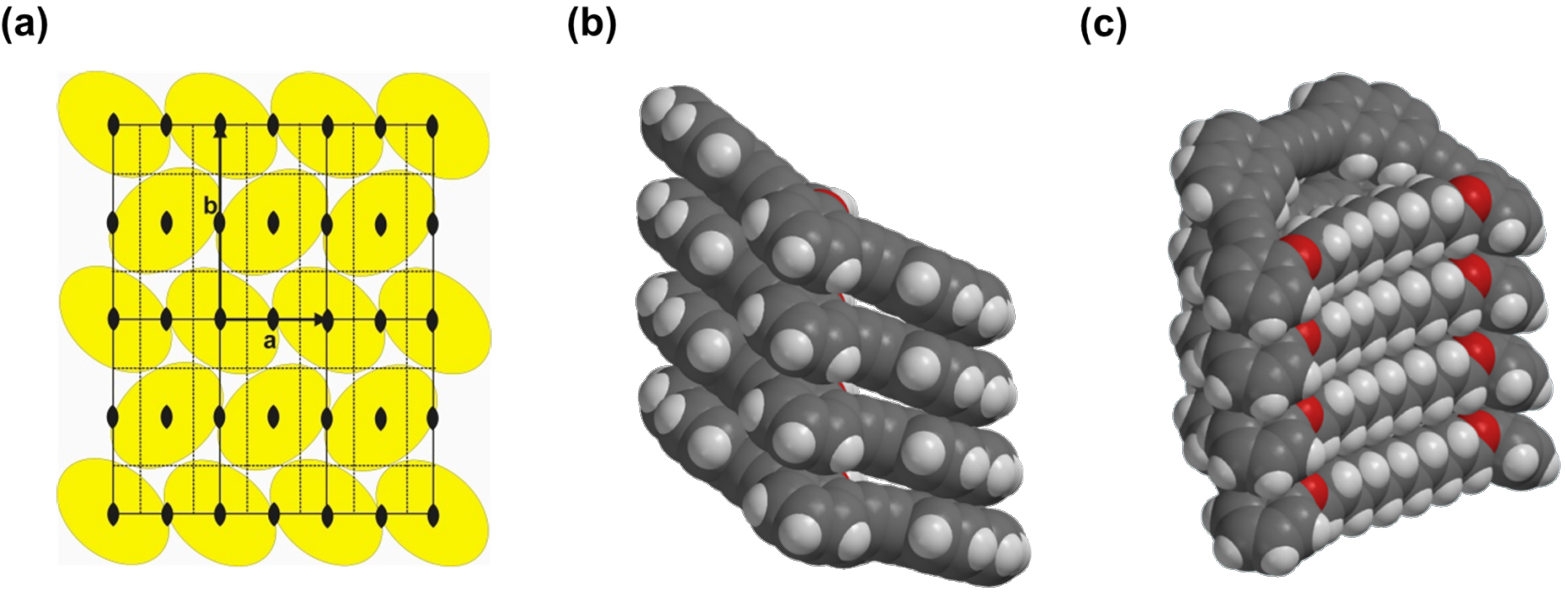
Model of the molecular packing in the columnar mesophase of **1a**: (a) 2D packing scheme for the columns in the liquid crystalline phase (plane group *p*2*gg*, arbitrary cross section of the columns to fit the symmetry); MMFF calculation (Spartan ’08) of a tetramer of the macrocycles; (b) suggested stacking of the macrocycles within one column (side chains omitted for clarity); (c) visualization of the intraannular alkyl chain packing within the columns.

A plausible packing for the molecules is a stacking of the macrocycles in columns, in which the mean planes of the cycles have a 45° tilt angle with respect to the columnar axes. The columns in turn are arranged in the 2D lattice described above. Assuming one molecule in the cross section of a column with C_2_ symmetry, the number of columns and hence of molecules in the cross section of the unit cell in this lattice is 2. For this packing model a reasonable density of 1.17 g/cm^3^ is calculated using an average stacking distance *h* = 4.6 Å / cos 45° = 6.5 Å of the macrocycles along the columnar axis (ρ_calc_ = *n*_cell_*M/*V*_cell_/A with a volume of an average 3D unit cell *V*_cell_ = *a* * *b* * *h* = 9768.2 Å^3^ and A = Avogadro constant). The assumed packing model also allows to understand the columnar phase stability. A MMFF calculation (Spartan ‘08) of a short column of tilted macrocycles shows a local minimum arrangement with a close contact between the intraannular alkyl chains (Figure 7). It might be the additional packing effect of the intraannular alkyl chains which stabilizes the columnar phase. A similar effect (although with polar intraannular ester groups) has been observed previously [40]. However, in that particular case, the analogue non-filled macrocycle is not liquid crystalline. The packing model in Figure 7 also indicates that the longer intraannular alkyl bridge of **1b** prevents a close packing of the rings and leads in this case even to the absence of the lc phase.

## Conclusion

In summary, shape-persistent macrocycles with intraannular bridges were synthesized by oxidative Glaser-coupling of the appropriate acetylenes. The bridges serve during the synthesis as a covalent template. Compounds with branched extraannular side chains exhibit in some cases liquid crystalline phases. Depending on the ring interior, either a nematic (empty interior) or a columnar phase (alkyl template) could be observed, as determined by differential scanning calorimetry, optical microscopy and X-ray scattering. It can be assumed that the additional van der Waals interaction between the stretched intraannular alkyl chains stabilize the packing of the rings on top of each other. When the alkyl bridge is longer than the ring interior or when an oligoether template crosses the ring, no lc behavior is observed. In both cases an induced non-planarity of the macrocycles is assumed.

## Supporting Information

File 1 Complete experimental details, including ^1^H and ^13^C NMR spectra.
