# Supplementary material for: The influence of intraannular templates on the liquid crystallinity of shape-persistent macrocycles
Source: Beilstein J Org Chem. 2014 Apr 23;10:910–20. doi: 10.3762/bjoc.10.89 (PMC4077431; doi:10.3762/bjoc.10.89)
Supplement: File 1 — Complete experimental details, including 1H and 13C NMR spectra. [file Beilstein_J_Org_Chem-10-910-s001.pdf]

Supporting Information

for

The influence of intraannular templates on the liquid crystallinity of shape-persistent macrocycles

Joscha Vollmeyer[1], Ute Baumeister*[2], and Sigurd Höger*[1]

Address: [1]Kekulé-Institut für Organische Chemie und Biochemie, Rheinische Friedrich-Wilhelms-Universität Bonn, Gerhard-Domagk-Str. 1, 53121 Bonn, Germany and [2] Institut für Chemie, Physikalische Chemie, Martin-Luther-Universität Halle-Wittenberg, Von-Danckelmann-Platz 4, 06120 Halle (Saale), Germany

Email: Ute Baumeister* - ute.baumeister@chemie.uni-halle.de; Sigurd Höger* - hoeger@uni-bonn.de

* Corresponding author

Complete experimental details, including ^{1}H and ^{13}C NMR spectra

Content

1 General methods 3

1.1 Materials and equipment 3

1.2 X-ray diffraction 4

2 Synthesis 6

2.1 Synthesis and characterization of building blocks **6** and **11–19** 6

2.2 Synthesis and characterization of macrocycles **1a**, **3a**, **4a** 21

2.3 Synthesis and characterization of macrocycles **1b** and **1c** 38

2.4 Synthesis and characterization of macrocycle **1d** 52

References 58

1 General methods

1.1 Materials and equipment

THF was dried over Na and benzophenone. Piperidine and pyridine were dried over CaH_2. The anhydrous solvents were distilled and stored under argon if necessary. Reagents were purchased at reagent grade from commercial sources and used without further purification. Substances **5**,[1] **11**,[2] **27**,[1] **30**,[3] and 3,5-diiodotoluoene[4] were prepared according to literature procedures. All air-sensitive reactions were carried out using standard Schlenk techniques under argon. Analytical thin-layer chromatography (TLC) was performed using precoated TLC plates (obtained from Macherey-Nagel, Alugram® SIL G/UV_{254}, 0.2 mm). Column chromatography was performed using silica gel 60 M (Macherey-Nagel, 0.04–0.063 mm, 230 - 400 mesh) as stationary phase. ^{1}H and ^{13}C NMR spectra were recorded on Bruker DPX 300 (^{1}H, 300 MHz; ^{13}C, 75 MHz), DPX 400 (^{1}H, 400 MHz; ^{13}C, 100 MHz), and DPX 500 (^{1}H, 500 MHz; ^{13}C, 125 MHz) spectrometers. Chemical shifts are reported as δ values (ppm) and referenced to residual ^{1}H or ^{13}C signals in deuterated solvents. Electron impact ionization (EI) mass spectroscopy (MS) was performed using a Finnigan ThermoQuest MAT 95 XL mass spectrometer. Matrix-assisted laser desorption/ionization (MALDI) MS was performed using a Bruker Daltonics autoflex II TOF/TOF. Electrospray ionization (ESI) MS data were recorded on a Bruker Daltonics ESI micrOTOF-Q instrument. Melting points were either determined using a Leica DMLB microscope with a hot stage and a home-built control unit, or by differential scanning calorimetry (DSC, see below). Gel permeation chromatography (GPC) was performed in THF (HPLC grade, stabilized with 2.5 ppm BHT) at room temperature. A Shimadzu recycling GPC system, equipped with an LC-20 AD pump, an SPD-20 A UV-detector, and a set of three preparative columns (obtained from PSS Polymer Standards Service, 10^3 Å, 5µ, 20 mm × 300 mm) was used for the purification of **1a–d**, and **3a**. The system was operated at a flow rate of 5 mL min^{-1}.

Differential scanning calorimetry (DSC) measurements were performed on a METTLER TOLEDO DSC 823^e with a HSS7-sensor under nitrogen atmosphere and liquid nitrogen cooling. The samples (2–4 mg of the respective compound) were placed inside of standard aluminum crucibles (V = 40 µL, METTLER TOLEDO) with perforated lids. The perforation is necessary to ensure gas exchange and pressure equalization. As reference an empty crucible was used. The heating and cooling cycles have been repeated at least twice until two following thermograms showed identical patterns.

Polarized optical microscopy (POM) was performed on a Leica DMLB microscope with a hot stage and a home-built control unit. Images were acquired with a Canimpex digital camera connected to a PC. To

distinguish liquids from solids, shear stresses were performed by pressing down the cover lid with a pincer.

1.2 X-ray diffraction

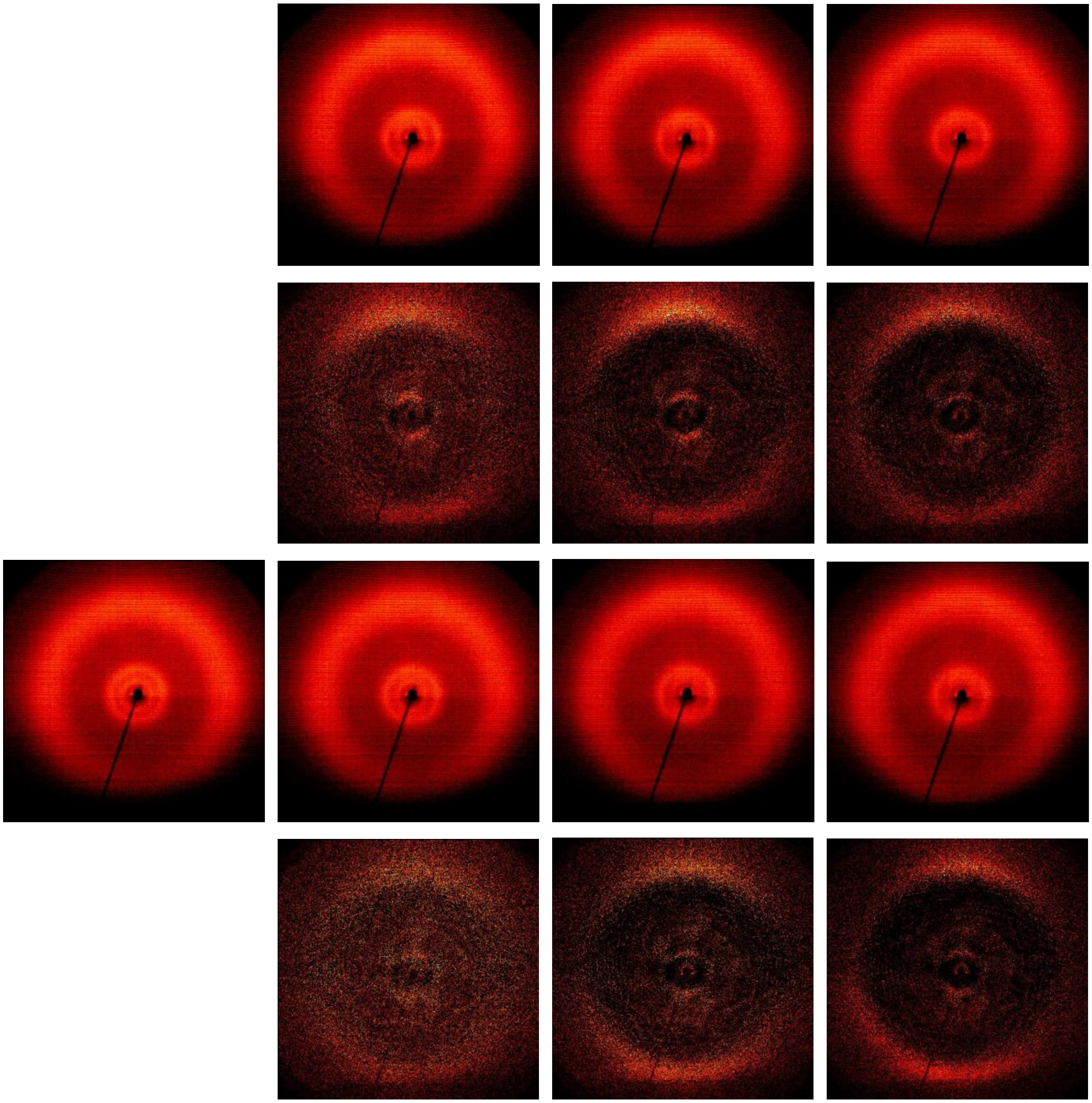

140 °C (isotropic liquid) **120°C (nematic lc)** **80°C (nematic lc)** **65 °C (nematic lc)**

Figure S1: 2D X-ray patterns for **1d** partially surface aligned at the sample – air interface. (1. + 2. row: on heating, 3. + 4. row: on cooling, 2. + 4. row: scattering at 140 °C subtracted from the above pattern to enhance the effect of the anisotropic distribution of the diffuse scattering)

Table S1: Observed angles $2\theta_{obs}$ (°) and *d* values d_{obs} (Å) for the intensity maxima of the diffuse X-ray scattering in the isotropic liquid (*T* = 160 °C) and in the l.c. phase (*T* = 120 °C) of **1d.** The outer scattering at 120 °C splits into two parts, the stronger one with the maxima on the equator of the pattern at $2\theta_{obs}$ = 19.2°, and the weaker, ring-like one at $2\theta_{obs}$ = 18.0°. The scattering at $2\theta_{obs}$ = 5.51° exhibits maxima on the equator and on the meridian of the pattern, whereas the four maxima of the innermost one at $2\theta_{obs}$ = 2.86° are situated 30° above and below the equator.

T	**$2\theta_{obs}$**	**d_{obs}**
160	3.057	28.9
	5.594	15.8
	19.0	4.7
120	2.861	30.9
	5.510	16.0
	18.0	4.9
	19.2	4.6

Table S2: Observed X-ray diffraction angles $2\theta_{obs}$ (°), *d* values d_{obs} (Å), Miller indices *h,k*, d values calculated from the parameters for a rectangular unit cell (plane group *p*2*gg, a* = 28.9 Å, *b* = 52.0 Å) d_{calc} at temperature *T* (°C) for **1a**.

T	**$2\theta_{obs}$**	**d_{obs}**	hk	**d_{calc}**	**d_{obs} - d_{calc}**
150	3.192*	27.7			
	5.702*	15.5			
	18.9*	4.7			
100	3.386	26.1	02	26.0	0.1
	3.487	25.3	11	25.3	0.1
	4.561	19.4	12	19.3	0.0
	5.965	14.8	13	14.9	0.0
	6.128	14.4	20	14.4	0.0
	6.804	13.0	04	13.0	0.0
	7.007	12.6	22	12.6	0.0
	19.4*	4.6			
	18.2*	4.9			

* maximum of the diffuse scattering

2 Synthesis

2.1 Synthesis and characterization of building blocks 6 and 11–19

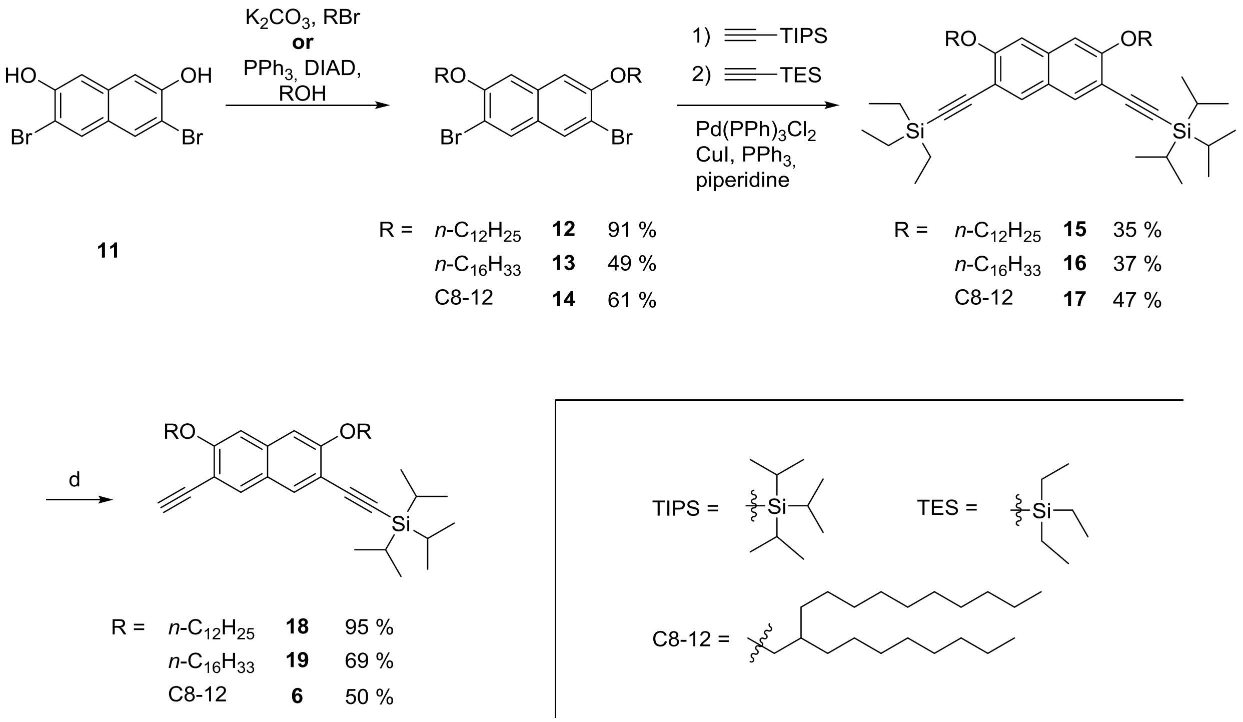

Scheme S1: Reaction pathway towards building blocks **6**, **18**, and **19**.

Synthesis of 2,7-dibromo-3,6-didodecoxynaphthalene (12). Under an argon atmosphere, 2,7-dibromo-3,6-dihydroxynaphthalene **11** (0.50 g, 1.57 mmol),[2] 1-bromododecane (0.80 g, 3.21 mmol), potassium carbonate (1.74 g, 12.56 mmol), and potassium iodide (6.4 mg, 0.04 mmol) were dissolved in dry DMF (10 ml). After stirring for 18.5 h at 60 °C the solution was allowed to cool down to room temperature and diluted with dichloromethane and water. The organic layer was separated and washed with water (2×), aqueous acetic acid (10%, v/v, 2×), water, aqueous NaOH (10%), water and brine. After drying over $MgSO_4$ the solution was concentrated under reduced pressure. To the residue methanol was added and the precipitate was filtered off and dried in vacuum. The product was obtained as a colorless solid (0.93 g, 1.42 mmol, 91%, R_f = 0.9, petroleum ether : CH_2Cl_2 = 1:1). M.p. 70 °C. ^{1}H NMR ($CDCl_3$, 400 MHz, 298 K): δ [ppm] = 7.85 (s, 2H), 6.99 (s, 2H), 4.09 (t, J = 6.5 Hz, 4H), 1.97–1.84 (m, 4H), 1.59–1.48 (m, 4H), 1.27 (s, 32H), 0.88 (t, J = 6.9 Hz, 6H). ^{13}C NMR (100 MHz, $CDCl_3$, 298 K): δ [ppm] = 153.92, 134.09, 130.99, 125.19,111.96, 106.59, 69.25, 32.07, 29.82, 29.80, 29.75, 29.72, 29.51, 29.48, 29.13, 26.19, 22.84, 14.27. MS EI (70 eV): m/z (%): 654.3 (71) [^{79,81}M]$^{+\cdot}$, 486.1 (7) [^{79,81}M – $C_{12}H_{24}$]$^{+\cdot}$, 317.9 (100) [^{79,81}M – $C_{24}H_{48}$]$^{+\cdot}$; $C_{34}H_{54}Br_2O_2$ requires 654.25.

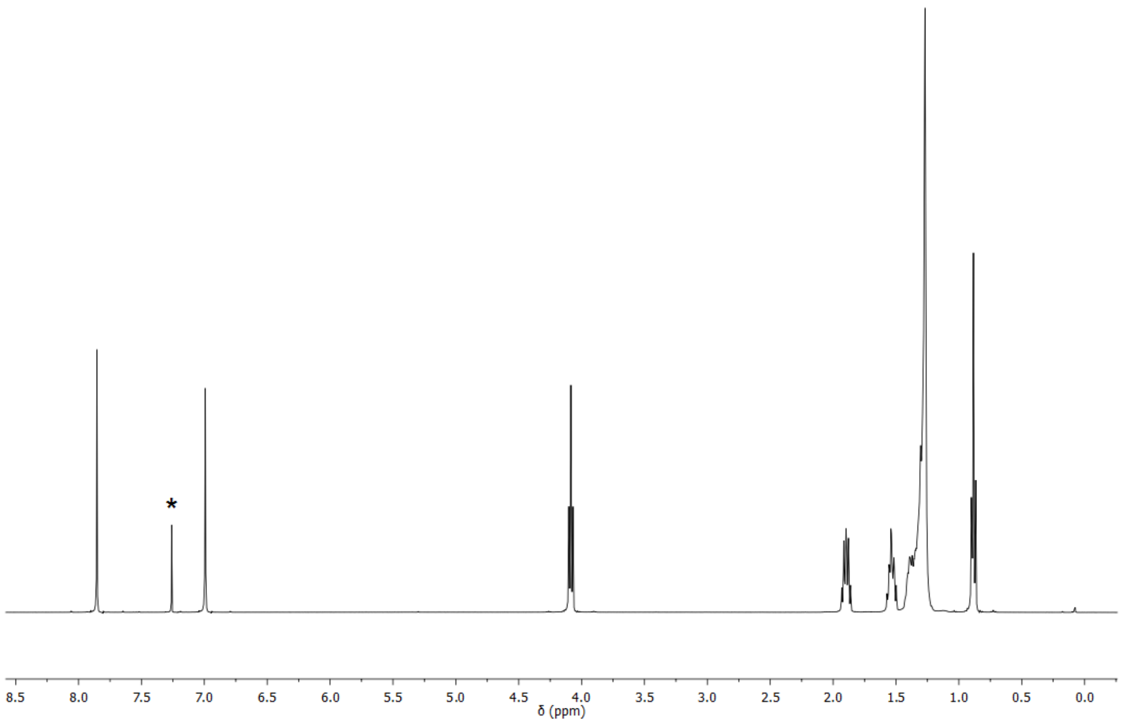

Figure S2: ^{1}H NMR (400 MHz, $CDCl_3$) of **12** (* = NMR solvent residual peak).

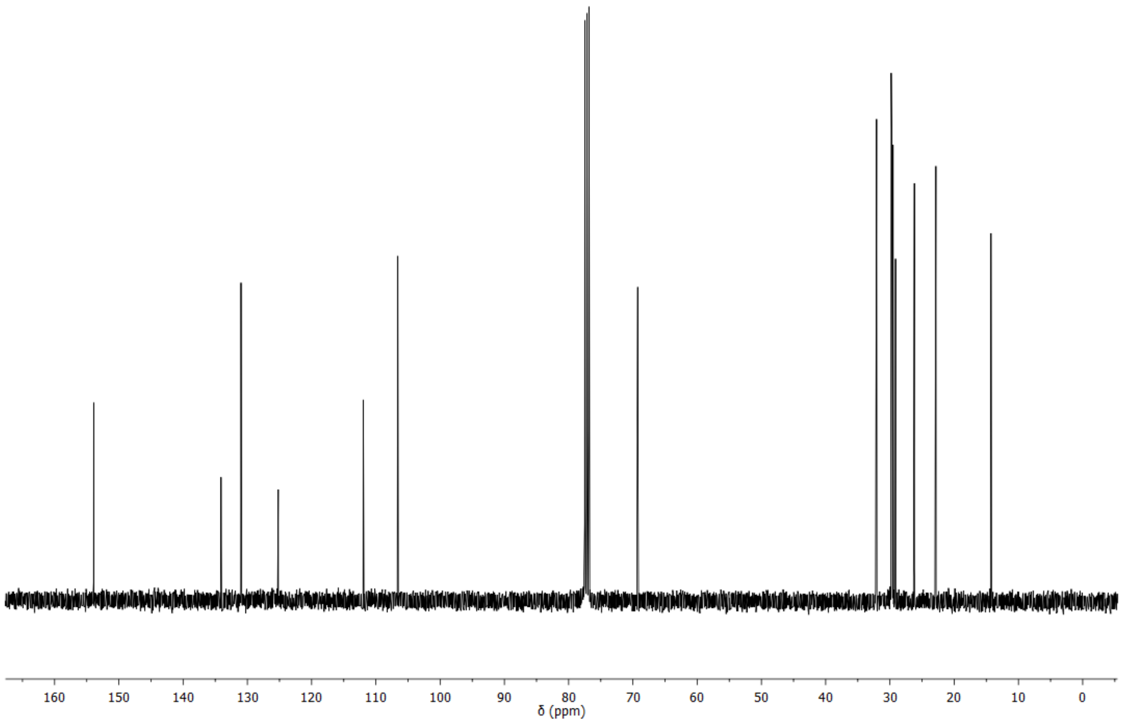

Figure S3: ^{13}C NMR (100 MHz, $CDCl_3$) of **12** (* = NMR solvent residual peak).

Synthesis of 2,7-dibromo-3,6-dihexadecoxynaphthalene (13). Under an argon atmosphere, 2,7-dibromo-3,6-(dihydroxy)naphthalene **11** (1.04 g, 3.27 mmol), 1-bromo hexadecane (2.00 g, 6.54 mmol), potassium carbonate (3.64 g, 26.2 mmol), and potassium iodide (13 mg, 0.08 mmol) were dissolved in dry DMF (20 ml). After stirring for 18.5 h at 65 °C the solution was allowed to cool to room temperature and was diluted with chloroform and water. The organic layer was separated and washed with aqueous acetic acid (10%, v/v, 3×), water (3×), aqueous NaOH (10%, 2×), and brine (3×). After drying over $MgSO_4$ the solvent was removed under reduced pressure and the residue was dissolved in dichloromethane. To the solution methanol was added and the precipitate was filtered off and dried in vacuum. The product was redissolved and filtered through a short silica column (silica gel, petroleum ether / DCM = 1 : 1; R_f = 1) and the resulting product was recrystallized from 2-propanol to yield **13** as a slightly yellow solid (1.20 g, 1.56 mmol, 49%). Mp. 77 °C. ^{1}H NMR ($CDCl_3$, 300 MHz, 298 K): δ [ppm] = δ = 7.86 (2H, s), 7.00 (2H, s), 4.09 (4H, t), 1.90 (4H, m), 1.53 (4H, m), 1.45 - 1.18 (48H, m), 0.88 (6H, t). ^{13}C NMR (75 MHz, $CDCl_3$, 298 K): δ [ppm] = 153.76, 133.93, 130.83, 125.03, 111.81, 106.43, 69.10, 31.92, 29.69, 29.66, 29.59, 29.56, 29.36, 29.33, 28.98, 26.04, 22.69, 14.11.

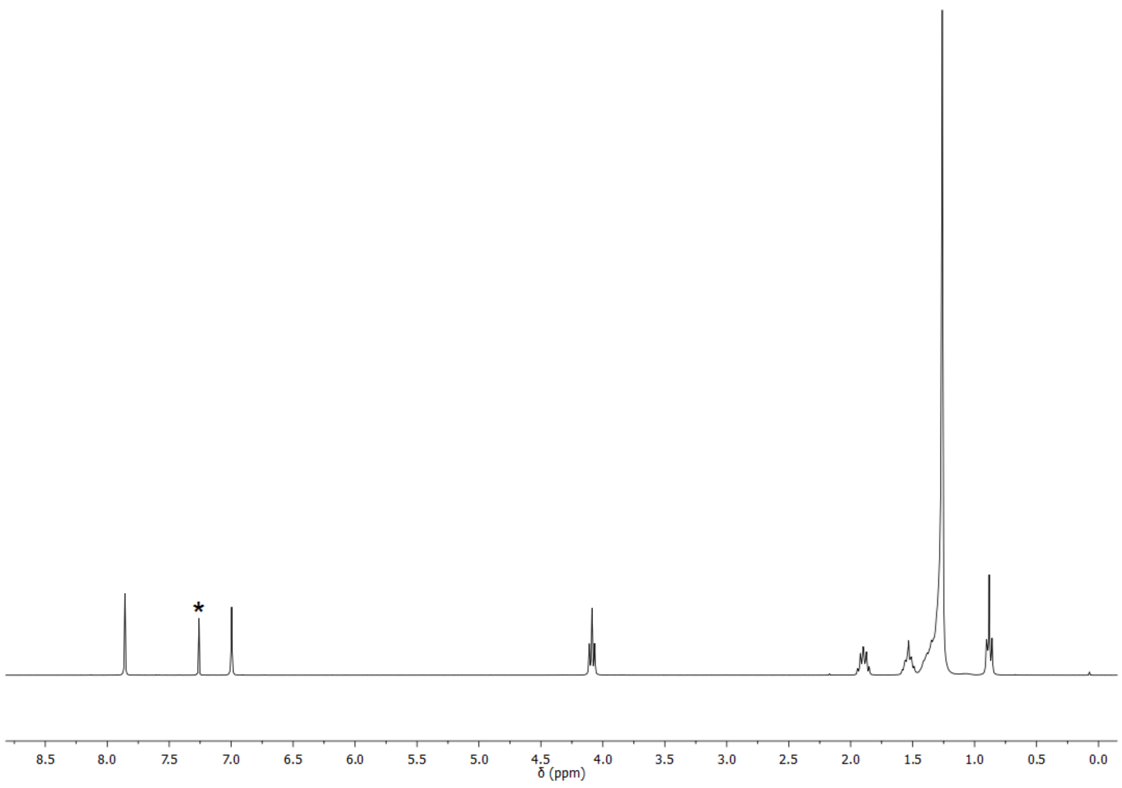

Figure S4: ^{1}H NMR (300 MHz, $CDCl_3$) of **13** (* = NMR solvent residual peak).

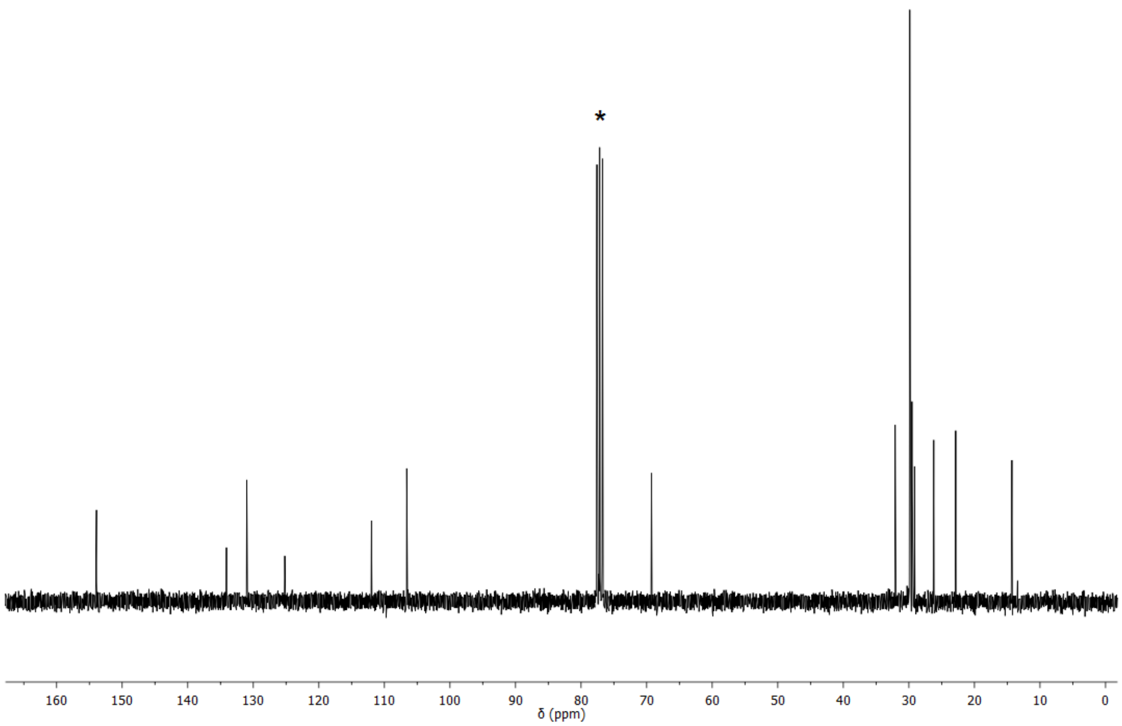

Figure S5: ^{13}C NMR (75 MHz, $CDCl_3$) of **13** (* = NMR solvent residual peak).

Synthesis of 2,7-dibromo-3,6-bis([2-octyl]dodecoxy)naphthalene 14. Under an argon atmosphere, 2,7-dibromo-3,6-(dihydroxy)naphthalene **11** (1.00 g, 3.15 mmol),[2] PPh_3 (2.47 g, 9.44 mmol), and 2-octyldodecanol (2.30 g, 7.70 mmol) were dissolved in dry THF (10 ml). The reaction mixture was cooled with an ice bath and DIAD (1.95 g, 9.64 mmol) was added drop-wise. After stirring for 42 h at room temperature the solution was diluted with dichloromethane and water was added. The organic layer was separated and washed with water (3×) and with brine. After drying over $MgSO_4$ the solvent was evaporated. The product was purified by column chromatography (silica gel, petroleum ether / DCM = 15 : 1; R_f = 0.66, petroleum ether) yielded **14** as a colorless oil (1.70 g, 1.93 mmol, 61%). ^{1}H NMR ($CDCl_3$, 400 MHz, 298 K): δ [ppm] = 7.85 (s, 2H), 7.00 (s, 2H), 3.96 (d, J = 5.5 Hz, 4H), 1.98–1.83 (m, 2H), 1.65–1.18 (m, 64H), 0.95–0.80 (m, 12H). ^{13}C NMR (75 MHz, $CDCl_3$, 298 K): δ [ppm] = 154.11, 134.12, 130.93, 125.14, 112.07, 106.42, 71.99, 37.99, 32.07, 31.58, 30.16, 29.83, 29.81, 29.77, 29.74, 29.51, 29.50, 27.01, 22.84, 14.26. MS ESI (10 eV): m/z (%): 901.5 (49) $[^{79,81}M + Na]^{+\cdot}$, 877.5 (9) $[^{79,79}M + H]^{+\cdot}$; $C_{50}H_{86}Br_2O_2$ requires 876.50.

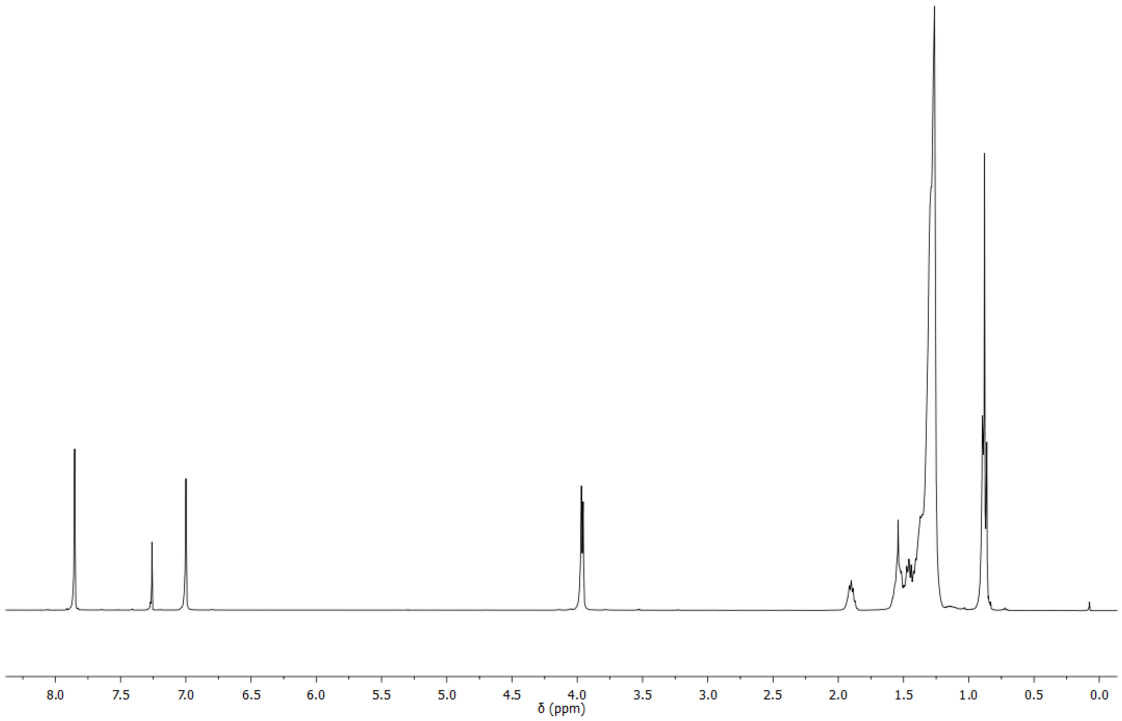

Figure S6: ^{1}H NMR (400 MHz, $CDCl_3$) of **14** (* = NMR solvent residual peak).

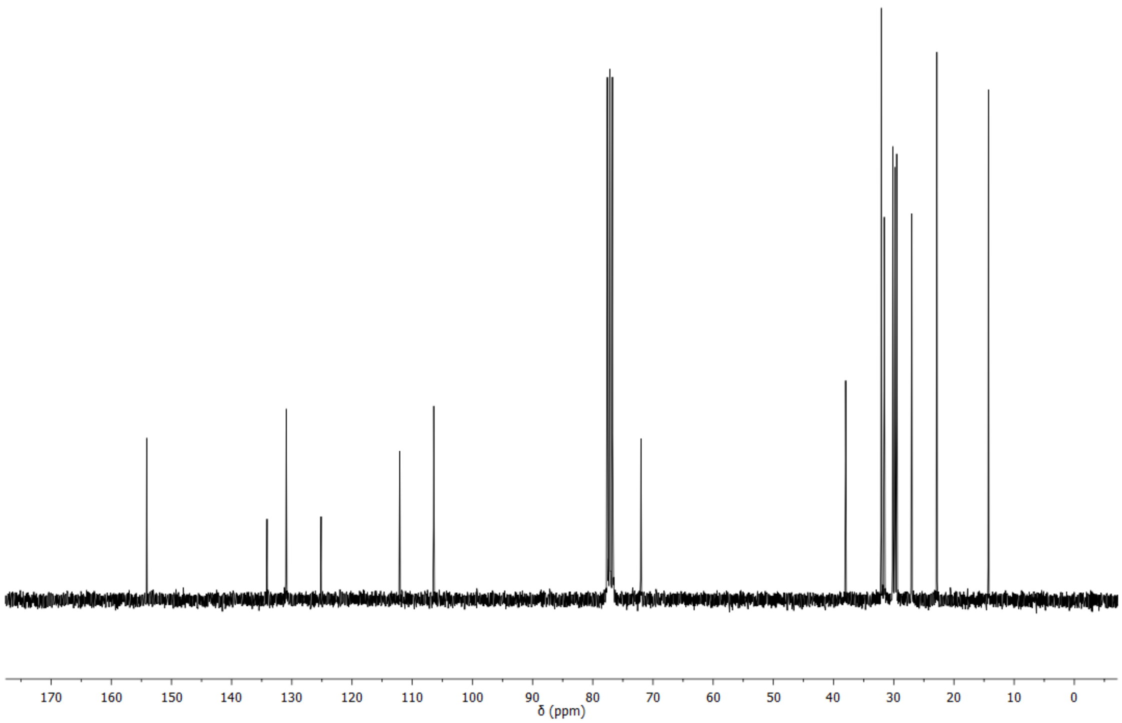

Figure S7: ^{13}C NMR (75 MHz, $CDCl_3$) of **14** (* = NMR solvent residual peak).

Synthesis of 15. 12 (981 mg, 1.50 mmol), $Pd(PPh_3)Cl_2$ (17 mg, 24 µmol), PPh_3 (25 mg, 95 µmol), and CuI (7 mg, 37 µmol) were placed in a Schlenk tube under an argon atmosphere. The compounds were dissolved in dry piperidine (7 mL) and TIPS-acetylene (333 mg, 1.83 mmol) was added dropwise. The reaction mixture was heated to 60 °C and stirred for 22.5 h. Then, TES-acetylene (392 mg, 2.79 mmol) was added and the reaction mixture was stirred for additional 5 h at 60 °C. The mixture was allowed to cool to room temperature and diluted with dichloromethane. The organic phase was washed with water (2×), aqueous acetic acid (10%, v/v, 2×), aqueous NaOH (10%), and brine. After drying over $MgSO_4$ the solvent was evaporated. The product was purified by column chromatography (silica gel, petroleum ether : CH_2Cl_2 = 15 : 1, R_f = 0.26) yielding **15** as a colorless oil (428 mg, 0.525 mmol, 35%). ^{1}H NMR (400 MHz, $CDCl_3$, 298 K): δ [ppm] = 7.80 (s, 1H), 7.79 (s, 1H), 6.90 (s, 2H), 4.11–4.01 (m, 4H), 1.92–1.79 (m, 4H), 1.60–1.49 (m, 4H), 1.42–1.21 (m, 32H), 1.20–1.14 (m, 21H), 1.08 (t, J = 7.9Hz, 9H), 0.88 (t, J = 6.9Hz, 6H), 0.70 (q, J =7.9Hz, 6H).

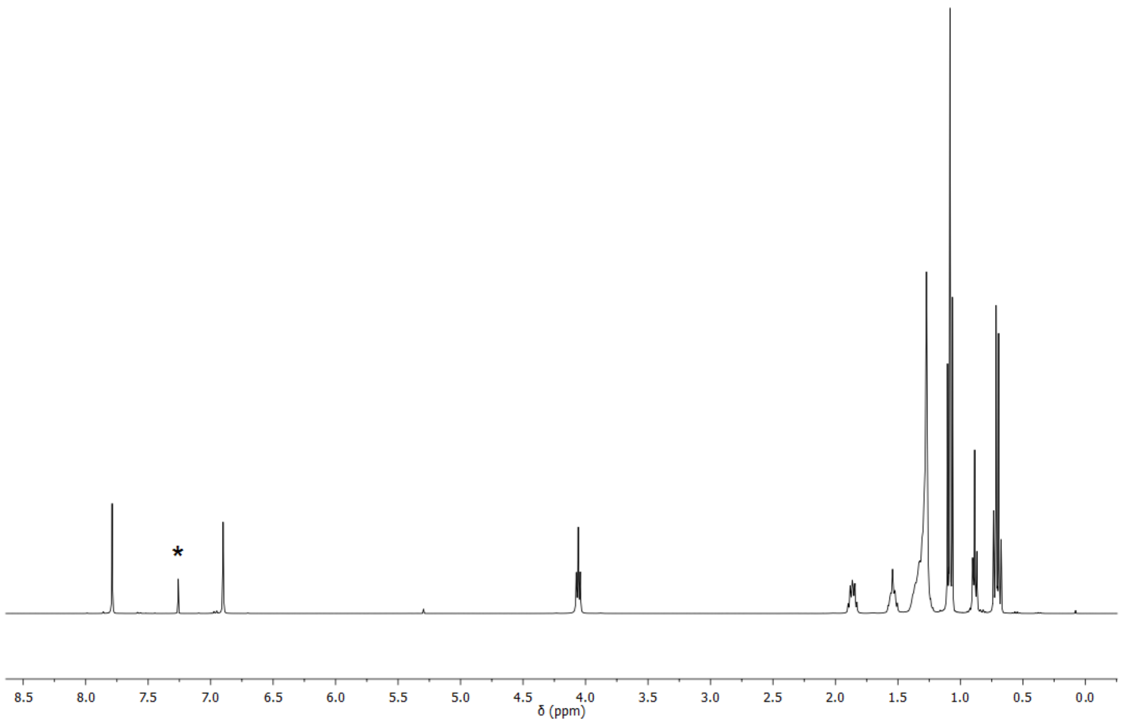

Figure S8: ^{1}H NMR (400 MHz, $CDCl_3$) of **15** (* = NMR solvent residual peak).

Synthesis of 16. 13 (1.00 g, 1.30 mmol), $Pd(PPh_3)Cl_2$ (14 mg, 19.9 µmol), PPh_3 (14 mg, 53.4 µmol), and CuI (6.2 mg, 32.6 µmol) were placed in a Schlenk tube under an argon atmosphere. The compounds were dissolved in dry piperidine (10 mL) and TIPS-acetylene (250 mg, 1.37 mmol) was added dropwise. The reaction mixture was heated to 60 °C and stirred for 2 h. Then, TES-acetylene (220 mg, 1.57 mmol) was added and the reaction mixture was stirred for additional 20 h at 60 °C. The mixture was allowed to cool to room temperature before it was diluted with dichloromethane. The organic phase was washed with water, aqueous acetic acid (10%, v/v), aqueous NaOH (10%), and brine. After drying over $MgSO_4$ the solvent was evaporated. The product was purified by column chromatography (silica gel, petroleum ether : CH_2Cl_2 = 10 : 1, R_f = 0.30) yielding **16** as a colorless oil (442 mg, 0.477 mmol, 37%). ^{1}H NMR (500 MHz, $CDCl_3$, 298 K): δ [ppm] 7.80 (s, 1H), 7.78 (s, 1H), 6.90 (s, 2H), 4.11–3.98 (m, 4H), 1.92–1.78 (m, 4H), 1.62–1.47 (m, 8H), 1.43–1.20 (m, 44H), 1.19–1.13 (m, 21H), 1.08 (t, J = 7.9 Hz, 9H), 0.88 (t, J = 7.0 Hz, 6H), 0.70 (q, J = 7.9 Hz, 6H). ^{13}C NMR (125 MHz, $CDCl_3$, 298 K): δ [ppm] = 158.32, 158.17, 135.84, 133.74, 133.72, 122.74, 112.73, 112.43, 105.17, 105.10, 103.32, 102.67, 95.63, 94.48, 68.57, 32.08, 29.86, 29.84, 29.68, 29.52, 29.50, 29.44, 26.46, 26.39, 22.85, 18.89, 14.27, 11.58, 7.72, 4.69. MS (ESI, 10 eV): m/z (%) = 949.8 (100) $[M + Na]^{+\cdot}$; $C_{61}H_{106}O_2Si_2$ requires 926.77.

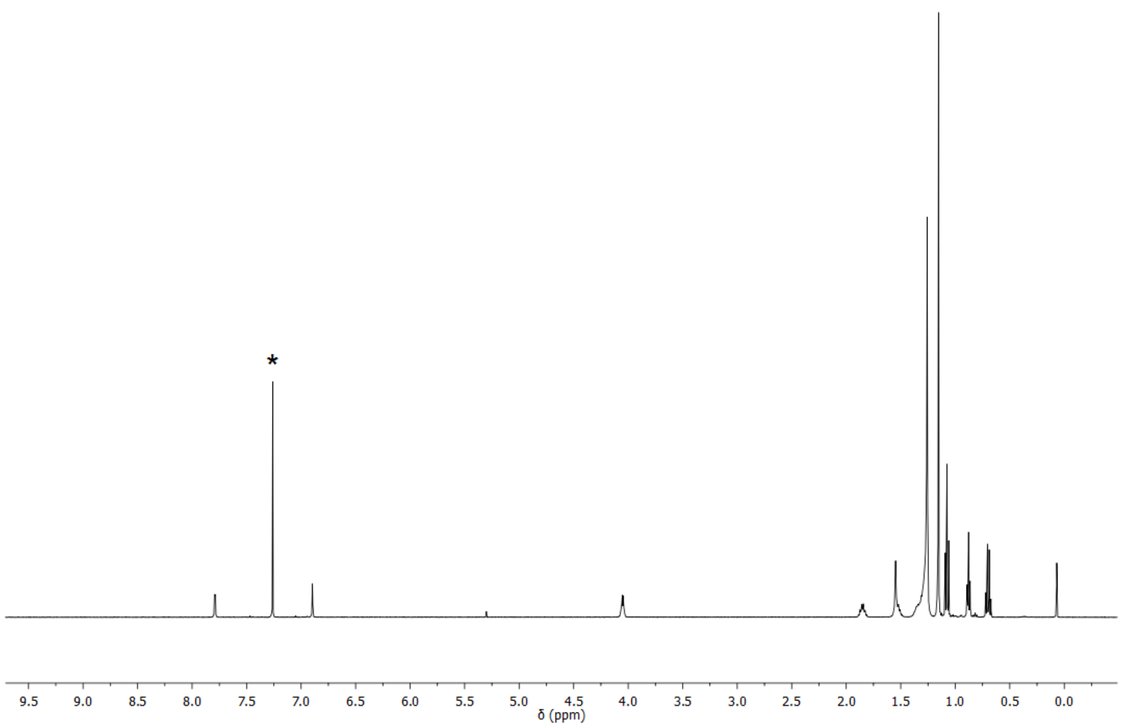

Figure S9: ^{1}H NMR (500 MHz, $CDCl_3$) of **16** (* = NMR solvent residual peak).

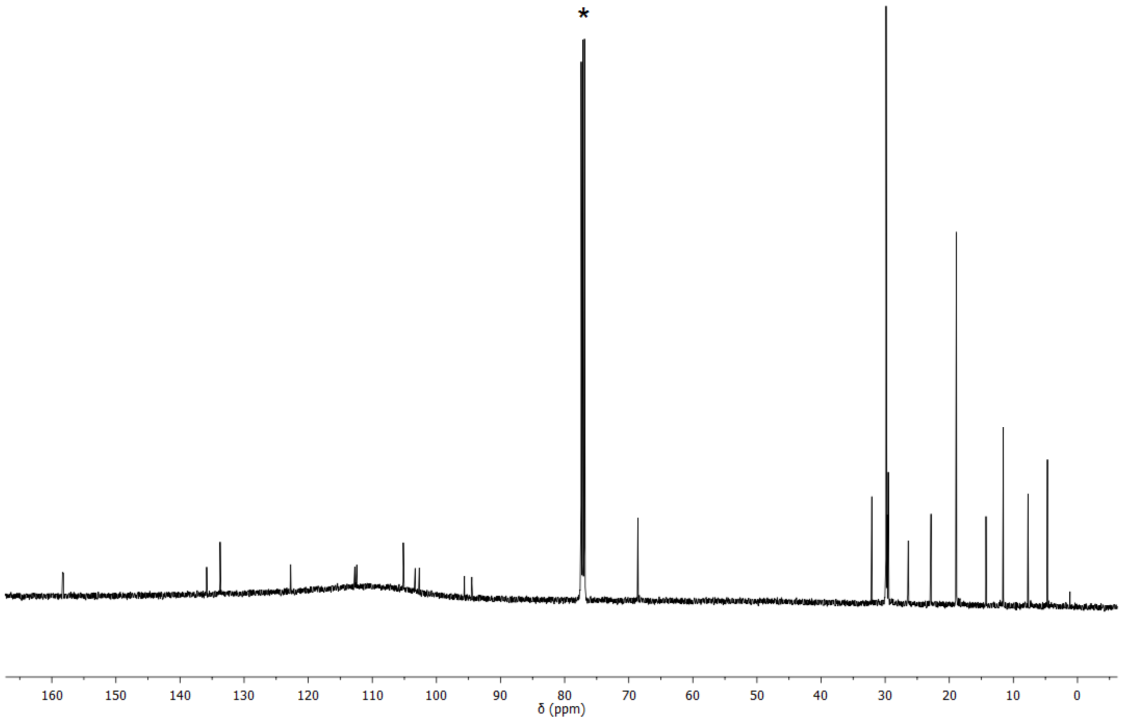

Figure S10: ^{13}C NMR (125 MHz, $CDCl_3$) of **16** (* = NMR solvent residual peak).

Synthesis of 17. 14 (1.01 g, 1.15 mmol), $Pd(PPh_3)Cl_2$ (13.3 mg, 18.9 µmol), PPh_3 (12.2 mg, 46.5 µmol), and CuI (5.2 mg, 27.2 µmol) were placed in a Schlenk tube under an argon atmosphere. The compounds were dissolved in dry piperidine (5 mL) and TIPS-acetylene (244 mg, 1.34 mmol) was added dropwise. The reaction mixture was heated to 60 °C and stirred for 1.5 h. Then, TES-acetylene (243 mg, 1.73 mmol) was added and the reaction mixture was stirred for additional 20 h at 60 °C. The mixture was allowed to cool to room temperature before it was diluted with dichloromethane. The organic phase was washed with water, aqueous acetic acid (10%, v/v), aqueous NaOH (10%), and brine. After drying over $MgSO_4$ the solvent was evaporated. The product was purified by column chromatography (silica gel, petroleum ether : CH_2Cl_2 = 19 : 1, R_f = 0.35) yielding **17** as a colorless oil (564 mg, 0.542 mmol, 47%). ^{1}H NMR (500 MHz, $CDCl_3$, 298 K): *δ* [ppm] = 7.80 (s, 1H), 7.79 (s, 1H), 6.91 (s, 2H), 3.97–3.92 (m, 4H), 1.91–1.80 (m, 2H), 1.62–1.49 (m, 4H), 1.49–1.38 (m, 4H), 1.38–1.20 (m, 56H), 1.16 (s, 21H), 1.07 (t, *J* = 7.9 Hz, 9H), 0.88 (t, *J* = 7.0 Hz, 12H), 0.70 (q, *J* = 7.9 Hz, 6H). ^{13}C NMR (125 MHz, $CDCl_3$, 298 K): *δ* [ppm] = 158.36, 158.31, 135.87, 134.02, 133.80, 122.66, 112.66, 112.42, 104.94, 104.92, 103.39, 102.64, 95.38, 94.18, 71.01, 38.26, 32.07, 32.06, 31.38, 31.30, 30.25, 30.23, 29.87, 29.86, 29.82, 29.80, 29.53, 29.52, 27.06, 27.04, 22.84, 18.92, 14.27, 11.57, 7.74, 4.67. MS (MALDI-TOF, DCTB): *m/z* (%) = 1038.9 (100) $[M]^{\cdot+}$; $C_{69}H_{122}O_2Si_2$ requires 1038.90.

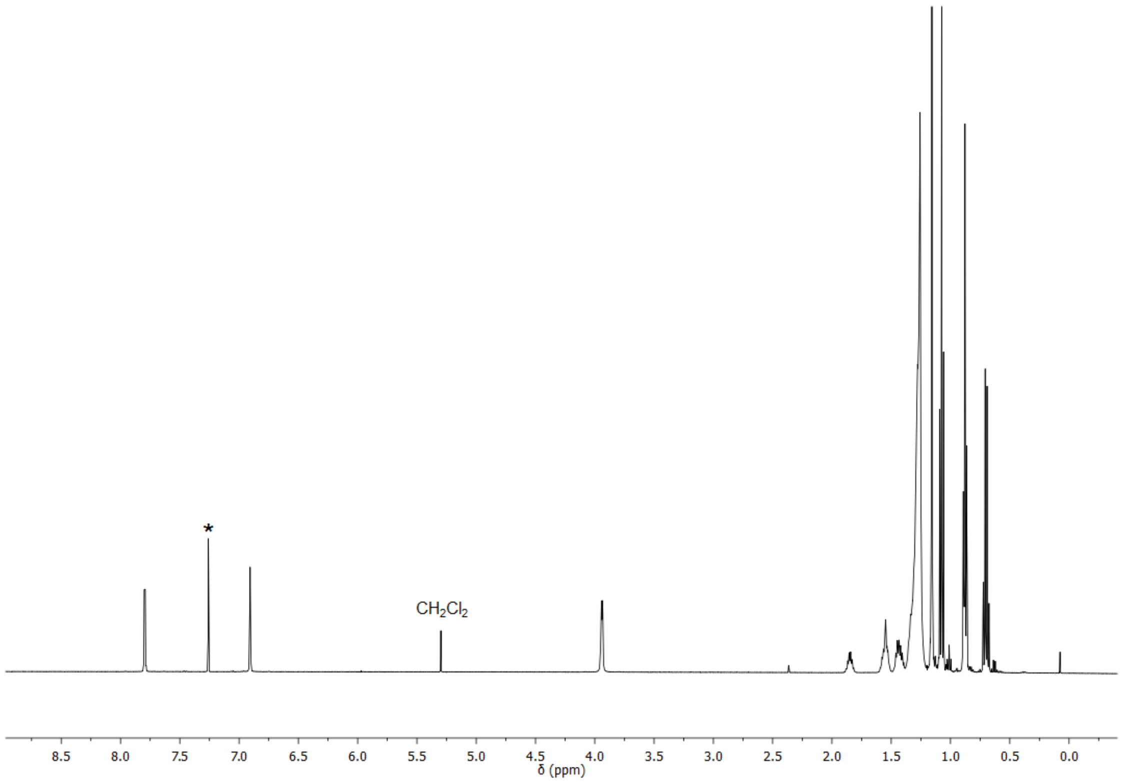

Figure S11: ^{1}H NMR (500 MHz, $CDCl_3$) of **17** (* = NMR solvent residual peak).

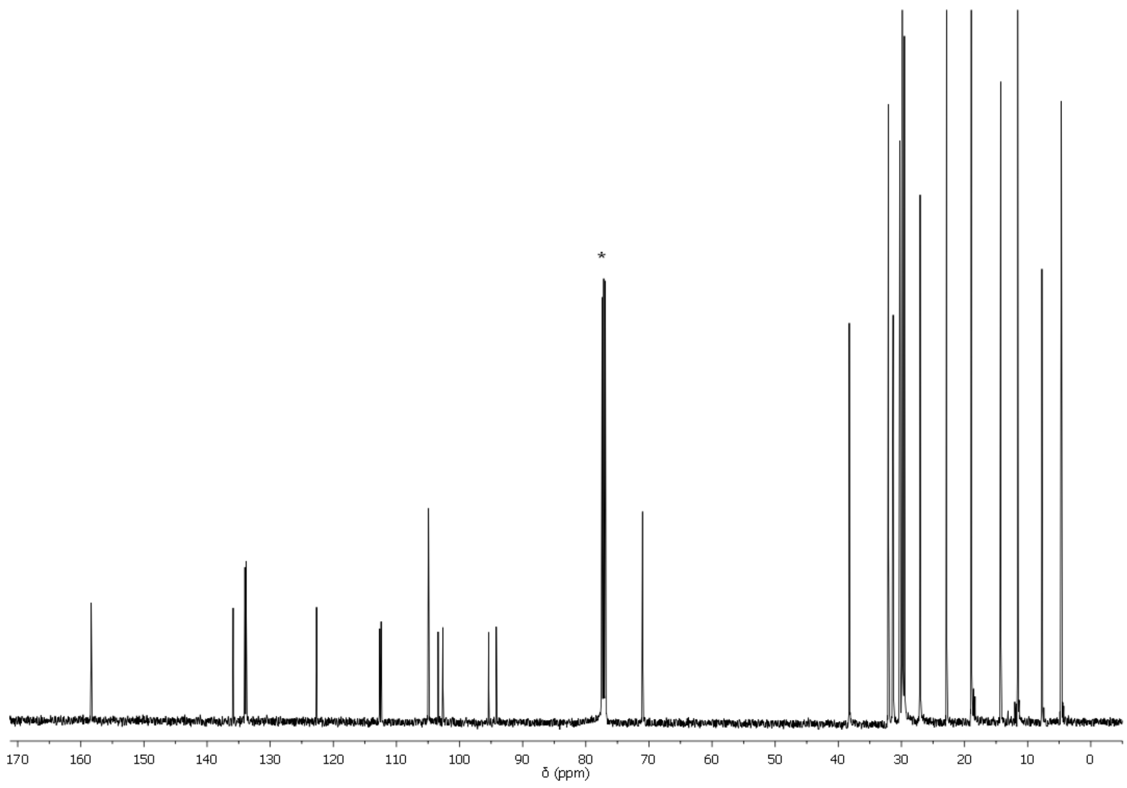

Figure S12: ^{13}C NMR (125 MHz, $CDCl_3$) of **17** (* = NMR solvent residual peak).

Synthesis of 18. To a solution of **15** (413 mg, 506 µmol) in THF (5 mL) K_2CO_3 (230 mg, 1.77 mmol) and MeOH (5 mL) were added. The mixture was heated to 40 °C and stirred for 18 h. After cooling the reaction mixture to room temperature, the mixture was diluted with CH_2Cl_2 and washed with water (2×), acetic acid (10% v/v), water and brine. After drying over $MgSO_4$, the solvent was evaporated and the crude product was purified by column chromatography (silica gel, petroleum ether : CH_2Cl_2 = 8 : 1, R_f = 0.30) to obtain **18** (337 mg, 480 µmol, 95%) as a colorless oil. ^{1}H NMR (500 MHz, $CDCl_3$): δ [ppm] = 7.82 (s, 1H), 7.80 (s, 1H), 6.94 (s, 1H), 6.91 (s, 1H), 4.10 (t, *J* = 6.6 Hz, 2H), 4.06 (t, *J* = 6.3 Hz, 2H), 3.26 (s, 1H), 1.94–1.81 (m, 4H), 1.58–1.47 (m, 4H), 1.43–1.21 (m, 34H), 1.16 (s, 21H), 0.88 (t, *J* = 7.0 Hz, 6H). ^{13}C NMR (125 MHz, $CDCl_3$): δ [ppm] = 158.47, 157.99, 135.96, 134.18, 133.68, 122.68, 112.95, 111.18, 105.44, 105.07, 103.16, 94.69, 80.69, 80.28, 68.79, 68.61, 32.07, 29.85, 29.83, 29.80, 29.78, 29.75, 29.73, 29.68, 29.51, 29.47, 29.12, 26.45, 26.14, 22.84, 18.89, 14.27, 11.56.

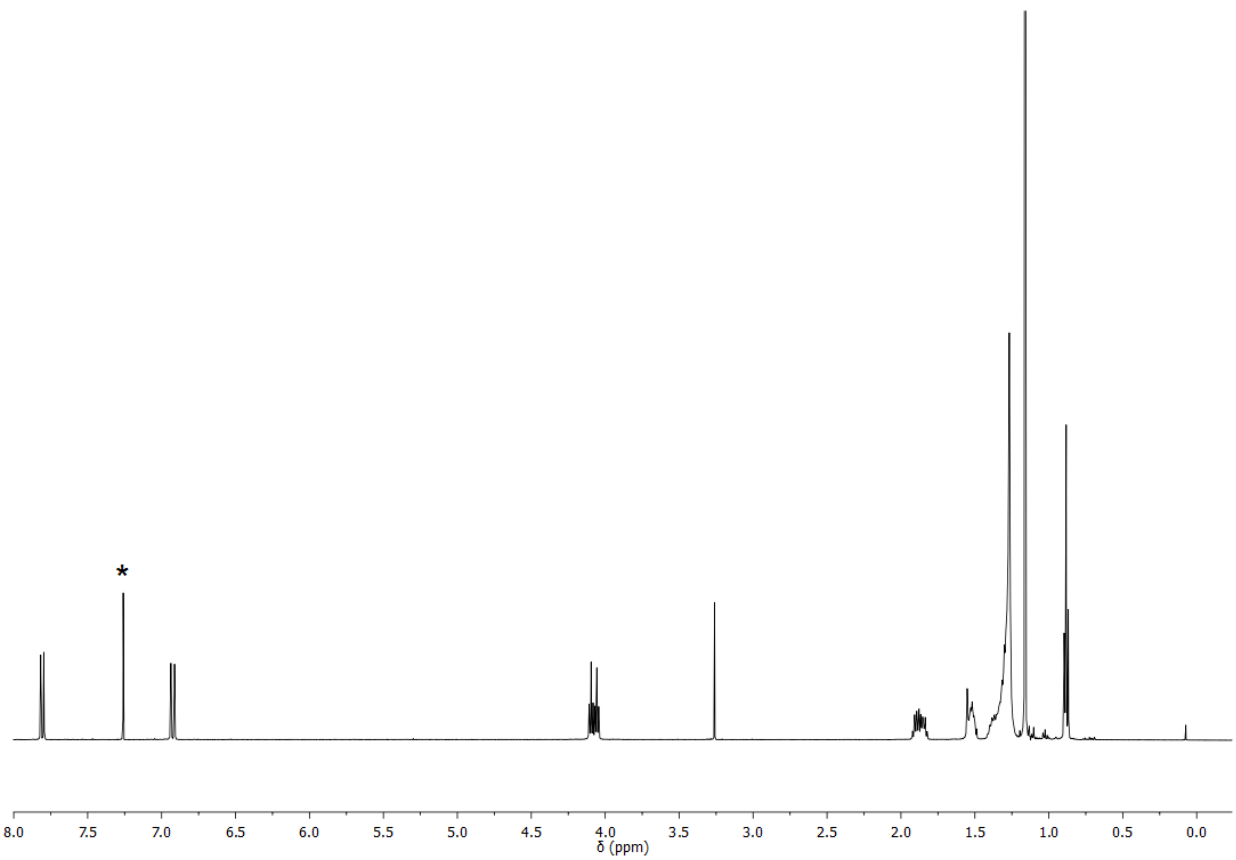

Figure S13: ^{1}H NMR (500 MHz, $CDCl_3$) of **18** (* = NMR solvent residual peak).

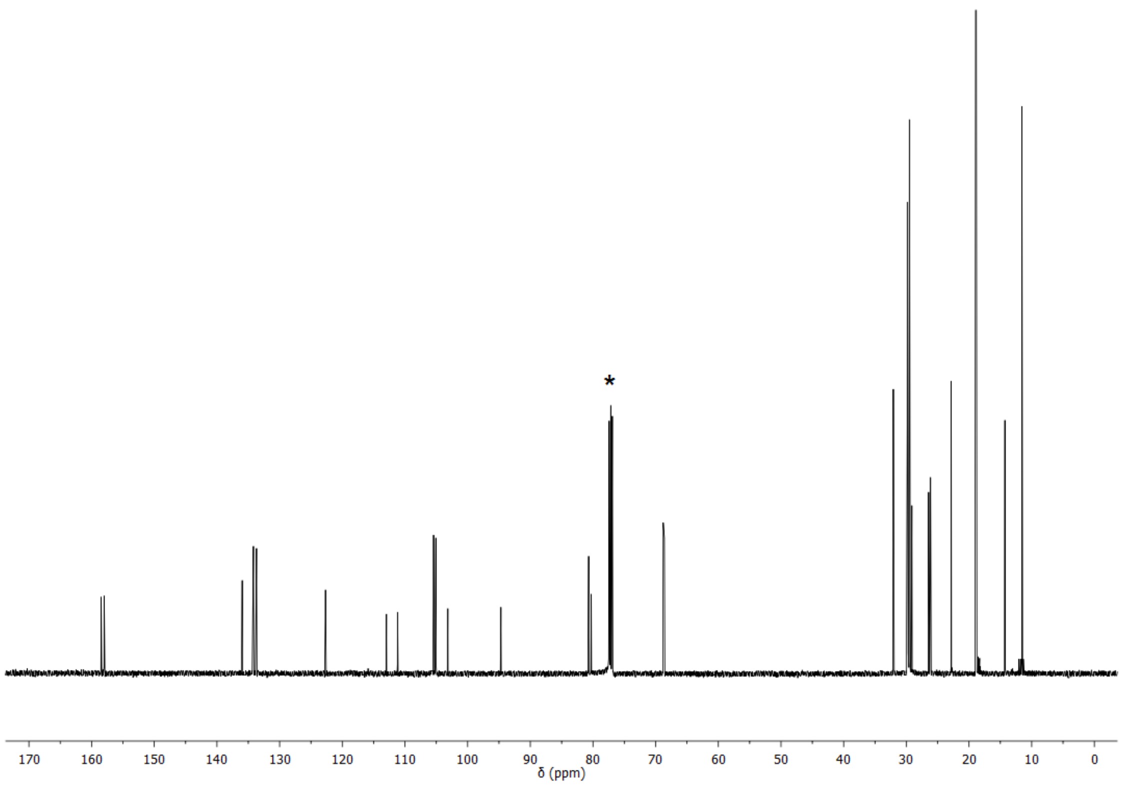

Figure S14: ^{13}C NMR (125 MHz, $CDCl_3$) of **18** (* = NMR solvent residual peak).

Synthesis of 19. To a solution of **16** (442 mg, 477 µmol) in THF (5 mL) K_2CO_3 (212 mg, 1.63 mmol) and MeOH (5 mL) were added. The mixture was heated to 40 °C and stirred for 18 h. After cooling the reaction mixture to room temperature, the mixture was diluted with CH_2Cl_2 and washed with water (2×), acetic acid (10% v/v), water and brine. After drying over $MgSO_4$, the solvent was evaporated and the crude product was purified by column chromatography (silica gel, petroleum ether : CH_2Cl_2 = 8 : 1, R_f = 0.46) to obtain **19** (269 mg, 331 µmol, 69%) as a yellowish viscous oil. ^{1}H NMR (400 MHz, $CDCl_3$): δ [ppm] = 7.82 (s, 1H), 7.80 (s, 1H), 6.94 (s, 1H), 6.91 (s, 1H), 4.09 (t, *J* = 6.7 Hz, 2H), 4.06 (t, *J* = 6.3 Hz, 2H), 3.26 (s, 1H), 1.97–1.77 (m, 4H), 1.63–1.43 (m, 4H), 1.43–1.20 (m, 48H), 1.16 (s, 21H), 0.88 (t, *J* = 6.8 Hz, 6H). ^{13}C NMR (125 MHz, $CDCl_3$): δ [ppm] = 158.48, 158.00, 135.97, 134.18, 133.68, 122.69, 112.97, 111.20, 105.45, 105.08, 103.17, 94.69, 80.69, 80.28, 68.80, 68.62, 32.08, 29.86, 29.83, 29.76, 29.74, 29.68, 29.52, 29.48, 29.13, 26.45, 26.15, 22.85, 18.89, 14.27, 11.57. MS (EI, 70 eV): m/z (%): 812.6 (100) $[M]^{+\cdot}$, 769.5 (36) $[M - C_3H_7]^{+\cdot}$, 545.3 (36) $[M - C_3H_7 - C_{16}H_{32}]^{+\cdot}$; $C_{55}H_{92}O_2Si$ requires 812.69.

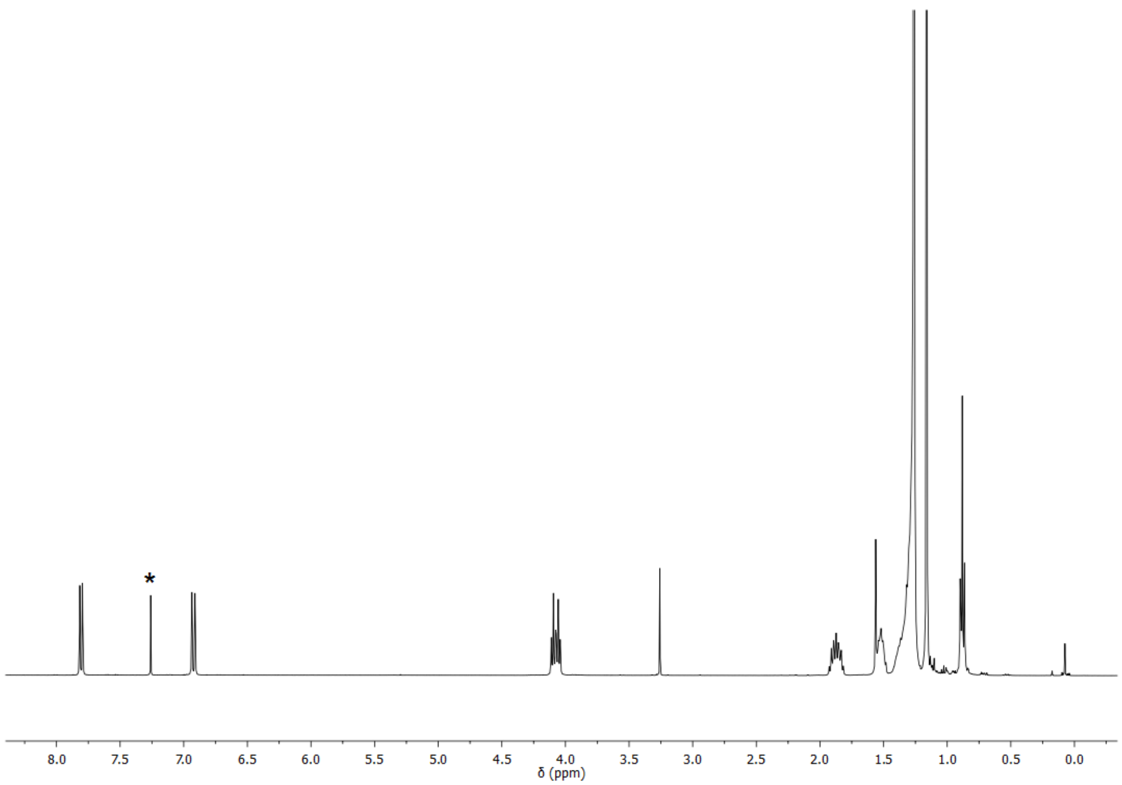

Figure S15: ^{1}H NMR (400 MHz, $CDCl_3$) of **19** (* = NMR solvent residual peak).

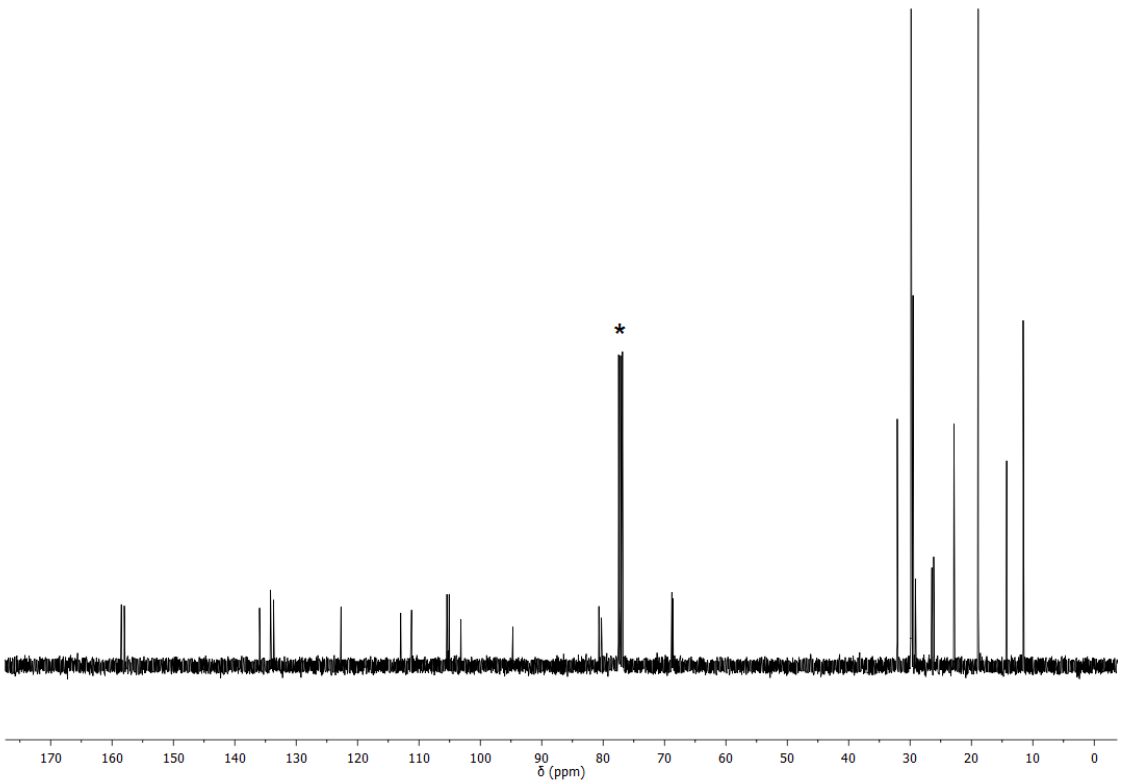

Figure S16: ^{13}C NMR (100 MHz, $CDCl_3$) of **19** (* = NMR solvent residual peak).

Synthesis of 6. To a solution of **17** (535 mg, 515 µmol) in THF (5 mL) K_2CO_3 (258 mg, 1.98 mmol) and MeOH (6 mL) were added. The mixture was heated to 40 °C and stirred for 46 h. After cooling to room temperature, the mixture was diluted with CH_2Cl_2 and washed with water, acetic acid (10% v/v, 2×), Water (3×) and brine. After drying over $MgSO_4$, the solvent was evaporated and the crude product was purified by column chromatography (silica gel, petroleum ether : CH_2Cl_2 = 12 : 1, R_f = 0.45) to obtain **6** (241 mg, 260 µmol, 50%) as a colorless oil. ^{1}H NMR (400 MHz, $CDCl_3$): δ [ppm] = 7.81 (s, 1H), 7.80 (s, 1H), 6.94 (s, 1H), 6.92 (s, 1H), 4.04–3.89 (m, 4H), 3.23 (s, 1H), 1.99–1.78 (m, 2H), 1.65–1.21 (m, 64H), 1.16 (s, 21H), 0.88 (t, J = 6.4 Hz, 12H). ^{13}C NMR (100 MHz, $CDCl_3$): δ [ppm] = 158.52, 158.30, 136.00, 133.98, 122.63, 112.90, 111.32, 105.29, 104.94, 103.26, 94.38, 88.70, 80.62, 80.26, 71.67, 71.08, 38.25, 37.93, 32.08, 32.06, 31.62, 31.30, 30.23, 30.18, 29.87, 29.85, 29.81, 29.79, 29.75, 29.51, 27.02, 22.84, 18.92, 14.27, 11.58. MS (MALDI-TOF, DCTB): m/z (%): 924.8 (100) $[M]^{\cdot+}$; $C_{59}H_{100}O_2Si$ requires 924.81.

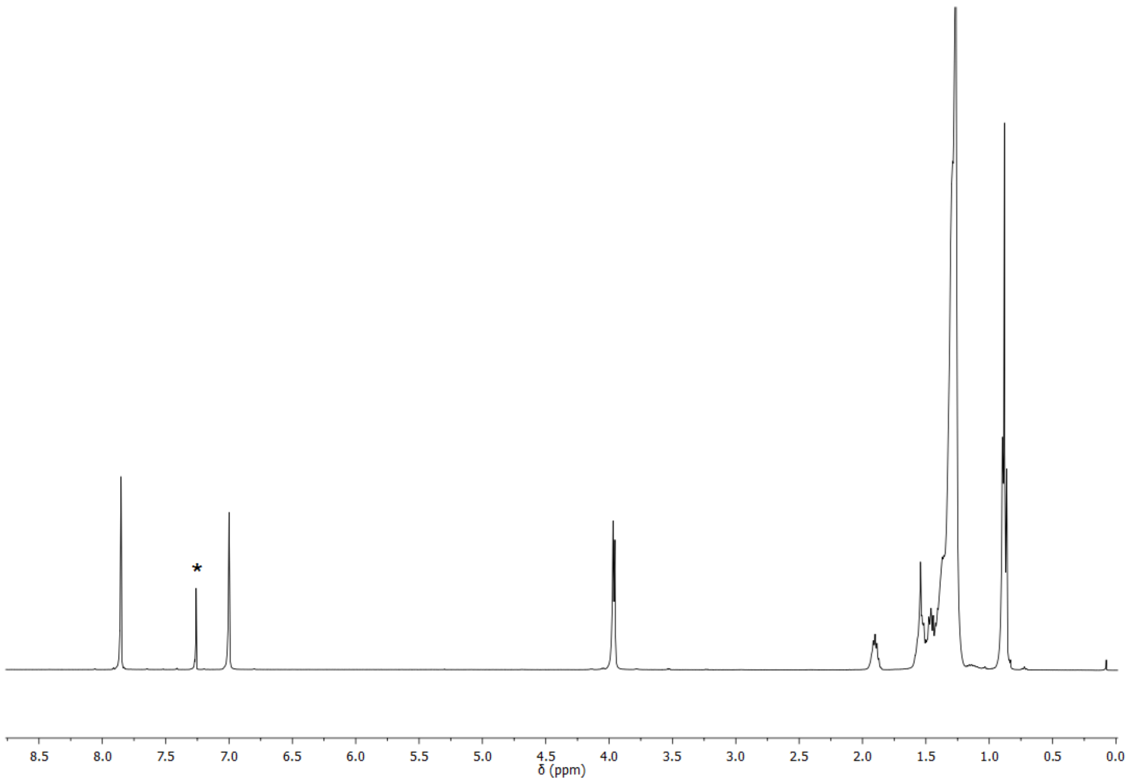

Figure S17: ^{1}H NMR (400 MHz, $CDCl_3$) of **6** (* = NMR solvent residual peak).

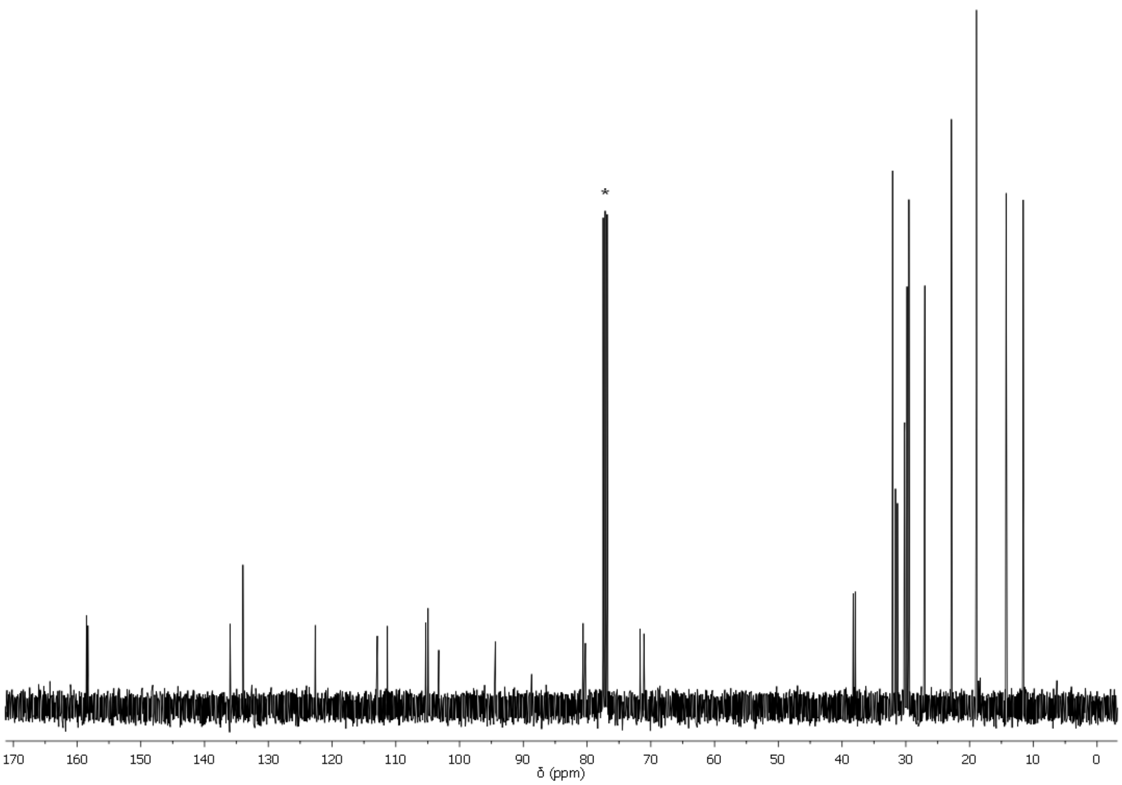

Figure S18: ^{13}C NMR (100 MHz, $CDCl_3$) of **6** (* = NMR solvent residual peak).

Synthesis and characterization of macrocycles **1a**, **3a**, **4a**:

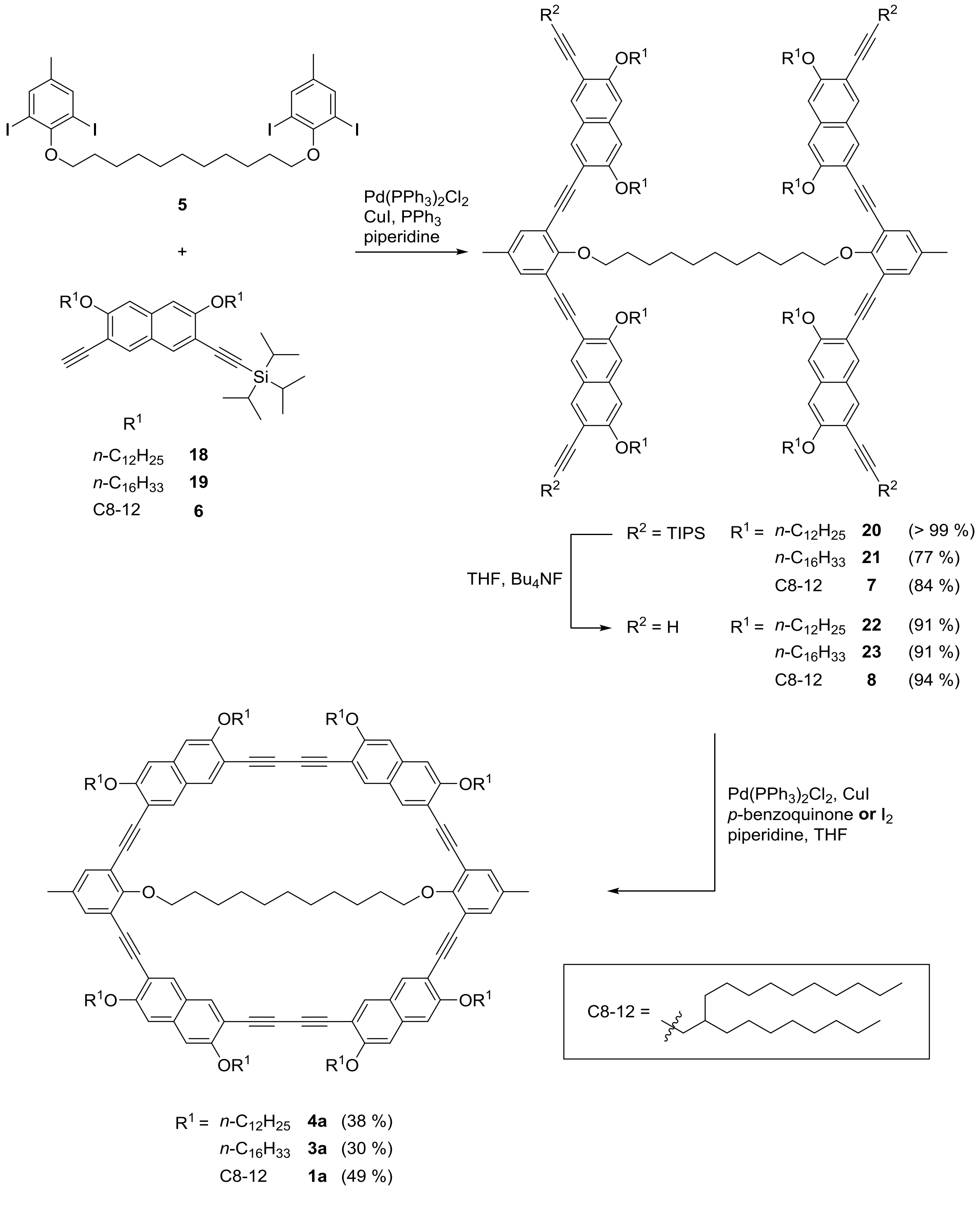

Scheme S2: Reaction pathway towards macrocycles **1a**, **3a**, and **4a**.

Synthesis of the TIPS-protected tetraacetylene 20. 18 (121 mg, 173 µmol), dissolved in dichloromethane, was poured into a Schlenk tube. The solvent was removed under reduced pressure

and an argon atmosphere was applied. **5** (24.5 mg, 28 µmol), $Pd(PPh_3)Cl_2$ (2.8 mg, 4.0 µmol), PPh_3 (3.3 mg, 12.5 µmol) and CuI (0.8 mg, 4.2 µmol) were added and the mixture was dissolved in dry piperidine (1.0 mL). The mixture was stirred for 16 h at 70 °C. After allowing the solution to cool to room temperature, it was diluted with CH_2Cl_2 and water. The organic layer was washed with water (3×), aqueous acetic acid (10%, v/v, 3×), water, aqueous NaOH (10%, w/w), and brine. After drying over $MgSO_4$ the solvent was evaporated. The product was purified by column chromatography (silica gel, petroleum ether : CH_2Cl_2 = 3 : 1, R_f = 0.33) yielding **20** as a colorless oil (94 mg, > 99%, however, still containing few impurities). ^{1}H NMR (500 MHz, $CDCl_3$): δ [ppm] = 7.80 (s, 4H), 7.79 (s, 4H), 7.28 (d, J = 0.6 Hz, 4H), 6.91 (s, 4H), 6.90 (s, 4H), 4.35 (t, J = 6.5 Hz, 4H), 4.12–3.96 (m, 16H), 2.30 (s, 6H), 1.95–1.76 (m, 20H), 1.59–1.41 (m, 20H), 1.40–1.19 (m, 138H), 1.18–1.11 (m, 84H), 0.88 (t, J = 7.0 Hz, 12H), 0.85 (t, J = 7.0 Hz, 12H). ^{13}C NMR (125 MHz, $CDCl_3$): δ [ppm] = 158.97, 158.30, 157.77, 135.76, 134.20, 133.62, 132.93, 132.40, 122.90, 117.94, 112.79, 112.75, 105.35, 105.10, 103.30, 94.46, 90.11, 89.76, 74.67, 68.63, 68.59, 32.09, 32.07, 30.76, 29.88, 29.85, 29.82, 29.80, 29.74, 29.70, 29.53, 29.34, 26.49, 26.32, 22.85, 22.84, 20.55, 18.88, 14.28, 14.27, 11.56. MALDI-MS (DCTB): m/z = 3270.4 (8) $[M + DCTB]^{+\cdot}$, 3162.4 (100) $[M]^{+\cdot}$; $C_{213}H_{332}O_{10}Si_4$ requires 3162.45.

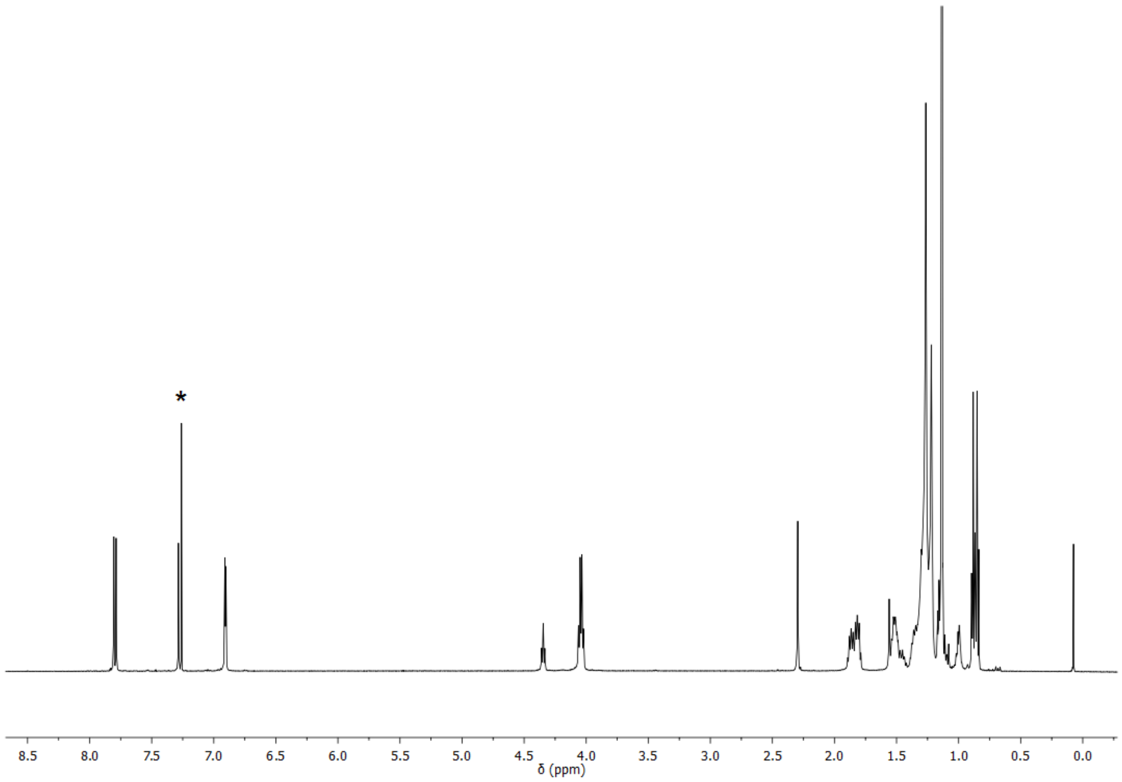

Figure S19: ^{1}H NMR (500 MHz, $CDCl_3$) of **20** (* = NMR solvent residual peak).

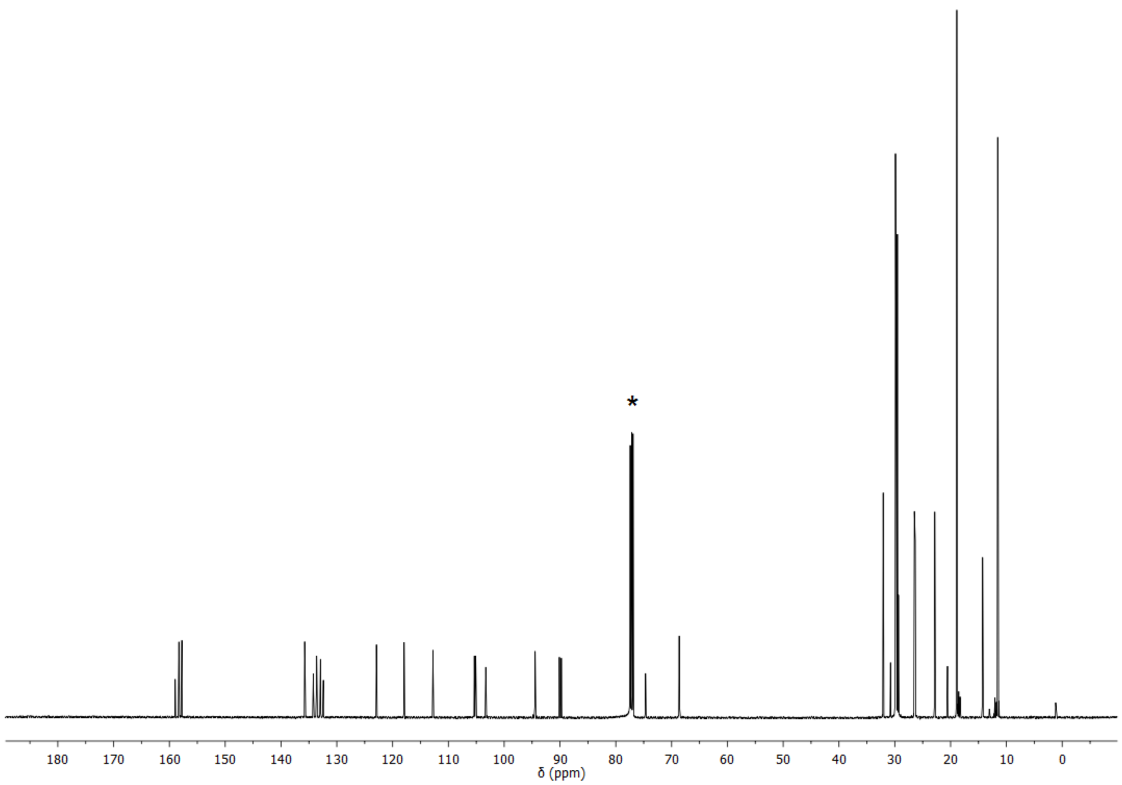

Figure S20: ^{13}C NMR (125 MHz, $CDCl_3$) of **20** (* = NMR solvent residual peak).

Synthesis of the TIPS-protected tetraacetylene 21. 5 (54.7 mg, 62.7 µmol), **19** (246 mg, 302 µmol), $Pd(PPh_3)Cl_2$ (11.0 mg, 15.7 µmol), PPh_3 (11.0 mg, 18.7 µmol) and CuI (1.0 mg, 5.3 µmol) were dissolved in dry piperidine (2.0 mL) and heated to 70 °C. The mixture was stirred for 1.5 h and—after cooling to room temperature—diluted with CH_2Cl_2 and water. The organic layer was washed with water (3×), aqueous acetic acid (10%, v/v, 3×), aqueous NaOH (10%, w/w), water, and brine. After drying over $MgSO_4$ the solvent was evaporated. The product was purified by column chromatography (silica gel, petroleum ether : CH_2Cl_2 = 4 : 1, R_f = 0.34) yielding **21** as a colorless oil (175 mg, 48.4 µmol, 77%). ^1H NMR (500 MHz, $CDCl_3$): δ [ppm] = 7.80 (s, 4H), 7.78 (s, 4H), 7.28 (d, J = 0.7Hz, 4H), 6.91 (s, 4H), 6.90 (s, 4H), 4.34 (t, J = 6.5Hz, 4H), 4.10–3.98 (m, 16H), 2.29 (s, 6H), 1.94–1.74 (m, 20H), 1.61–1.41 (m, 20H), 1.41–1.19 (m, 202H), 1.19–1.07 (m, 84H), 0.93–0.80 (m, 24H). ^{13}C NMR (125 MHz, $CDCl_3$): 158.96, 158.30, 157.76, 135.76, 134.20, 133.62, 132.92, 132.40, 122.89, 117.94, 112.78, 112.74, 105.35, 105.09, 103.30, 94.45, 90.11, 89.76, 74.67, 68.63, 68.59, 32.09, 30.76, 29.88, 29.83, 29.75, 29.71, 29.53, 29.35, 26.50, 26.33, 22.85, 20.56, 18.88, 14.27, 11.55. MALDI-MS (DCTB): m/z = 3865.1 (<5) $[M + DCTB]^{+\cdot}$, 3614.1 (100) $[M]^{+\cdot}$, $C_{245}H_{396}O_{10}Si_4$ requires 3610.96.

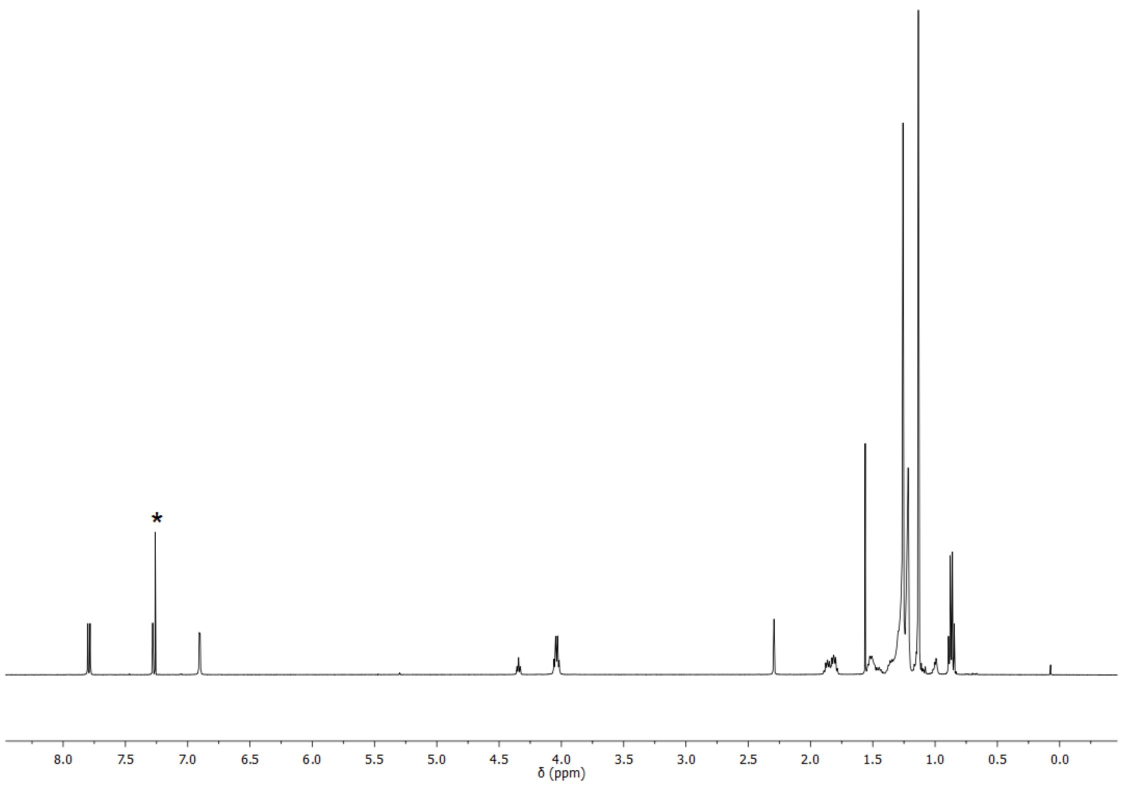

Figure S21: ^{1}H NMR (500 MHz, $CDCl_3$) of **21** (* = NMR solvent residual peak).

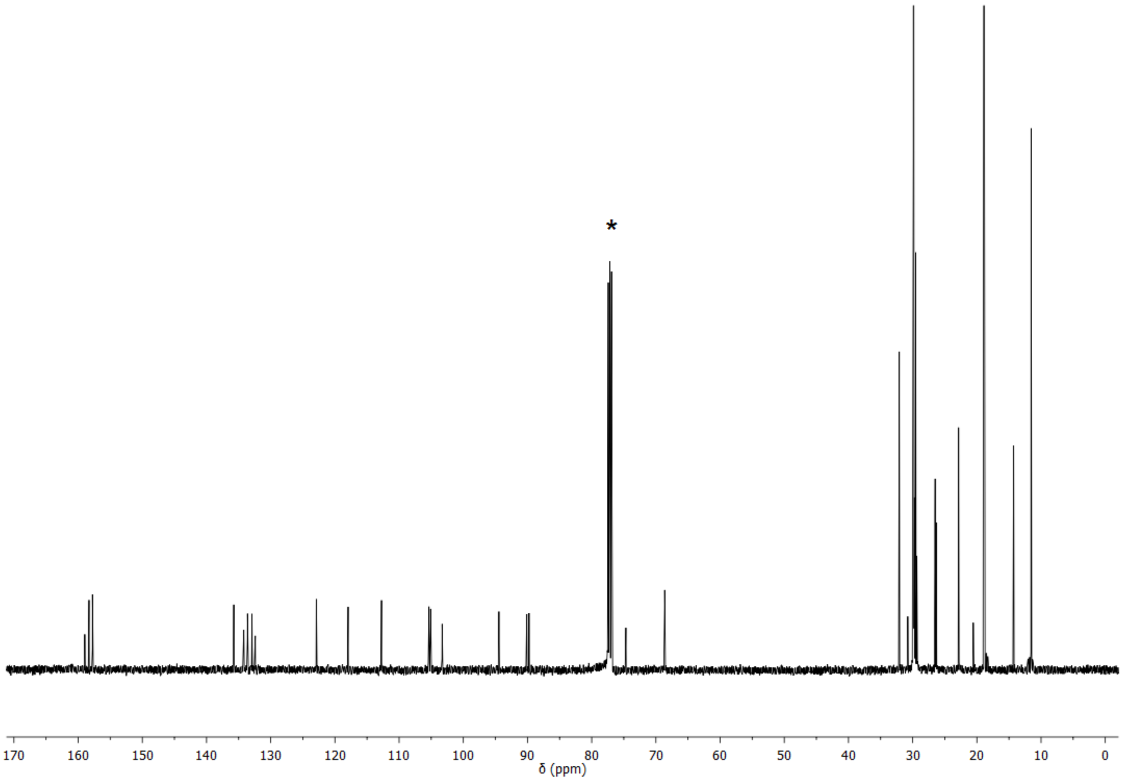

Figure S22: ^{13}C NMR (125 MHz, $CDCl_3$) of **21** (* = NMR solvent residual peak).

Synthesis of the TIPS-protected tetraacetylene 7. 6 (180 mg, 194 µmol), dissolved in dichloromethane, was transferred into a Schlenk tube. The solvent was removed and an argon atmosphere was applied. **5** (35 mg, 40 µmol), $Pd(PPh_3)Cl_2$ (4.0 mg, 5.7 µmol), PPh_3 (4.0 mg, 14.8 µmol) and CuI (1.2 mg, 6.3 µmol) was added and the mixture was dissolved in dry piperidine (1.3 mL). The mixture was stirred for 2.5 h at 70 °C and—after cooling to room temperature—diluted with CH_2Cl_2 and water. The organic layer was washed with water (1×), aqueous acetic acid (10%, v/v, 2×), water (1×), aqueous NaOH (10%, w/w, 1×), and brine. After drying over $MgSO_4$ the solvent was evaporated. The product was purified by column chromatography (silica gel, petroleum ether : CH_2Cl_2 = 6 : 1, R_f = 0.3) yielding **7** as a colorless oil (137 mg, 33.8 µmol, 84%). ^{1}H NMR (500 MHz, $CDCl_3$): δ [ppm] = 7.80 (s, 4H), 7.79 (s, 4H), 7.27 (d, J = 0.6 Hz, 4H), 6.94 (s, 4H), 6.93 (s, 4H), 4.34 (t, J = 6.5 Hz, 4H), 3.96 (d, J = 5.6 Hz, 8H), 3.93 (d, J = 5.2 Hz, 8H), 2.28 (s, 6H), 1.96–1.78 (m, 12H), 1.62–1.17 (m, 270H), 1.17–1.06 (m, 84H), 0.91–0.74 (m, 48H). ^{13}C NMR (125 MHz, $CDCl_3$): 158.89, 158.35, 158.00, 135.77,134.17, 133.92, 132.68, 132.28, 122.85, 118.02, 112.85, 112.76, 105.18, 104.96, 103.36, 94.16, 90.06, 89.80, 74.72, 71.44, 71.06, 38.26, 38.14, 32.08, 32.07, 31.59, 31.27, 30.85, 30.29, 30.24, 30.05, 29.88, 29.86, 29.83, 29.81, 29.55, 29.53, 27.11, 27.03, 26.62, 22.84, 22.83, 20.53, 18.91, 18.57, 18.32, 14.27, 11.56. MALDI-MS (DCTB): m/z = 4063.4 (100) $[M]^{+\cdot}$, $C_{277}H_{460}O_{10}Si_4$ requires 4059.46.

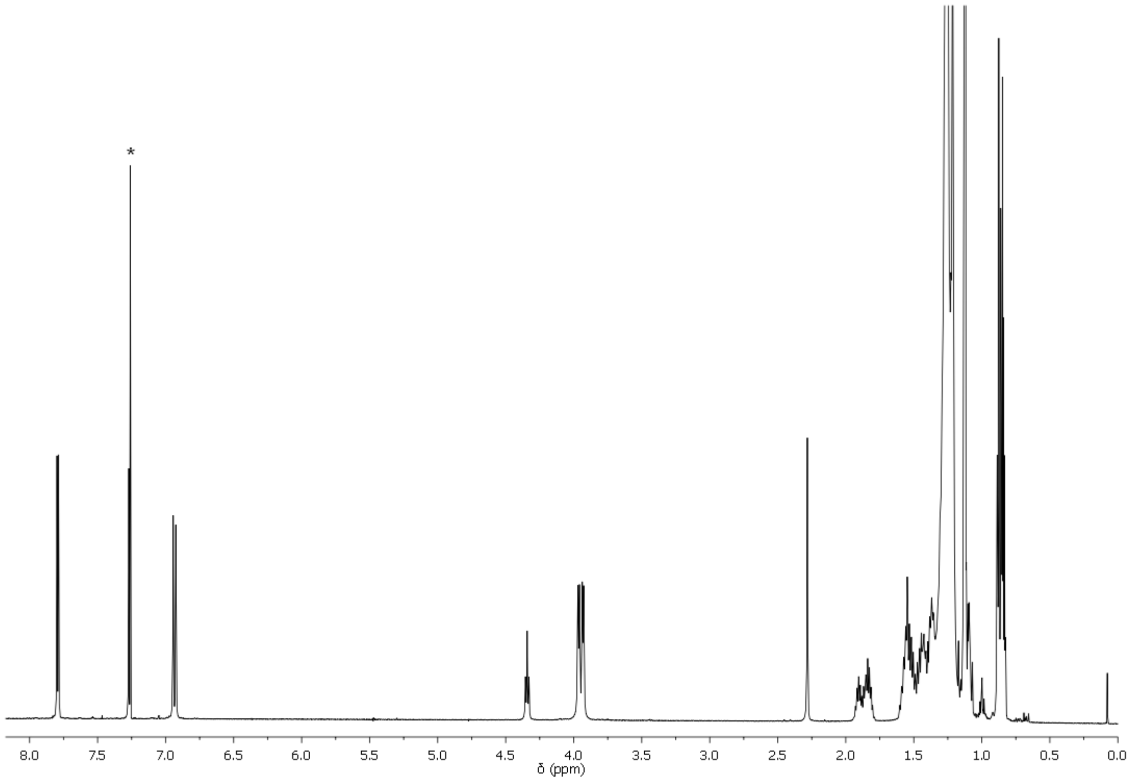

Figure S23: ^{1}H NMR (500 MHz, $CDCl_3$) of **7** (* = NMR solvent residual peak).

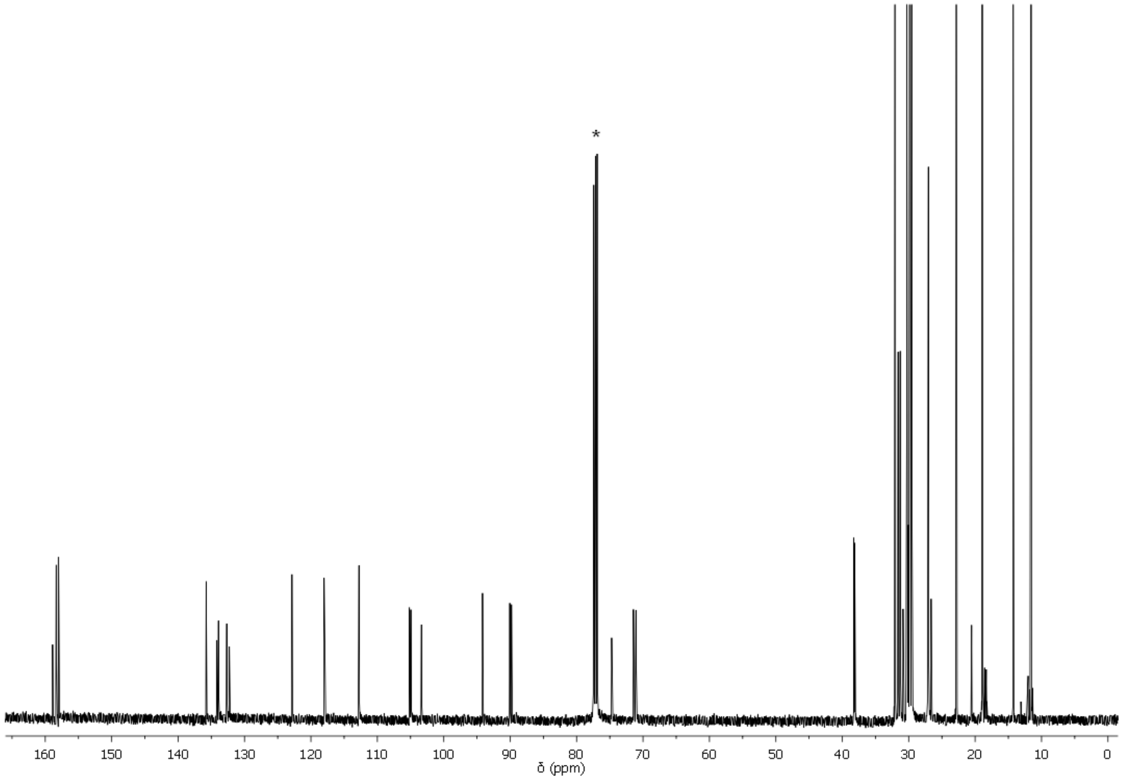

Figure S24: ^{13}C NMR (125 MHz, $CDCl_3$) of **7** (* = NMR solvent residual peak).

Synthesis of the tetraacetylene 22. To **20** (94 mg, 29.7 µmol), dissolved in THF (2 mL), TBAF (1 M in THF, 0.35 mL, 0.35 mmol) was added. The mixture was stirred for 1.5 h at room temperature. The reaction mixture was diluted with CH_2Cl_2 and washed with water (3×) and brine. The organic phase was dried over $MgSO_4$, and the solution was concentrated under reduced pressure. The residue was dissolved in dichloromethane and methanol was added. The precipitate was filtered off through a PTFE membrane yielding **22** (69 mg, 27.2 µmol, 91%) as a red glassy solid. ^{1}H NMR (500 MHz, $CDCl_3$): δ [ppm] = 7.83 (s, 4H), 7.80 (s, 4H), 7.30 (d, J = 0.7 Hz, 4H), 6.94 (s, 4H), 6.94 (s, 4H), 4.35 (t, J = 6.3 Hz, 4H), 4.09 (t, J = 6.6 Hz, 8H), 4.07 (t, J = 6.6 Hz, 8H), 3.26 (s, 4H), 2.30 (s, 6H), 1.96–1.80 (m, 20H), 1.60–1.44 (m, 20H), 1.41–1.15 (m, 138H), 0.90–0.82 (m, 24H). ^{13}C NMR (125 MHz, $CDCl_3$): δ [ppm] = 159.07, 158.00, 157.94, 135.93, 134.20, 133.04, 132.54, 122.87, 117.93, 112.97, 111.35, 105.44, 105.33, 90.00, 89.88, 80.93, 80.20, 77.36, 74.77, 68.80, 68.70, 32.08, 32.07, 30.88, 30.23, 30.17, 29.88, 29.87, 29.85, 29.82, 29.78, 29.75, 29.70, 29.55, 29.52, 29.33, 29.12, 26.72, 26.31, 26.14, 22.85, 22.84, 20.57, 14.27. MALDI-MS (DCTB): m/z (%) = 2788.1 (12) $[M + DCTB]^{+\cdot}$, 2537.9 (100) $[M]^{+\cdot}$, 2368.7 (11) $[M - C_{12}H_{25}]^{+\cdot}$; $C_{177}H_{252}O_{10}$ requires 2537.92.

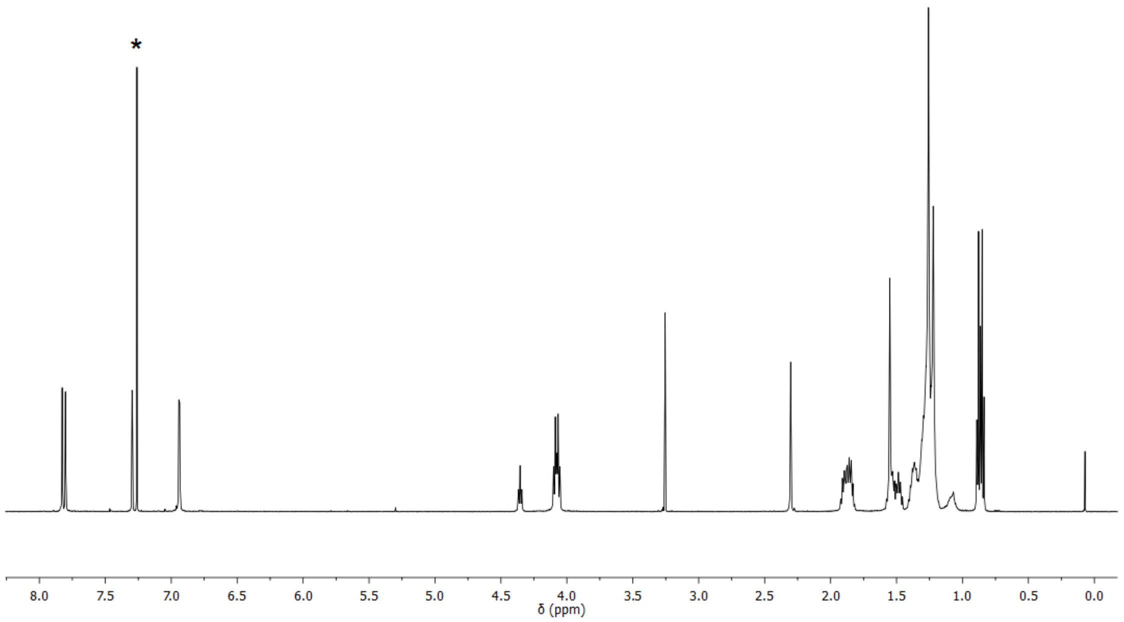

Figure S25: ^{1}H NMR (400 MHz, $CDCl_3$) of **22** (* = NMR solvent residual peak).

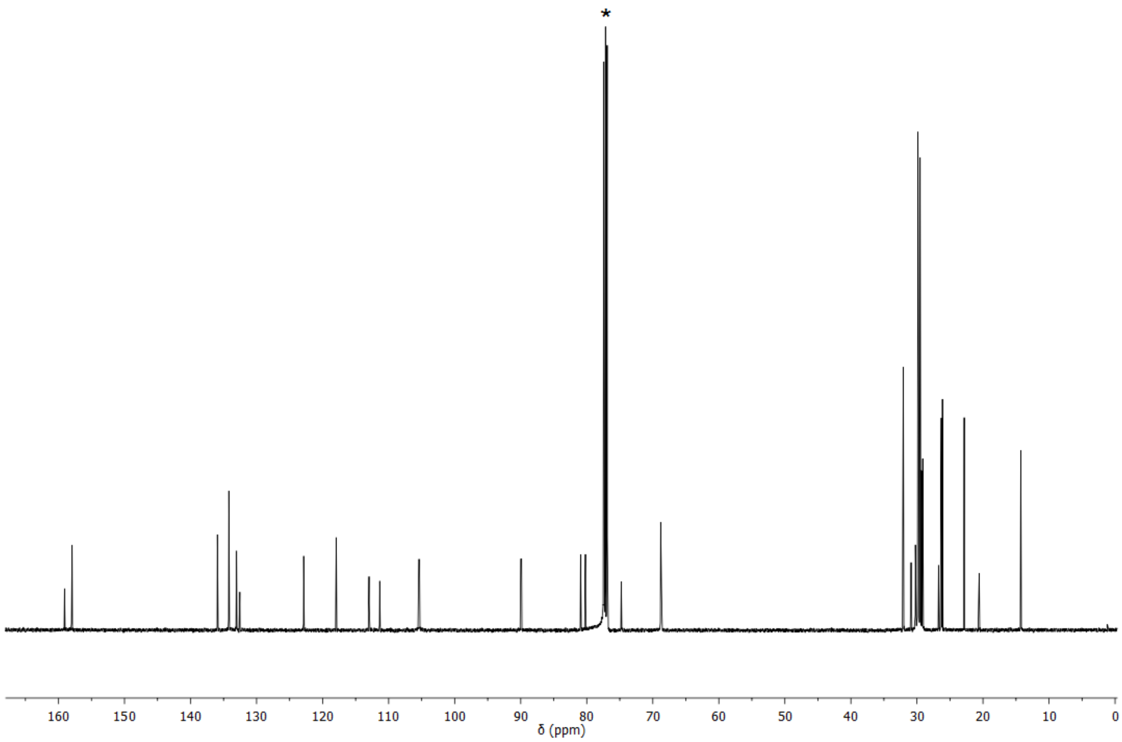

Figure S26: ^{13}C NMR (100 MHz, $CDCl_3$) of **22** (* = NMR solvent residual peak).

Synthesis of the tetraacetylene 23. To **21** (140 mg, 38.7 µmol), dissolved in THF (2 mL), TBAF (1 M in THF, 0.4 mL, 0.04 mmol) was added. The mixture was stirred for 18 h at room temperature. The reaction mixture was diluted with CH_2Cl_2 and washed with water (3×) and brine. The organic phase was dried over $MgSO_4$ and the solution was concentrated under reduced pressure. The residue was dissolved in dichloromethane and methanol was added. The precipitate was filtered through a PTFE membrane yielding **23** (105 mg, 35.1 µmol, 91%) as a colorless solid. M.p. 75 °C. ^{1}H NMR (500 MHz, $CDCl_3$): δ [ppm] = 7.83 (s, 4H), 7.80 (s, 4H), 7.30 (s, 4H), 6.96–6.92 (m, 8H), 4.36 (t, J = 6.3 Hz, 4H), 4.09 (t, J = 6.4 Hz, 8H), 4.07 (t, J = 6.6 Hz, 8H), 3.26 (s, 4H), 2.30 (s, 6H), 1.99–1.78 (m, 20H), 1.62–1.44 (m, 20H), 1.44–1.16 (m, 202H), 0.93–0.80 (m, 24H). ^{13}C NMR (125 MHz, $CDCl_3$): δ [ppm] = 159.07, 158.00, 157.93, 135.93, 134.19, 133.03, 132.53, 122.87, 117.94, 112.98, 111.36, 105.44, 105.34, 90.01, 89.88, 80.93, 80.20, 74.77, 68.80, 68.70, 32.08, 30.88, 30.23, 30.17, 29.87, 29.82, 29.79, 29.76, 29.71, 29.56, 29.53, 29.33, 29.13, 26.72, 26.32, 26.15, 22.84, 20.57, 14.27. MALDI-MS (DCTB): m/z (%) = 3239.5 (<5) $[M + DCTB]^{+\cdot}$, 2988.7 (100) $[M]^{+\cdot}$; $C_{209}H_{316}O_{10}$ requires 2986.42.

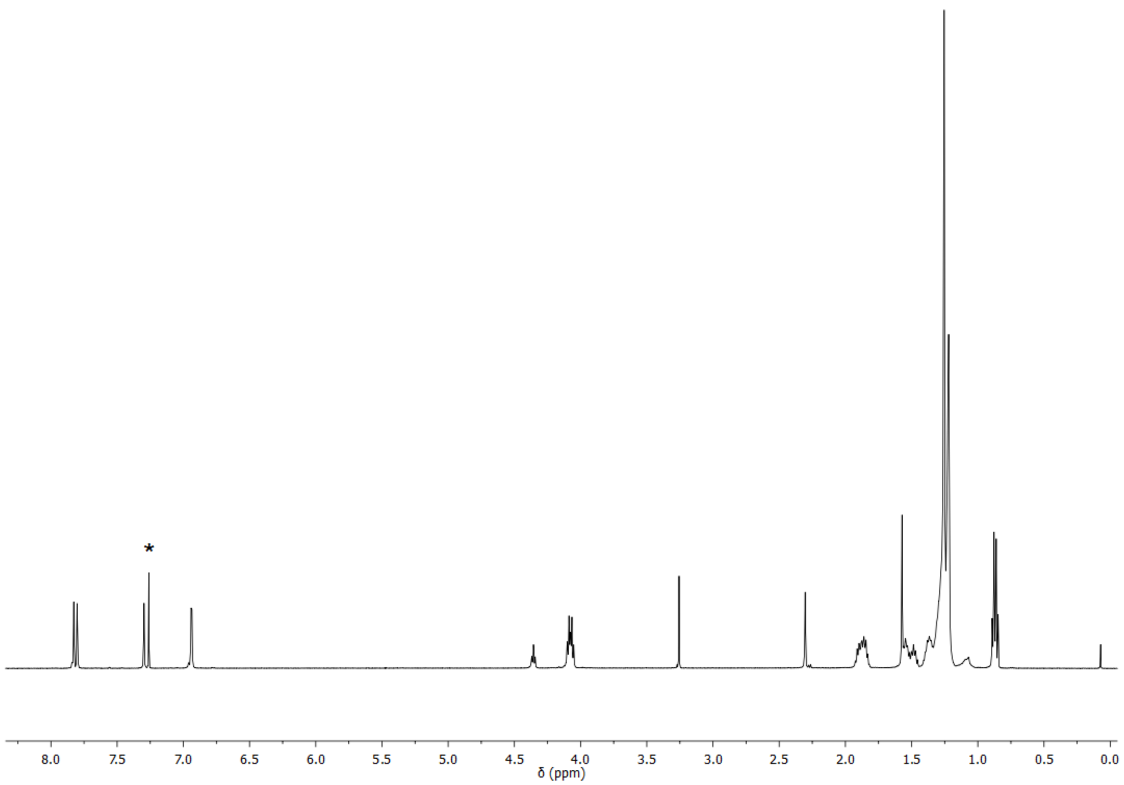

Figure S27: ^{1}H NMR (400 MHz, $CDCl_3$) of **23** (* = NMR solvent residual peak).

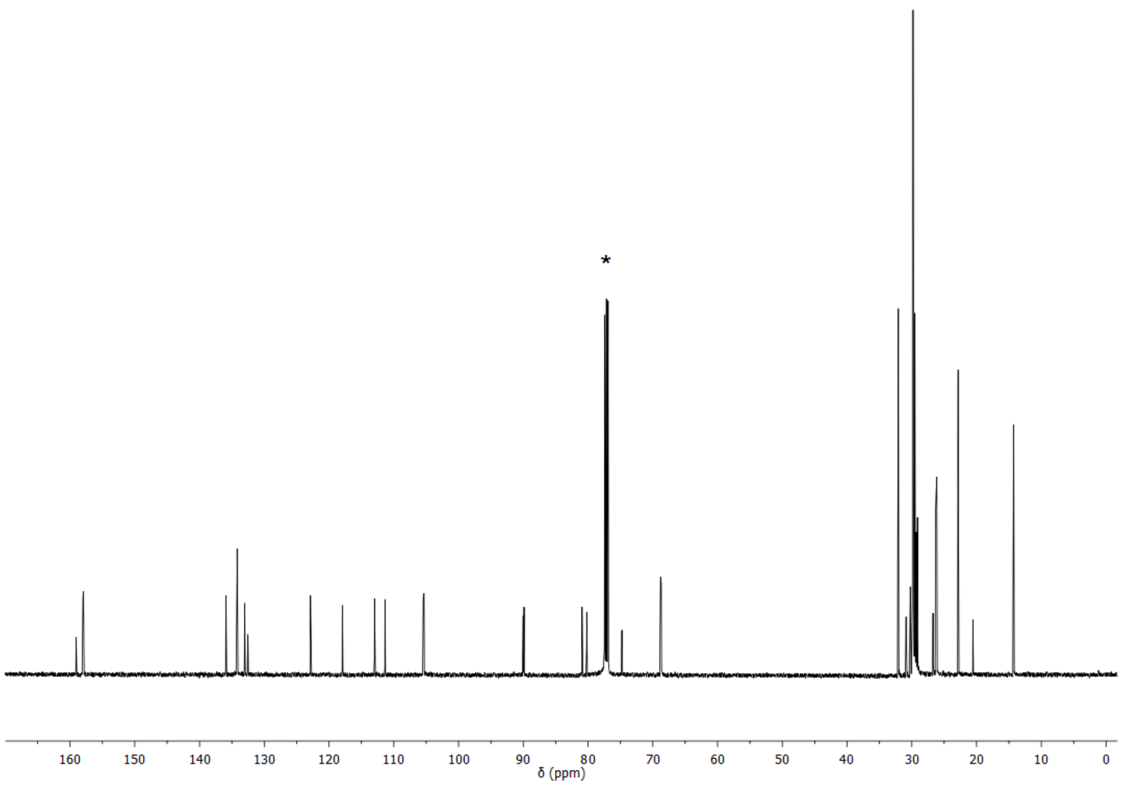

Figure S28: ^{13}C NMR (100 MHz, $CDCl_3$) of **23** (* = NMR solvent residual peak).

Synthesis of the tetraacetylene 8. To **7** (135.5 mg, 33.3 µmol), dissolved in THF (2 mL), TBAF (1 M in THF, 0.4 mL) was added. The mixture was stirred for 1.5 h at room temperature. The reaction mixture was diluted with CH_2Cl_2 and washed with water (3×) and brine. The organic phase was dried over $MgSO_4$, and the solvent was evaporated. The residue was purified by column chromatography (silica gel, PE : CH_2Cl_2 = 3 : 1, R_f = 0.26) yielding **8** (108 mg, 31.4 µmol, 94%) as a colorless oil, which crystallizes after some time. M.p. 43°C. ^{1}H NMR (400 MHz, $CDCl_3$): δ [ppm] = 7.83 (s, 4H), 7.80 (s, 4H), 7.28 (s, 4H), 6.96 (s, 4H), 6.95 (s, 4H), 4.34 (t, J = 6.2 Hz, 4H), 3.98 (d, J = 5.5 Hz, 8H), 3.94 (d, J = 5.7 Hz, 8H), 3.22 (s, 4H), 2.29 (s, 6H), 1.98–1.79 (m, 12H), 1.66–1.05 (m, 270H), 0.94–0.75 (m, 48H). ^{13}C NMR (100 MHz, $CDCl_3$): δ [ppm] = 159.08, 158.31, 158.16, 135.96, 134.06, 132.89, 132.42, 122.85, 118.05, 113.06, 111.51, 105.34, 105.21, 90.00, 89.87, 80.87, 80.19, 74.81, 71.69, 71.53, 38.14, 37.90, 32.08, 32.07, 31.60, 31.58, 30.95, 30.34, 30.30, 30.19, 29.86, 29.81, 29.76, 29.52, 27.12, 27.01, 26.82, 22.84, 22.83, 20.55, 14.27, 14.26. MALDI-MS (DCTB): m/z (%) = 3686.9 (8) [M + DCTB]$^{\cdot+}$, 3437.0 (100) [M]$^{\cdot+}$, $C_{241}H_{380}O_{10}$ requires 3434.92.

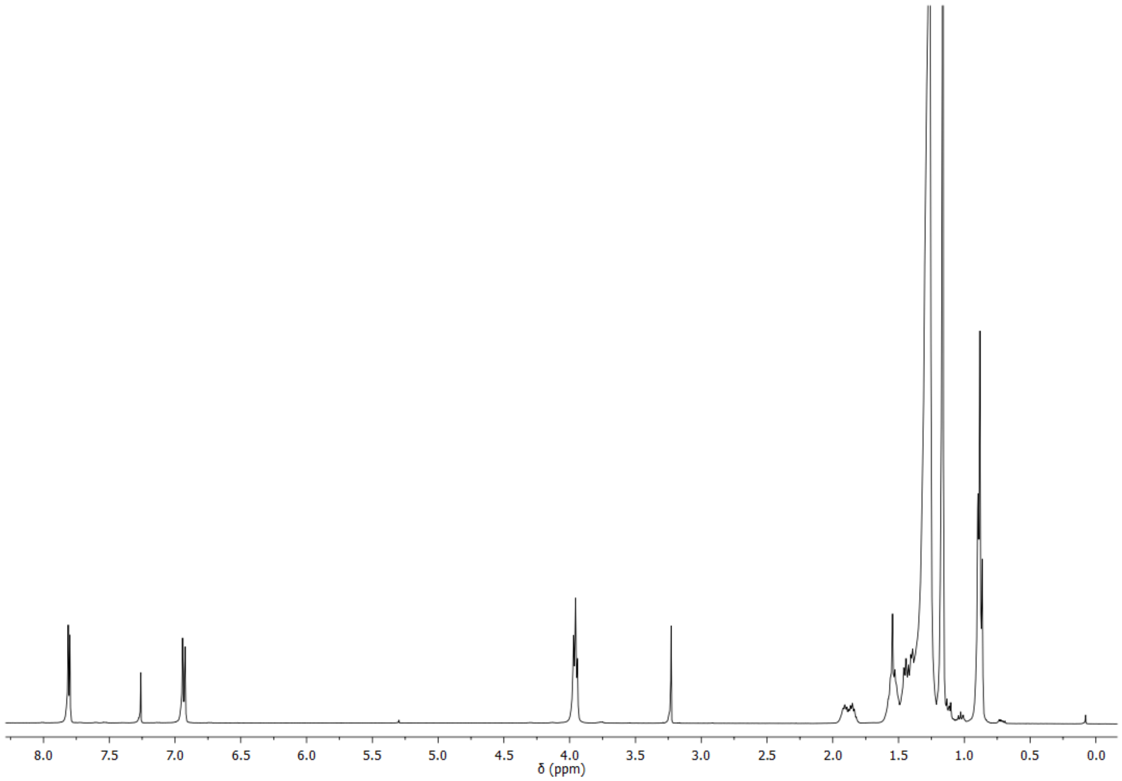

Figure S29: ^{1}H NMR (400 MHz, $CDCl_3$) of **8** (* = NMR solvent residual peak).

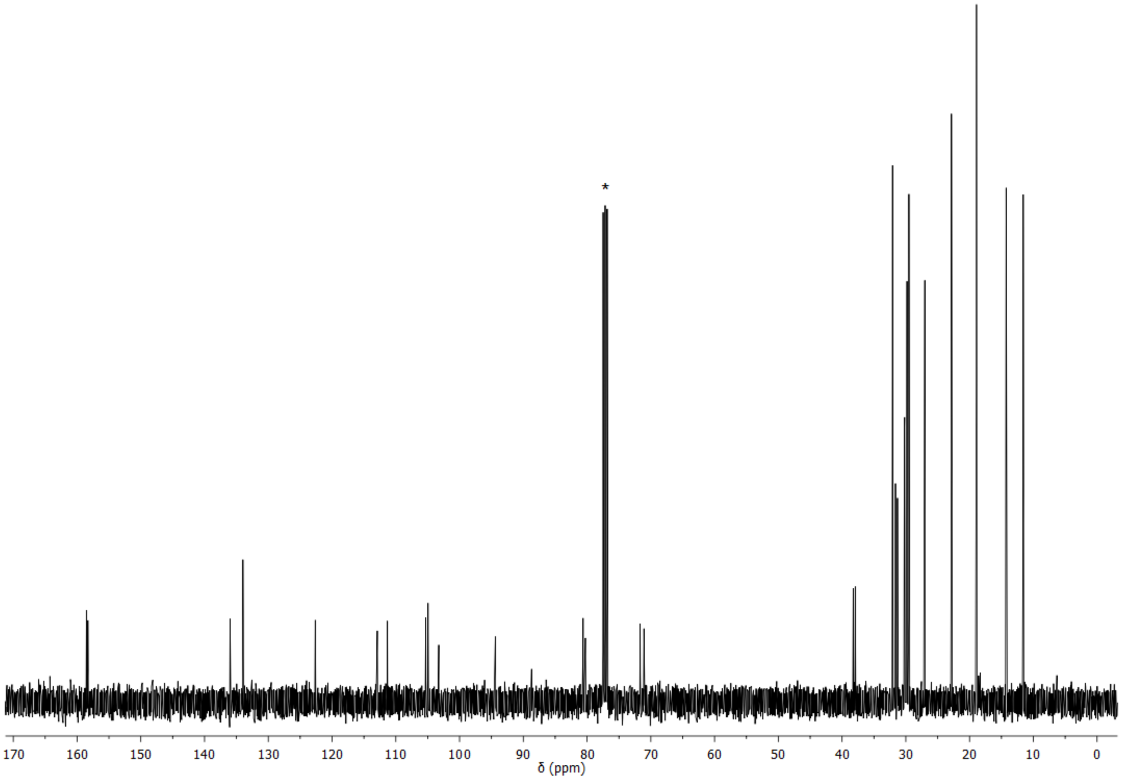

Figure S30: ^{13}C NMR (100 MHz, $CDCl_3$) of **8** (* = NMR solvent residual peak).

Synthesis of macrocycle 4a. Under an argon atmosphere, a 50 ml Hamilton syringe was charged with a solution of tetraacetylene **22** (30.0 mg, 11.8 µmol) in THF (15 mL). $Pd(PPh_3)_2Cl_2$ (4.0 mg, 5.7 µmol, 0.5 equiv) and CuI (3.0 mg, 15.7 µmol, 1.5 equiv) together with iodine (8.0 mg, 42.0 µmol, 3.6 equiv) were dissolved in THF (15 mL) and diisopropylamine (15 mL) and heated to 50 °C. While stirring vigorously, the acetylene solution was added dropwise to the catalyst/oxidant solution over a period of 48 h. The mixture was stirred for additional 20 h at 50 °C. After letting the reaction mixture cool to room temperature, it was diluted with chloroform and water. The aqueous phase was extracted with chloroform once and the collected organic phases were washed with water (3×), acetic acid (10% v/v, 3×), water, NaOH (10% w/w), and brine, and subsequently dried over $MgSO_4$. The solvent was removed under reduced pressure. In a first cleaning step, the residue was separated from the inorganic impurities by column chromatography (silica gel, petroleum ether : CH_2Cl_2 = 1 : 1, R_f = 0.5). After removing the solvent, the crude product was dissolved in chloroform and methanol was added. The suspension was filtered through a PTFE membrane. The residue was recrystallized from ethyl acetate to yield **4a** as a slightly yellow solid (11.4 mg, 4.54 µmol, 38%). M.p. 201 °C (LC), 227 °C (T_{cl}). ^{1}H NMR (500 MHz, $CDCl_3$, 298 K): δ [ppm] = 7.98 (s, 4H), 7.77 (s, 4H), 7.29 (d, J = 0.6 Hz, 4H), 6.96 (s, 4H), 6.96 (s, 4H), 4.38 (t, J = 7.2 Hz, 4H), 4.18–4.08 (m, 16H), 2.31 (s, 6H), 2.20–2.08 (m, 4H), 2.01–1.89 (m, 16H), 1.77–1.17 (m, 158H), 0.92–0.82 (m, 24H). ^{13}C NMR (126 MHz, CD_2Cl_2, 298 K): δ [ppm] = 158.58, 158.21, 157.93, 136.09, 136.03, 134.36, 132.70, 132.59, 123.13, 117.93, 113.16, 111.39, 105.56, 105.35, 90.10, 89.78, 79.79, 77.97, 74.94, 68.97, 68.68, 32.37, 32.09, 32.07, 32.04, 31.13, 29.92, 29.86, 29.83, 29.81, 29.77, 29.56, 29.52, 29.42, 29.09, 27.08, 26.44, 26.17, 22.85, 20.58, 14.28. MALDI-MS (DCTB): m/z (%) = m/z (%): 3034.2 (5) $[M + 2DCTB]^{+\cdot}$, 2783.9 (21) $[M + DCTB]^{+\cdot}$, 2533.9 (100) $[M]^{+\cdot}$, 2408.0 (15) $[M - C_{12}H_{24}]^{+\cdot}$. $C_{177}H_{248}O_{10}$ requires 2533.89.

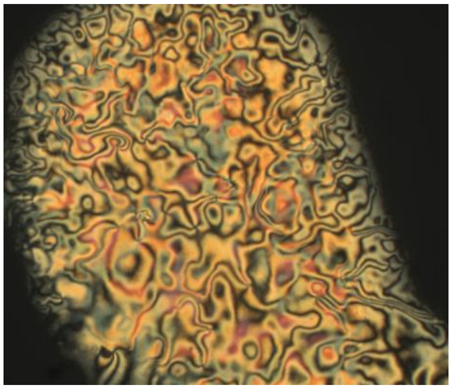

Figure S31: Texture of **4a** at 194 °C after cooling from the isotropic phase.

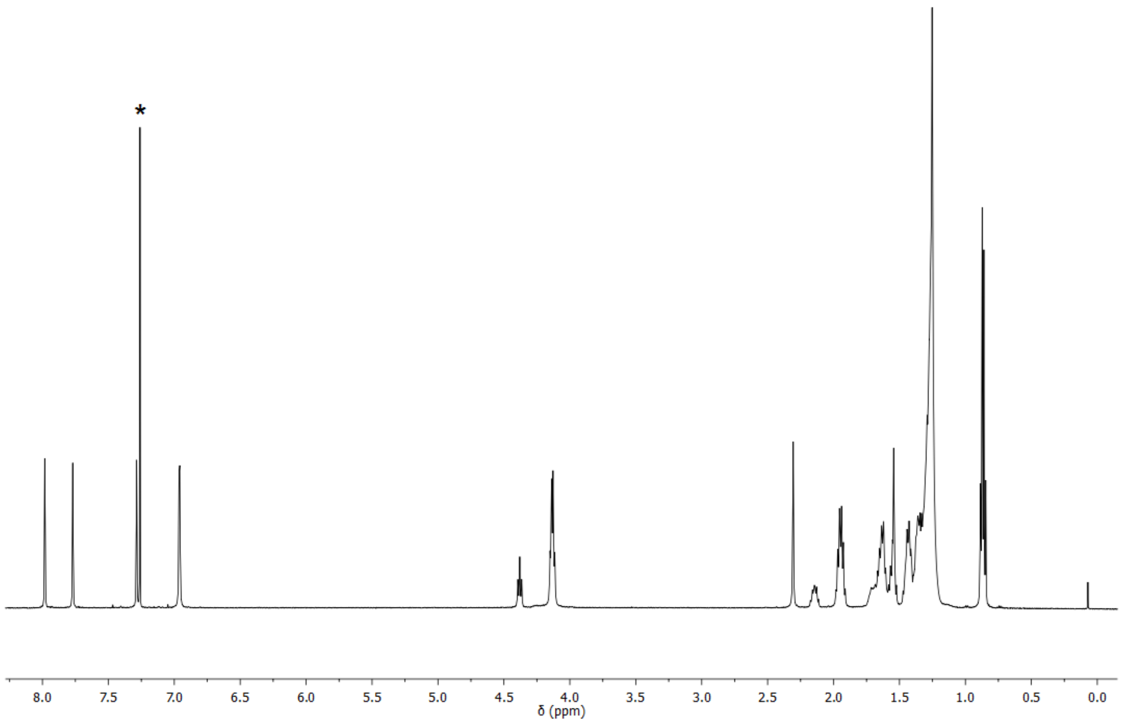

Figure S32: ^{1}H NMR (500 MHz, CD_2Cl_2) of **4a.** (* = NMR solvent residual peak).

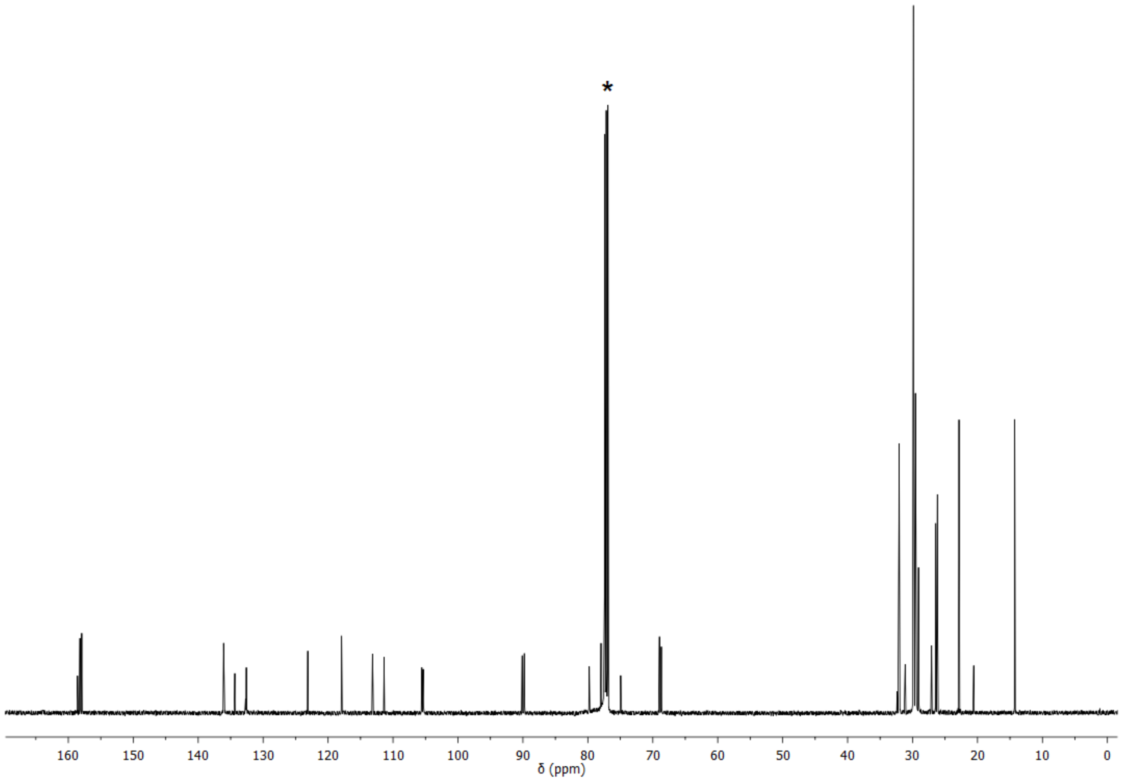

Figure S33: ^{13}C NMR (126 MHz, CD_2Cl_2) of **4a.** (* = NMR solvent residual peak).

Synthesis of macrocycle 3a. Under an argon atmosphere, a 50 mL Hamilton syringe was charged with a solution of tetraacetylene **23** (30.6 mg, 10.2 µmol) in THF (10 mL). $Pd(PPh_3)_2Cl_2$ (2.0 mg, 2.85 µmol, 0.3 equiv) and CuI (2.7 mg, 14.2 µmol, 1.4 equiv) together with 1,4-benzoquinone (8.4 mg, 77.7 µmol, 7.6 equiv) were dissolved in THF (10 mL) and piperidine (10 mL) and heated to 50 °C. While stirring vigorously, the acetylene solution was added dropwise to the catalyst/oxidant solution over a period of 96 h. The mixture was stirred for additional 18 h at 50 °C. After letting the reaction mixture cool to room temperature, it was diluted with chloroform and water. The aqueous phase was additionally extracted once with chloroform and the combined organic phases were washed with water (3×), acetic acid (10% v/v, 3×), water (2×), NaOH (10% w/w), and brine, and subsequently dried over $MgSO_4$. The solvent was removed under reduced pressure. In a first cleaning step, the residue was separated from the inorganic impurities by column chromatography (silica gel, petroleum ether : CH_2Cl_2 = 4 : 3, R_f = 0.8 (1:1)). The resulting crude product was further purified by preparative recycling GPC. The solvent of the monodisperse product fraction was removed under reduced pressure. The solid residue was dissolved in CH_2Cl_2 and methanol was added to precipitate the product from the BHT-containing solution. The suspension was filtered through a PTFE membrane and the residue was precipitated from cold CH_2Cl_2 to yield **1** as a colorless solid (9.0 mg, 3.02 µmol, 30%). M.p. 174 °C. ^{1}H NMR (500 MHz, $CDCl_3$, 298 K): δ [ppm] = 7.99 (s, 4H), 7.78 (s, 4H), 7.29 (d, J = 0.8 Hz, 4H), 6.97 (s, 4H), 6.96 (s, 4H), 4.38 (t, J = 7.1 Hz, 4H), 4.18 – 4.10 (m, 16H), 2.30 (s, 6H), 2.20 – 2.09 (m, 4H), 2.00 – 1.89 (m, 16H), 1.76 – 1.17 (m, 222H), 0.872 (t, J = 7.0 Hz, 12H), 0.868 (t, J = 6.9 Hz, 12H). ^{13}C NMR (126 MHz, $CDCl_3$, 298 K): δ [ppm] = 158.58, 158.21, 157.93, 136.09, 136.03, 134.36, 132.72, 132.60, 123.13, 117.93, 113.17, 111.40, 105.57, 105.36, 90.10, 89.79, 79.79, 77.96, 74.95, 68.98, 68.69, 32.09, 32.05, 31.14, 29.93, 29.90, 29.88, 29.84, 29.76, 29.58, 29.54, 29.43, 29.09, 27.08, 26.45, 26.18, 22.86, 20.59, 14.29. MALDI-MS (DCTB): m/z (%) = 2984.9 (100) $[M]^{+\cdot}$. $C_{209}H_{312}O_{10}$ requires 2982.39. GPC (PS calibration): single peak with $M_w = 4.31 \times 10^3$ g mol^{-1}.

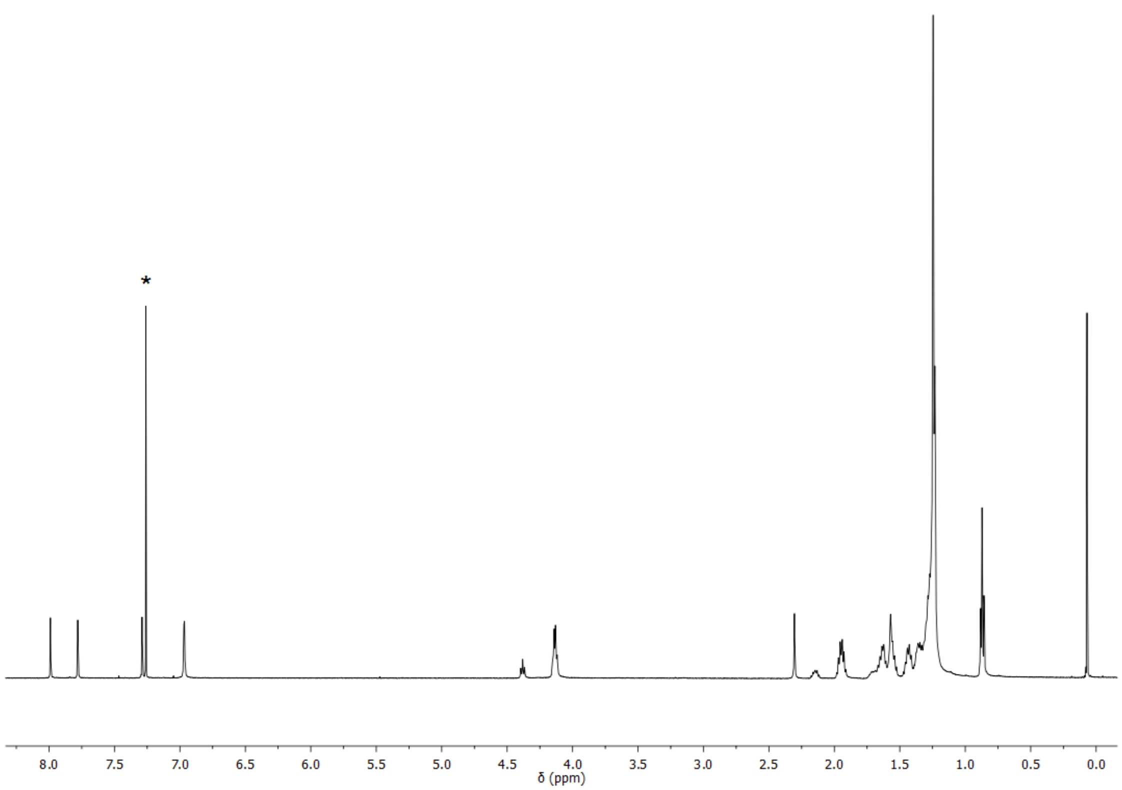

Figure S34: ^{1}H NMR (500 MHz, $CDCl_3$) of **3a** (* = NMR solvent residual peak).

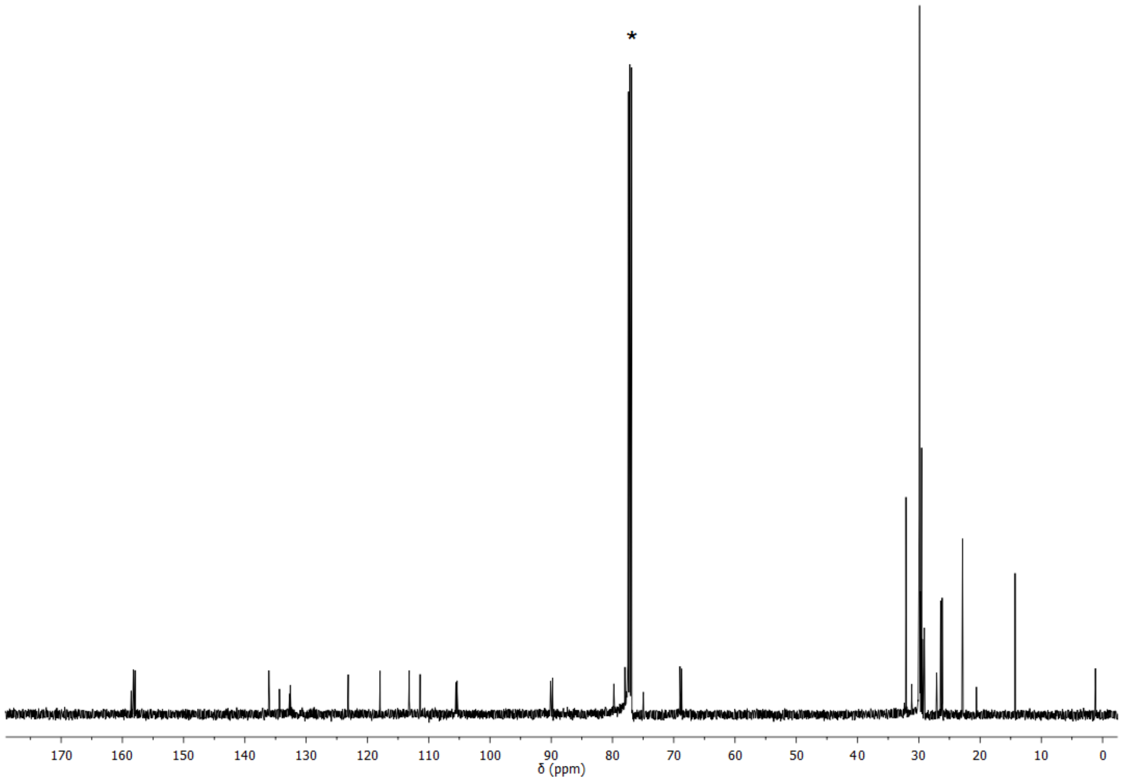

Figure S35: ^{13}C NMR (125 MHz, $CDCl_3$) of **3a** (* = NMR solvent residual peak).

Synthesis of macrocycle 1a. Under an argon atmosphere, a 50 mL Hamilton syringe was charged with a solution of tetraacetylene **8** (31.4 mg, 9.135 µmol) in THF (10 mL). $Pd(PPh_3)_2Cl_2$ (3.05 mg, 4.99 µmol, 0.5 equiv) and CuI (1.5 mg, 7.88 µmol, 0.8 equiv) together with 1,4-benzoquinone (9.0 mg, 83.3 µmol, 9.1 equiv) were dissolved in THF (10 mL) and piperidine (10 mL) and heated to 50 °C. While stirring vigorously, the acetylene solution was added dropwise to the catalyst/oxidant solution over a period of 48 h. The mixture was stirred for additional 43 h at 50 °C. After letting the reaction mixture cool to room temperature, it was diluted with CH_2Cl_2 and water. The organic layer was washed with water (3×), acetic acid (10% v/v, 3×), water, NaOH (10% w/w), and brine, and subsequently dried over $MgSO_4$. The solvent was removed under reduced pressure. In a first cleaning step, the residue was separated from the inorganic impurities by column chromatography (silica gel, petroleum ether : CH_2Cl_2 = 4 : 1, R_f = 0.3). The resulting crude product was further purified by preparative recycling GPC. The solvent of the monodisperse product fraction was removed under reduced pressure. The residue was dissolved in CH_2Cl_2 and, by adding methanol, the product was precipitated from the BHT containing solution. The suspension was filtered through a PTFE membrane to yield **1** as a slightly yellow solid (15.4 mg, 4.49 µmol, 49%). M.p. 63 °C (LC), 142 °C (T_{cl}). ^{1}H NMR (500 MHz, CD_2Cl_2, 298 K): δ [ppm] = 8.00 (s, 4H), 7.82 (s, 4H), 7.31 (d, J = 0.5 Hz, 4H), 7.04 (s, 4H), 7.03 (s, 4H), 4.38 (t, J = 7.3 Hz, 4H), 4.14–3.98 (m, 16H), 2.32 (s, 6H), 2.24–2.11 (m, 4H), 2.03–1.88 (m, 8H), 1.77–1.07 (m, 270H), 0.86 (t, J = 6.9 Hz, 24H), 0.84 (t, J = 7.0 Hz, 24H). ^{13}C NMR (126 MHz, CD_2Cl_2, 298 K): δ [ppm] = 158.98, 158.84, 158.78, 136.74, 136.03, 134.65, 133.52, 132.98, 123.37, 118.38, 113.37, 111.81, 105.87, 105.67, 90.29, 90.21, 79.94, 78.08, 75.45, 71.91, 38.67, 38.34, 32.93, 32.63, 32.58, 32.55, 32.51, 32.49, 32.48, 32.05, 32.02, 31.72, 30.71, 30.38, 30.35, 30.31, 30.29, 30.26, 30.25, 30.22, 30.22, 30.02, 29.99, 29.93, 27.54, 27.52, 27.45, 27.43, 23.28, 23.26, 23.24, 20.77, 14.45. MALDI-MS (DCTB): m/z (%) = 6862.0 (3) $[2M]^{\cdot+}$, 4173.3 (10) $[M+3DCTB]^{\cdot+}$, 3932.6 (55) $[M+2DCTB]^{\cdot+}$, 3682.3 (100) $[M+DCTB]^{\cdot+}$, 3432.4 (62) $[M]^{\cdot+}$. $C_{241}H_{376}O_{10}$ requires 3430.89. GPC (PS calibration): single peak with $M_w = 4.66 \times 10^3$ g mol^{-1}.

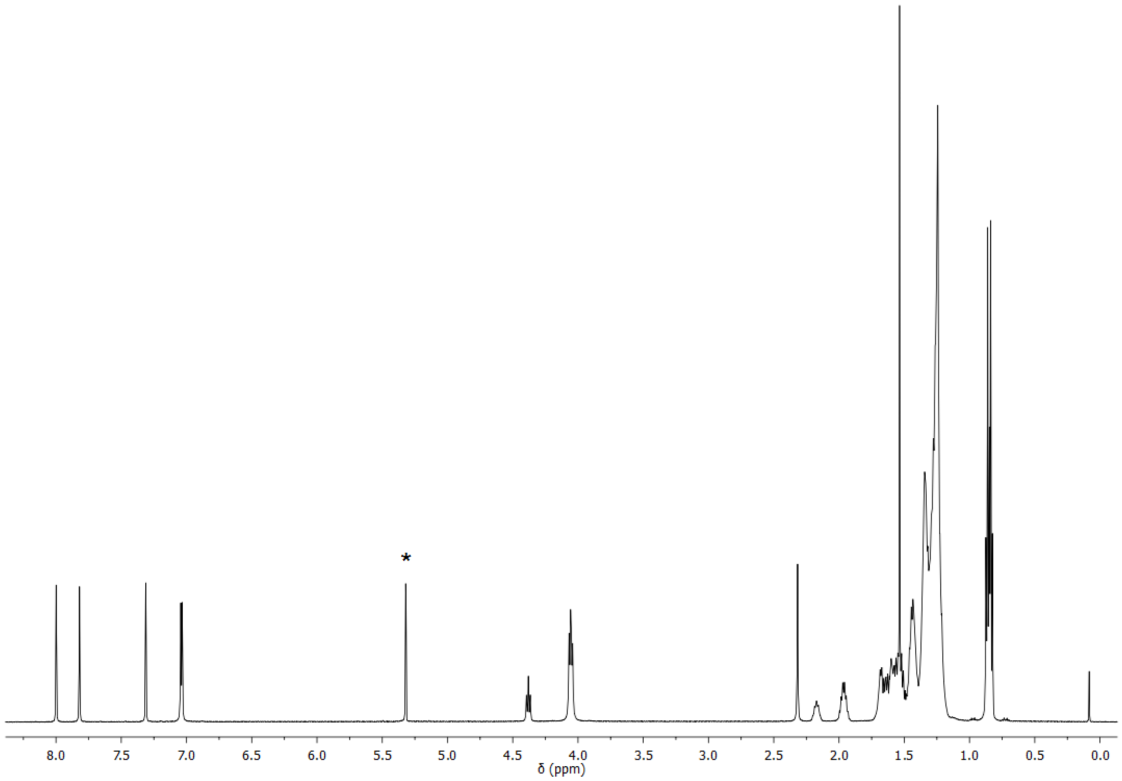

Figure S36: ^{1}H NMR (500 MHz, CD_2Cl_2) of **1a** (* = NMR solvent residual peak).

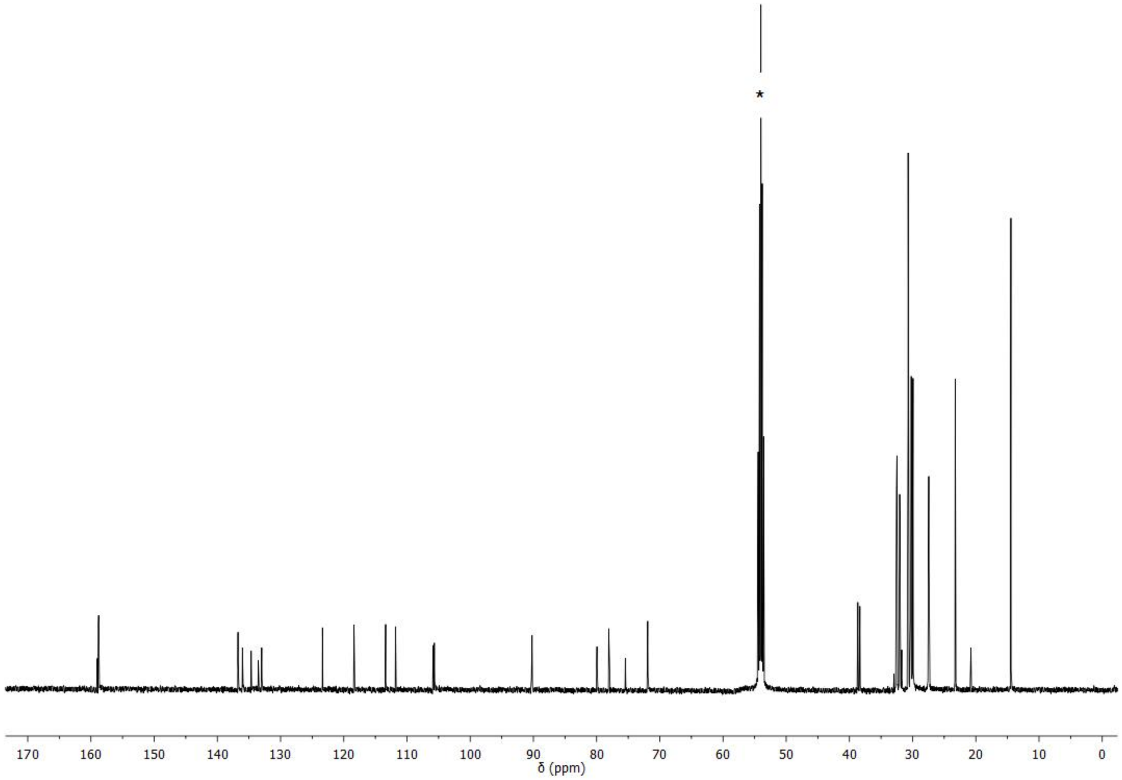

Figure S37: ^{13}C NMR (125 MHz, CD_2Cl_2) of **1a** (* = NMR solvent residual peak).

2.2 Synthesis and characterization of macrocycles 1b and 1c

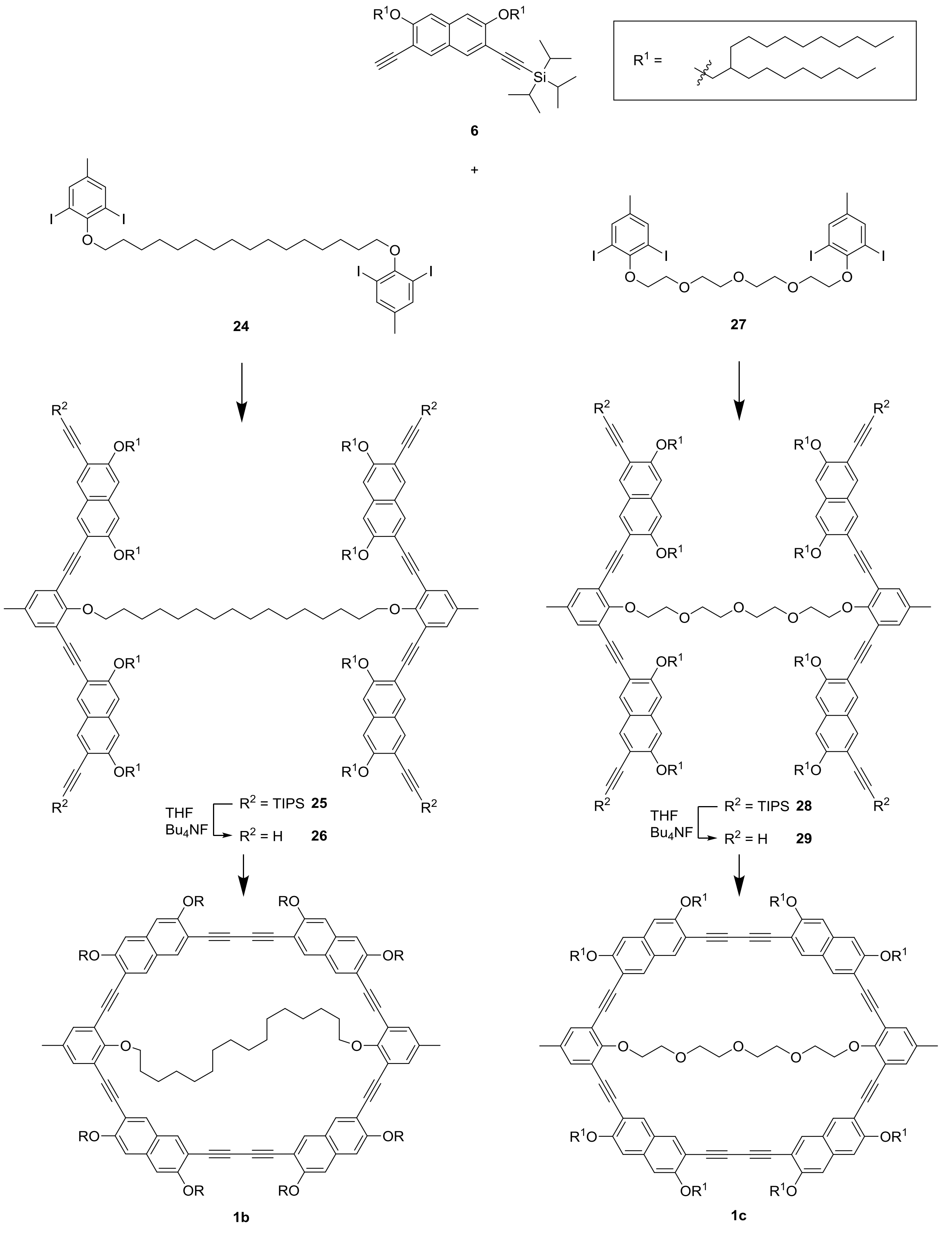

Scheme S3: Reaction scheme towards macrocycles **1b** and **1c**.

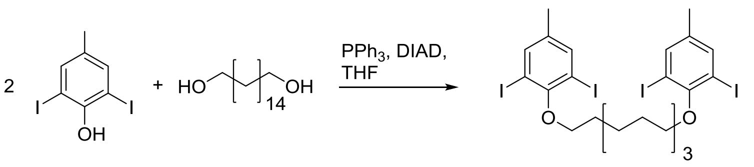

Scheme S4: Reaction scheme towards the central building block **24**.

Synthesis of 24. 2,6-Diiodo-4-methylphenol (**30**)[5] (260 mg, 0.73 mmol), PPh_3 (0.29 g, 1.10 mmol), and 1,16-hexadecanediol (100 mg, 0.38 mmol) were dissolved in THF (5 mL). After adding DIAD (0.22 g, 1.10 mmol) the mixture was stirred for 1 h at room temperature. The reaction mixture was diluted with CH_2Cl_2 and water. The organic layer was washed with water (3×) and brine. After drying ($MgSO_4$), the solvent was removed under reduced pressure. The residue was purified by column chromatography (silica gel, petroleum ether : CH_2Cl_2 = 3 : 1, R_f = 0.70) to yield **24** (311 mg, 0.35 mmol, 92%) as a colorless solid. M.p. 104 °C. ^{1}H NMR (400 MHz, $CDCl_3$): δ [ppm] 7.57 (s, 4H), 3.92 (t, J = 6.6 Hz, 4H), 2.23 (s, 6H), 1.97–1.82 (m, 4H), 1.64–1.46 (m, 4H), 1.46–1.19 (m, 20H). ^{13}C NMR (75 MHz, $CDCl_3$): δ [ppm] = 155.96, 140.40, 137.62, 90.70, 73.60, 30.18, 29.83, 29.79, 29.75, 29.68, 26.13, 19.76. MS (EI, 70 eV): m/z = 942.0 (60) $[M]^{\cdot+}$, 816.1 (11) $[M - I]^{\cdot+}$, 359.9 (100) $[M - C_{23}H_{37}I_2O]^{\cdot+}$, $C_{30}H_{42}I_4O_2$ requires 941.94.

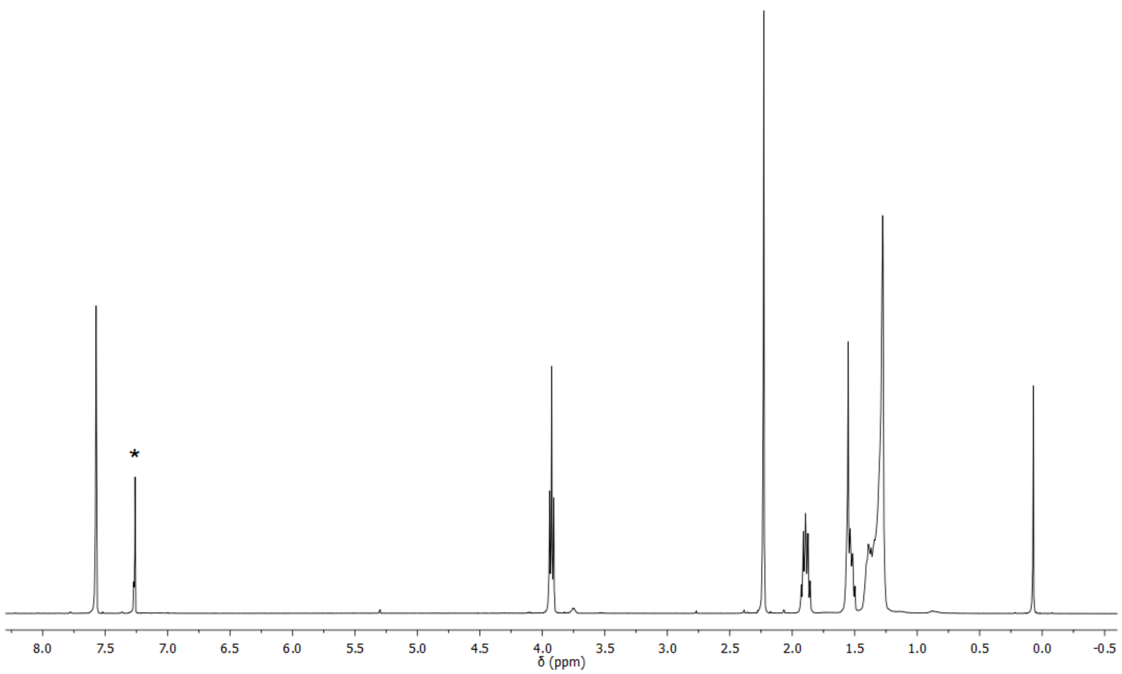

Figure S8: ^{1}H NMR (400 MHz, $CDCl_3$) of **24** (* = NMR solvent residual peak).

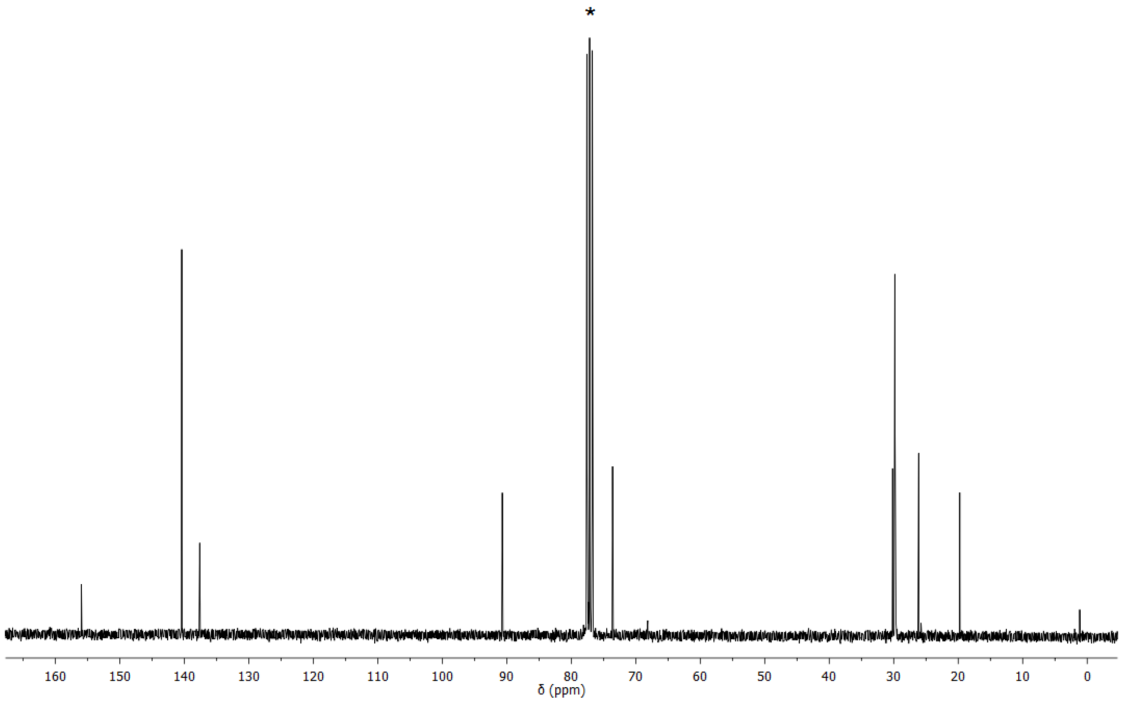

Figure S39: ^{13}C NMR (75 MHz, $CDCl_3$) of **24** (* = NMR solvent residual peak).

Synthesis of the TIPS-protected tetraacetylene 25. Under an argon atmosphere, **24** (35.7 mg, 37.9 µmol), $Pd(PPh_3)Cl_2$ (2.8 mg, 4.0 µmol), PPh_3 (6.0 mg, 22.9 µmol), and CuI (1.8 mg, 9.5 µmol) were poured into a Schlenk tube and a solution of **6** (180 mg, 194 µmol) in dry piperidine (2 mL) was added. The mixture was stirred at 70 °C for 18 h and—after cooling to room temperature—diluted with CH_2Cl_2 and water. The organic layer was washed with water (3×), aqueous acetic acid (10%, v/v, 2×), water, aqueous NaOH (10%, w/w), and brine. After drying ($MgSO_4$), the solvent was removed under reduced pressure. The crude product was purified by column chromatography (silica gel, petroleum ether : CH_2Cl_2 = 5 : 1, R_f = 0.40) yielding **25** as a colorless oil (140 mg, 33.9 µmol, 91%). ^{1}H NMR (300 MHz, $CDCl_3$): δ [ppm] = 7.81 (s, 4H), 7.80 (s, 4H), 7.28 (d, J = 0.5 Hz, 4H), 6.96 (s, 4H), 6.93 (s, 4H), 4.36 (t, J = 6.4 Hz, 4H), 3.99 (d, J = 5.5 Hz, 8H), 3.94 (d, J = 5.1 Hz, 8H), 2.29 (s, 6H), 2.03–1.75 (m, 12H), 1.69–0.99 (m, 364H), 0.96–0.78 (m, 48H). ^{13}C NMR (75 MHz, $CDCl_3$) : δ [ppm] = 158.95, 158.36, 158.01, 135.79, 134.14, 133.95, 132.74, 132.37, 122.88, 118.01, 112.86, 112.79, 105.22, 105.00, 103.39, 94.20, 90.07, 89.78, 71.51, 71.11, 38.26, 38.16, 32.07, 32.06, 31.62, 31.30, 30.87, 30.28, 30.24, 30.12, 30.07, 29.87, 29.80, 29.52, 27.11, 27.03, 26.66, 22.83, 20.53, 18.92, 14.25, 11.58. MALDI-MS (DCTB): m/z (%) = 4133.2 (100) $[M]^{\cdot+}$, $C_{282}H_{470}O_{10}Si_4$ requires 4129.53.

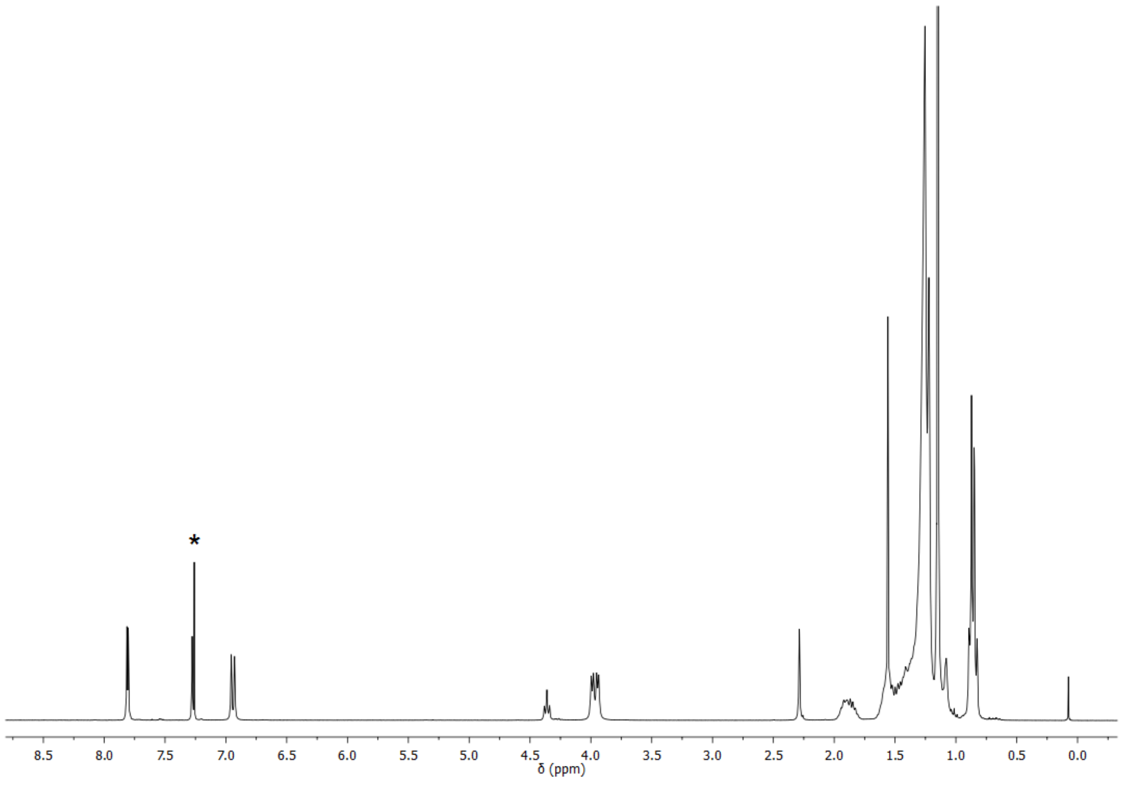

Figure S40: ^{1}H NMR (300 MHz, $CDCl_3$) of **25** (* = NMR solvent residual peak).

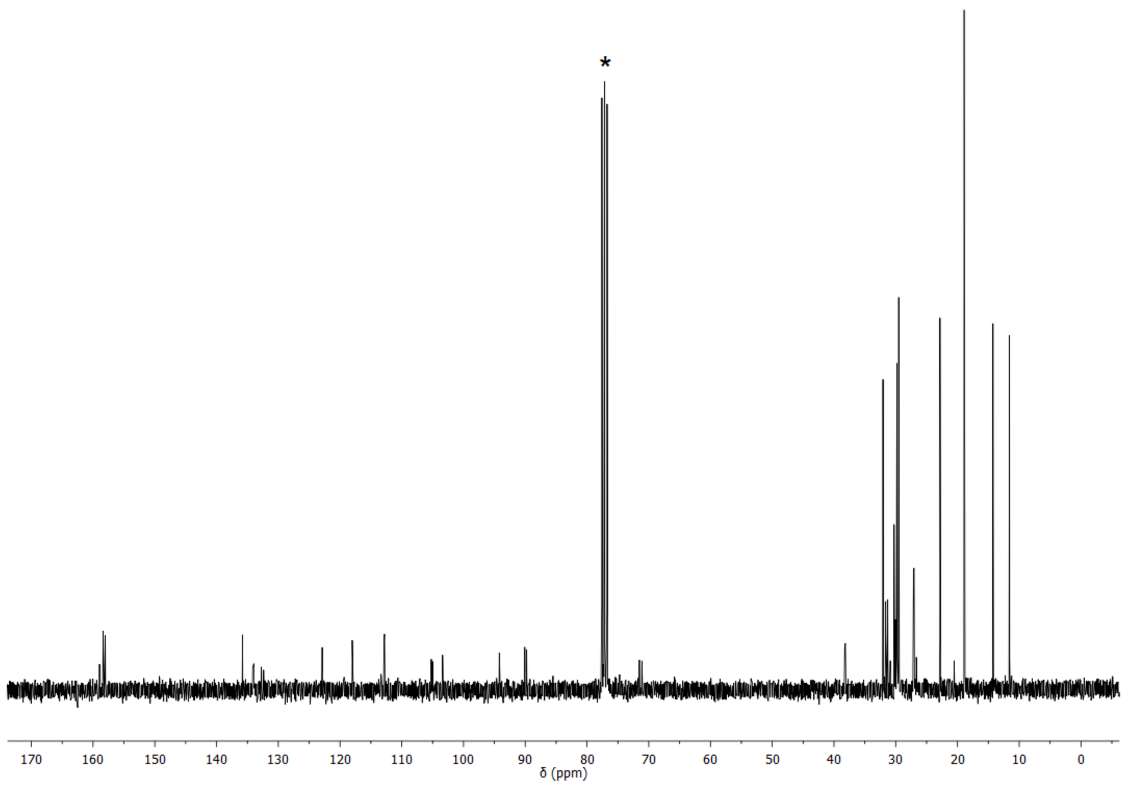

Figure S41: ^{13}C NMR (75 MHz, $CDCl_3$) of **25** (* = NMR solvent residual peak).

Synthesis of the tetraacetylene 26. 25 (137 mg, 33.1 µmol) was dissolved in THF (2 mL) and TBAF (1 M in THF, 0.40 mL) was added. The mixture was stirred for 2 h at room temperature and then diluted with CH_2Cl_2 and water. The organic layer was separated and washed with water (3x) and brine. After drying over $MgSO_4$, the solvent was evaporated. The residue was purified by column chromatography (silica gel, petroleum ether : CH_2Cl_2 = 4 : 1, R_f = 0.5 (petroleum ether : CH_2Cl_2 = 3 : 1)) to yield **26** as a turbid, yellowish oil (92.6 mg, 26.4 µmol, 79%). ^1H NMR (500 MHz, $CDCl_3$): δ [ppm] = 7.830 (s, 4H), 7.826 (s, 4H), 7.28 (s, 4H), 6.97 (s, 4H), 6.96 (s, 4H), 4.35 (t, J = 6.3 Hz, 4H), 3.99 (d, J = 5.7 Hz, 8H), 3.96 (d, J = 5.8 Hz, 8H), 3.22 (s, 4H), 2.29 (s, 6H), 1.98–1.83 (m, 12H), 1.67–1.16 (m, 280H), 0.93–0.78 (m, 48H). ^{13}C NMR (125 MHz, $CDCl_3$): 159.05, 158.30, 158.15, 135.96, 134.10, 134.05, 132.86, 132.47, 122.82, 117.98, 113.05, 111.46, 105.33, 105.20, 89.95, 89.82, 80.73, 80.20, 74.74, 71.68, 71.53, 38.11, 37.89, 32.08, 32.06, 31.59, 30.91, 30.28, 30.19, 30.15, 30.08, 30.05, 29.87, 29.85, 29.81, 29.75, 29.52, 27.10, 27.00, 26.78, 22.84, 22.83, 20.55, 14.27, 14.26. MALDI-MS (DCTB): m/z (%)= 3758.1 (< 5) $[M + DCTB]^{\cdot+}$, 3507.9 (100) $[M]^{\cdot+}$, 3227.9 (10) $[M - C_{20}H_{41}]^{\cdot+}$, $C_{246}H_{390}O_{10}$ requires 3505.61.

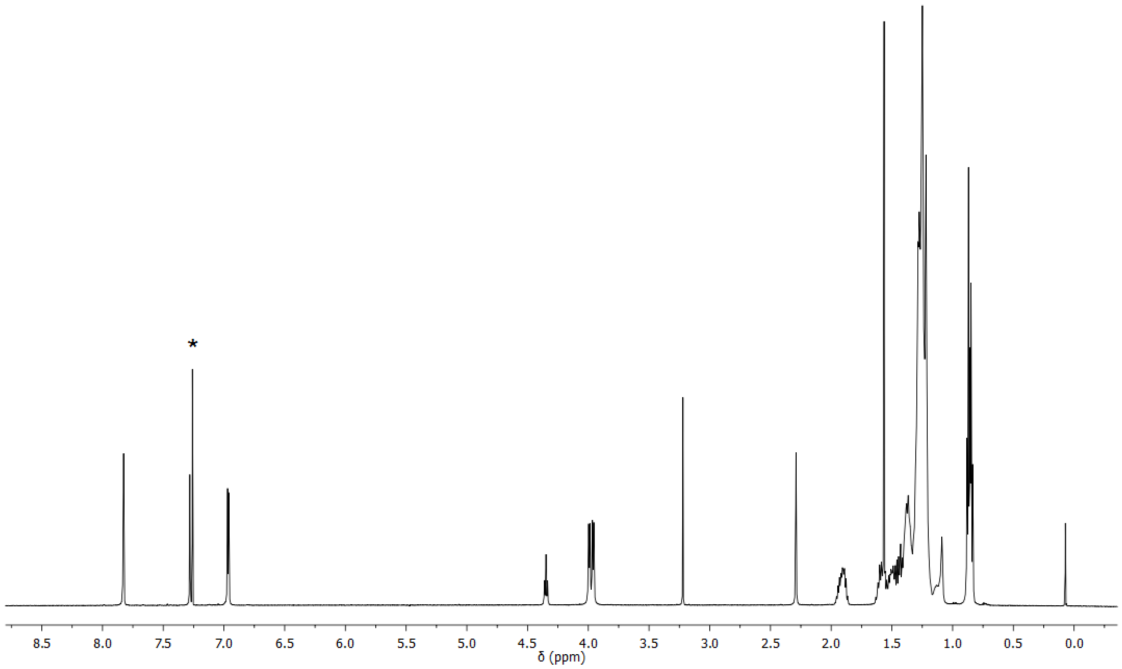

Figure S42: ^{1}H NMR (500 MHz, $CDCl_3$) of **26** (* = NMR solvent residual peak).

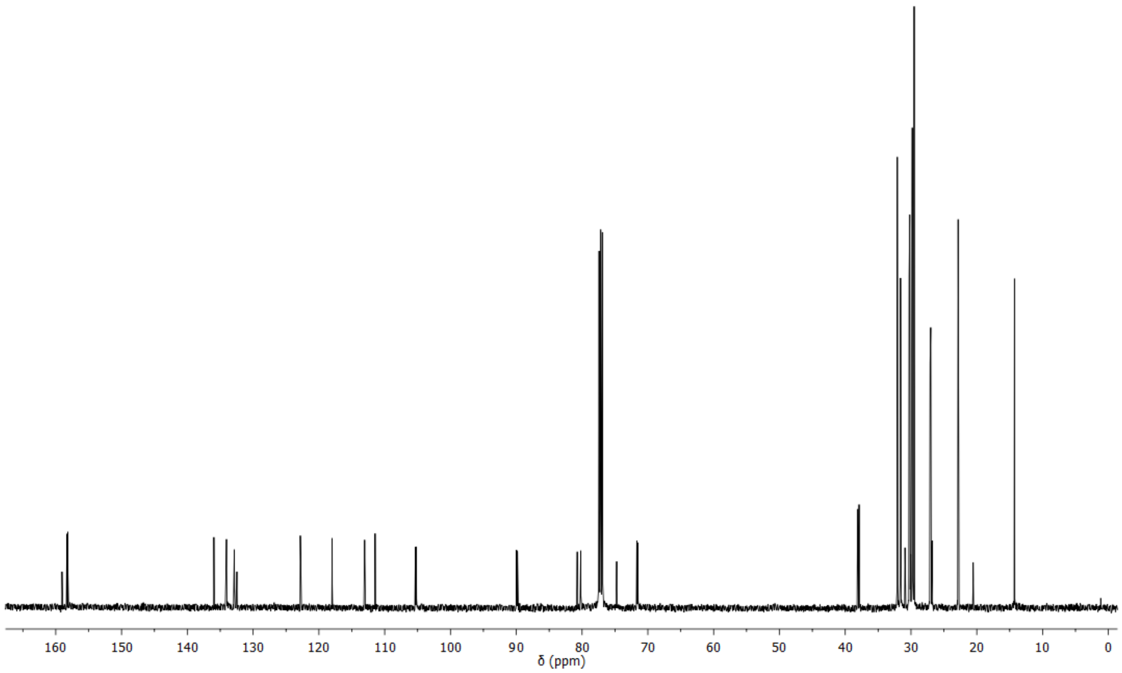

Figure S43: ^{13}C NMR (125 MHz, $CDCl_3$) of **26** (* = NMR solvent residual peak).

Synthesis of the TIPS-protected tetraacetylene 28. Under an argon atmosphere, $Pd(PPh_3)Cl_2$ (10.4 mg, 14.8 µmol), PPh_3 (13.5 mg, 51.5 µmol), and CuI (3.0 mg, 15.8 µmol) were poured into a Schlenk tube and **27** (100 mg, 114 µmol) and **6** (526 mg, 568 µmol), both separately dissolved in piperidine/THF (3.5 mL, 5:2), were subsequently added. The reaction mixture was stirred at 60 °C for 6.5 h and—after cooling to room temperature—diluted with CH_2Cl_2 and water. The organic layer was washed with water (3×), aqueous acetic acid (10%, v/v, 3×), water, aqueous NaOH (10%, w/w), and brine. After drying ($MgSO_4$), the solvent was removed under reduced pressure. The crude product was purified by column chromatography (silica gel, CH_2Cl_2 : MeOH = 100 : 1; R_f = 0.23, CH_2Cl_2 : MeOH = 200 : 1) yielding **28** as a colorless oil (301 mg, 74.0 µmol, 65%). In addition, a fraction of 176 mg is obtained, which contains mainly product material together with some impurities. ^{1}H NMR (500 MHz, $CDCl_3$): δ [ppm] = 7.80 (s, 8H), 7.24 (d, J = 0.6 Hz, 4H), 6.94 (s, 4H), 6.92 (s, 4H), 4.51 (t, J = 5.4 Hz, 4H), 4.02–3.84 (m, 20H), 3.62 (dd, J = 5.7 Hz, J = 4.3 Hz, 4H), 3.42 (dd, J = 5.6 Hz, J = 4.3 Hz, 4H), 2.26 (s, 6H), 1.95–1.78 (m, 8H), 1.62–1.49 (m, 16H), 1.48–1.16 (m, 240H), 1.16–1.09 (m, 84H), 0.92–0.79 (m, 48H). ^{13}C NMR (125 MHz, $CDCl_3$): δ [ppm] = 158.66, 158.35, 157.95, 135.79, 134.04, 133.98, 132.75, 132.54, 122.85, 117.91, 112.75, 112.73, 105.19, 104.95, 103.36, 94.16, 90.35, 89.56, 73.21, 71.47, 71.05, 70.75, 70.70, 70.67, 38.27, 38.09, 32.07, 31.55, 31.27, 30.28, 30.25, 29.88, 29.86, 29.83, 29.81, 29.55, 29.52, 27.09, 27.04, 22.84, 22.82, 20.52, 18.92, 14.27, 11.56. MALDI-MS (DCTB): m/z = 4068.9 $[M]^{\cdot+}$, $C_{274}H_{454}O_{13}Si_4$ requires 4065.39.

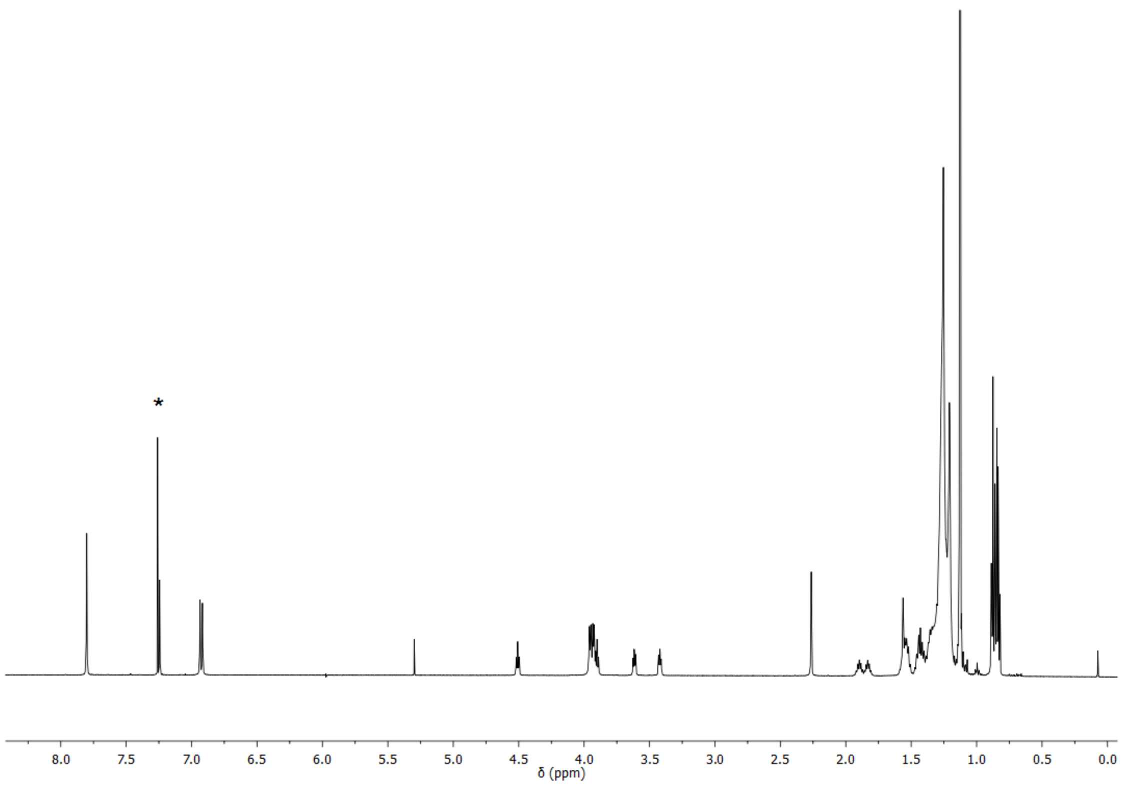

Figure S44: ^{1}H NMR (500 MHz, $CDCl_3$) of **28** (* = NMR solvent residual peak).

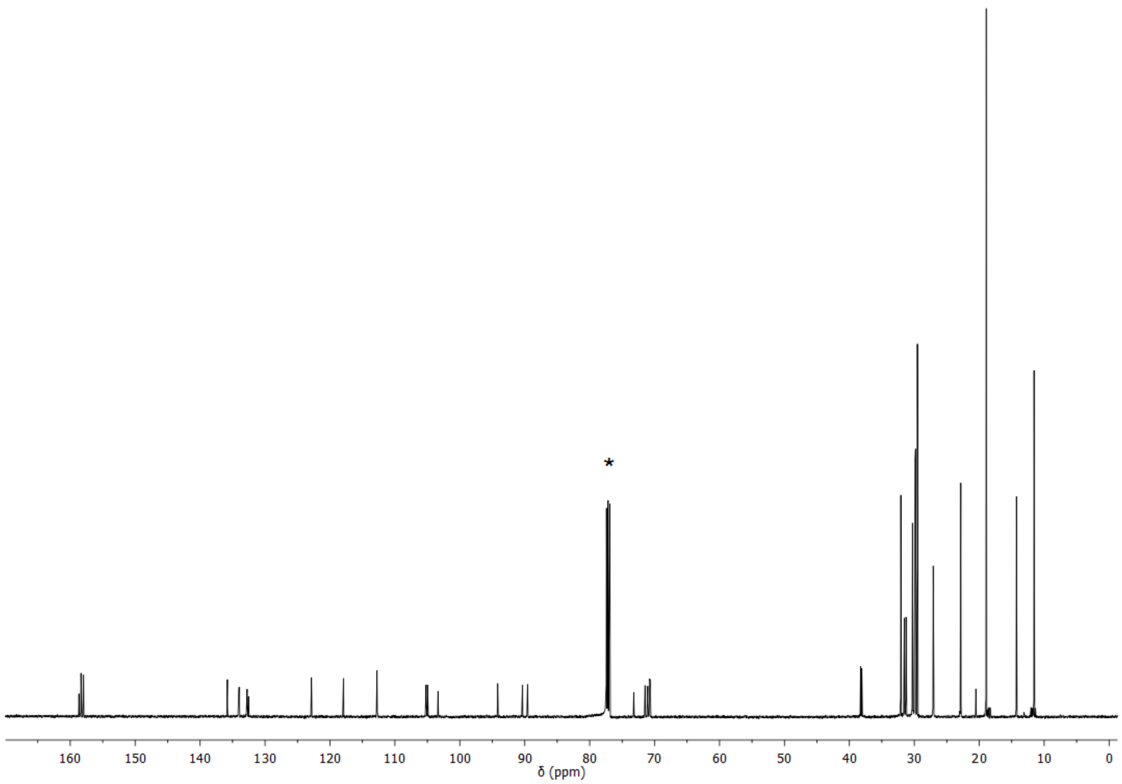

Figure S45: ^{13}C NMR (125 MHz, $CDCl_3$) of **28** (* = NMR solvent residual peak).

Synthesis of the tetraacetylene 29. 28 (292 mg, 71.8 µmol) was dissolved in THF (2 mL) and TBAF (1 M in THF, 0.70 mL) was added. The mixture was stirred for 18 h at room temperature. The reaction mixture was diluted with CH_2Cl_2 and water. The organic layer was separated and washed with water (3×) and brine. After drying over $MgSO_4$, the solvent was evaporated. The residue was purified by column chromatography (silica gel, CH_2Cl_2 : MeOH = 100 : 1, R_f = 0.5 (DCM)) to yield **29** as a dark yellow oil (176 mg, 51.1 µmol, 71%). ^{1}H NMR (400 MHz, $CDCl_3$): δ [ppm] = 7.84 (s, 4H), 7.82 (s, 4H), 7.26 (d, J = 0.5 Hz, 4H), 6.95 (s, 4H), 6.94 (s, 4H), 4.51 (t, J = 5.2 Hz, 4H), 4.05–3.89 (m, 20H), 3.69 (dd, J = 5.8 Hz, J = 4.5 Hz, 4H), 3.52 (dd, J = 5.7 Hz, J = 4.5 Hz, 4H), 3.21 (s, 4H), 2.28 (s, 6H), 1.98–1.83 (m, 8H), 1.65–1.14 (m, 256H), 0.94–0.79 (m, 48H). ^{13}C NMR (75 MHz, $CDCl_3$): δ [ppm] = 158.79, 158.31, 158.11, 135.98, 134.12, 133.99, 132.99, 132.67, 130.29, 122.86, 117.95, 112.95, 111.49, 105.35, 105.22, 90.32, 89.62, 80.84, 80.23, 73.40, 71.70, 71.57, 70.84, 70.71, 38.11, 37.93, 32.08, 32.06, 31.59, 30.29, 30.20, 29.87, 29.81, 29.76, 29.52, 27.10, 27.02, 22.84, 22.82, 20.54, 17.85, 14.26. MALDI-MS (DCTB): m/z (%) = 3693.9 (8) $[M + DCTB]^{+\cdot}$, 3466.6 (14) $[M + Na]^{+\cdot}$, 3443.5 (100) $[M]^{+\cdot}$, 3162.5 (< 5) $[M - C_{20}H_{41}]^{+\cdot}$, $C_{238}H_{374}O_{13}$ requires 3440.86.

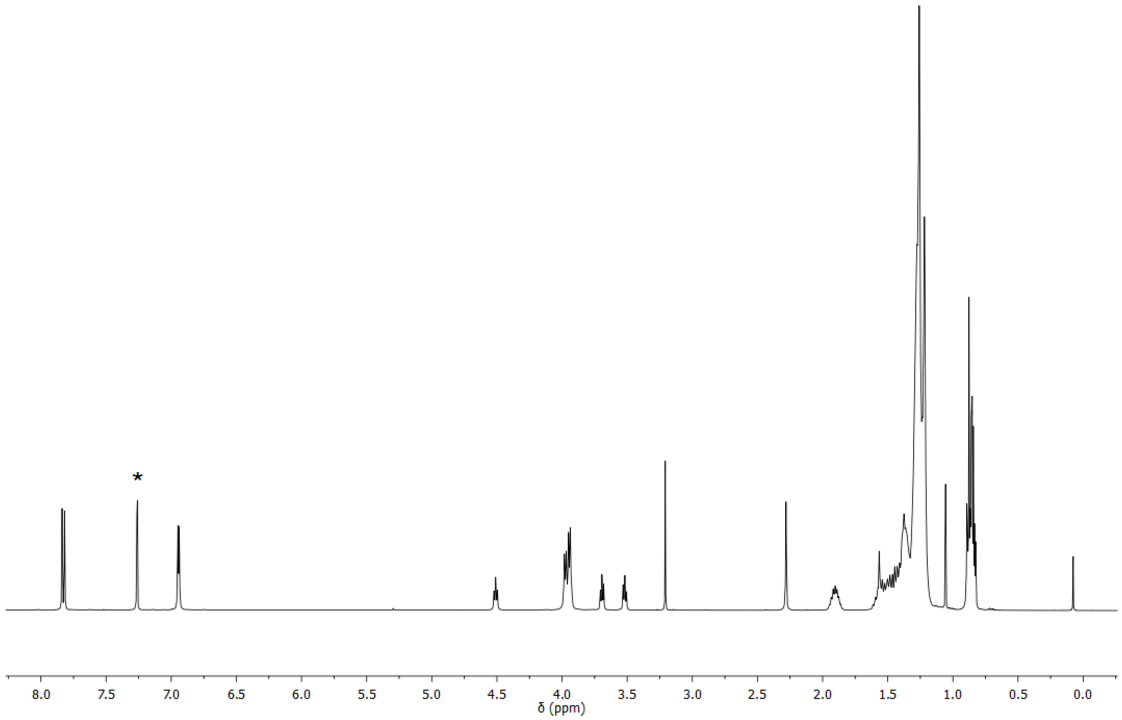

Figure S46: ^{1}H NMR (400 MHz, $CDCl_3$) of **29** (* = NMR solvent residual peak).

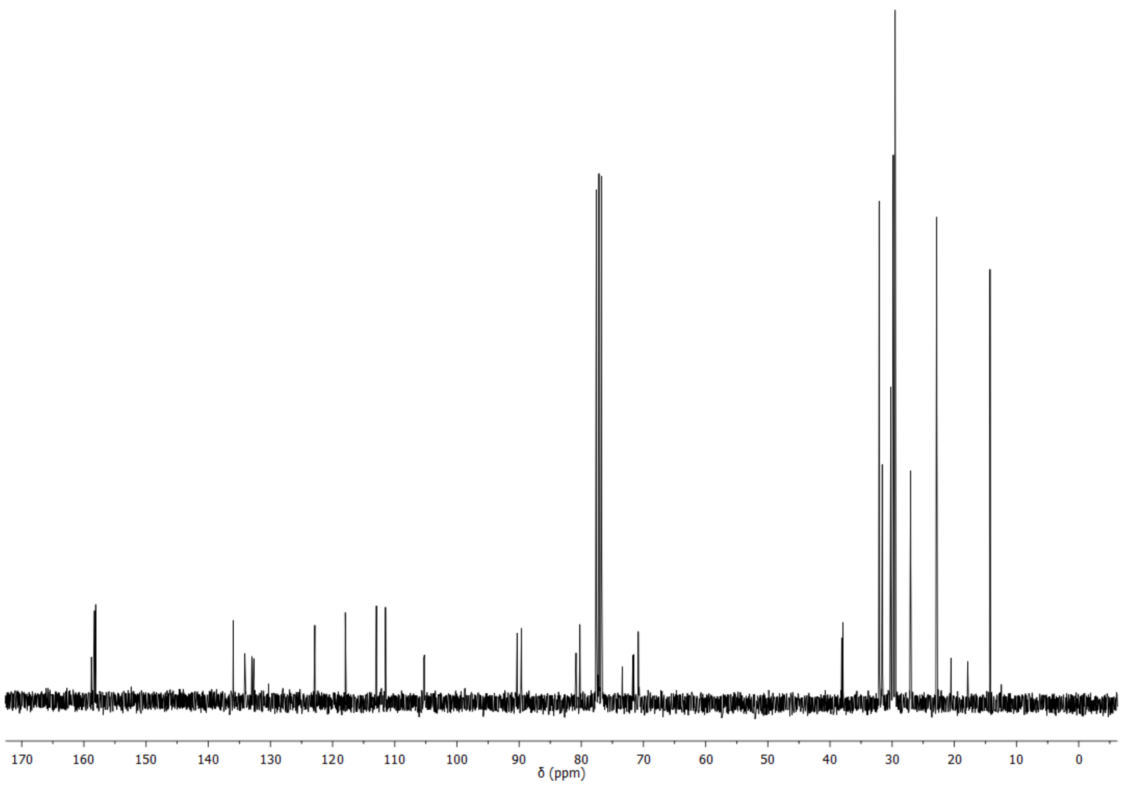

Figure S47: ^{13}C NMR (75 MHz, $CDCl_3$) of **29** (* = NMR solvent residual peak).

Synthesis of macrocycle 1b. Under an argon atmosphere, a 50 mL Hamilton syringe was charged with a solution of tetraacetylene **26** (34.2 mg, 9.75 µmol) in THF (20 mL). $Pd(PPh_3)_2Cl_2$ (4.0 mg, 5.70 µmol, 0.6 equiv) and CuI (2.7 mg, 14.2 µmol, 1.5 equiv) together with 1,4-benzoquinone (11.0 mg, 61.1 µmol, 6.3 equiv) were dissolved in THF (15 mL) and piperidine (15 mL) and heated to 50 °C. While stirring vigorously, the acetylene solution was added dropwise to the catalyst/oxidant solution over a period of 48 h. The mixture was stirred for additional 16 h at 50 °C. After letting the reaction mixture cool to room temperature, it was diluted with CH_2Cl_2 and water. The organic layer was washed with water (3×), acetic acid (10% v/v, 3×), water, NaOH (10% w/w), and brine, and subsequently dried over $MgSO_4$. The solvent was removed under reduced pressure. In a first cleaning step, the residue was separated from the inorganic impurities by column chromatography (silica gel, petroleum ether : CH_2Cl_2 = 3 : 1). The resulting crude product was purified by preparative recycling GPC. The solvent of the product fraction was removed under reduced pressure. To remove of the THF stabilizer, the oily residue was finally purified by column chromatography (silica gel, petroleum ether) to yield **1b** as a slightly yellow, soft material which crystallizes after several days (8.2 mg, 2.34 µmol, 24%). M.p. 72 °C. ^1H NMR (400 MHz, $CDCl_3$, 298 K): δ [ppm] = 7.92 (s, 4H), 7.90 (s, 4H), 7.27 (d, J = 0.4 Hz, 4H), 6.98 (s, 4H), 6.98 (s, 4H), 4.32 (t, J =5.9 Hz,

4H), 4.01 (dd, J = 8.6 Hz, J = 5.7 Hz, 16H), 2.31 (s, 6H), 2.08–1.88 (m, 12H), 1.86–1.74 (m, 4H), 1.72–1.12 (m, 276H), 0.93–0.77 (m, 48H). 13C NMR (126 MHz, CD_2Cl_2, 298 K): δ [ppm] = 159.93, 159.17, 158.65, 136.72, 135.07, 133.89, 133.60, 133.42, 131.47, 129.28, 123.38, 118.60, 113.41, 112.04, 105.87, 105.71, 90.61, 90.01, 78.99, 78.28, 75.50, 72.03, 71.81, 38.65, 38.34, 32.54, 32.51, 32.49, 32.12, 32.09, 31.29, 30.74, 30.72, 30.39, 30.36, 30.31, 30.27, 30.26, 30.22, 30.17, 30.09, 30.02, 30.00, 29.94, 29.75, 29.61, 29.28, 27.56, 27.54, 27.47, 27.44, 27.42, 27.24, 23.27, 23.26, 23.24, 20.86, 14.45. MALDI-MS (DCTB): m/z (%) = 7005.9 (<5) $[2M]^{+\cdot}$, 3754.3 (10) $[M+DCTB]^{+\cdot}$, 3503.7 (100) $[M]^{+\cdot}$. $C_{246}H_{386}O_{10}$ requires 3500.97. GPC (PS calibration): single peak at $M_{peak} = 4.46 \times 10^3$ g mol^{-1} ($M_w = 4.60 \times 10^3$ g mol^{-1}).

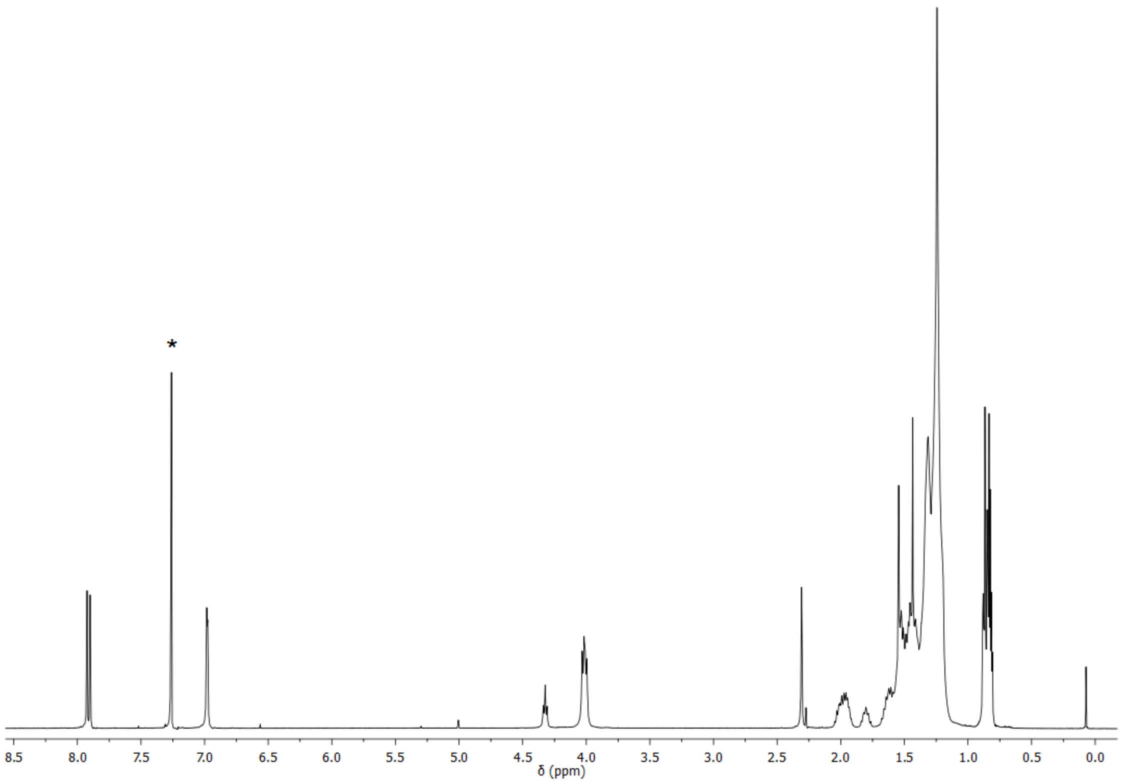

Figure S48: ^{1}H NMR (400 MHz, $CDCl_3$) of **1b** (* = NMR solvent residual peak).

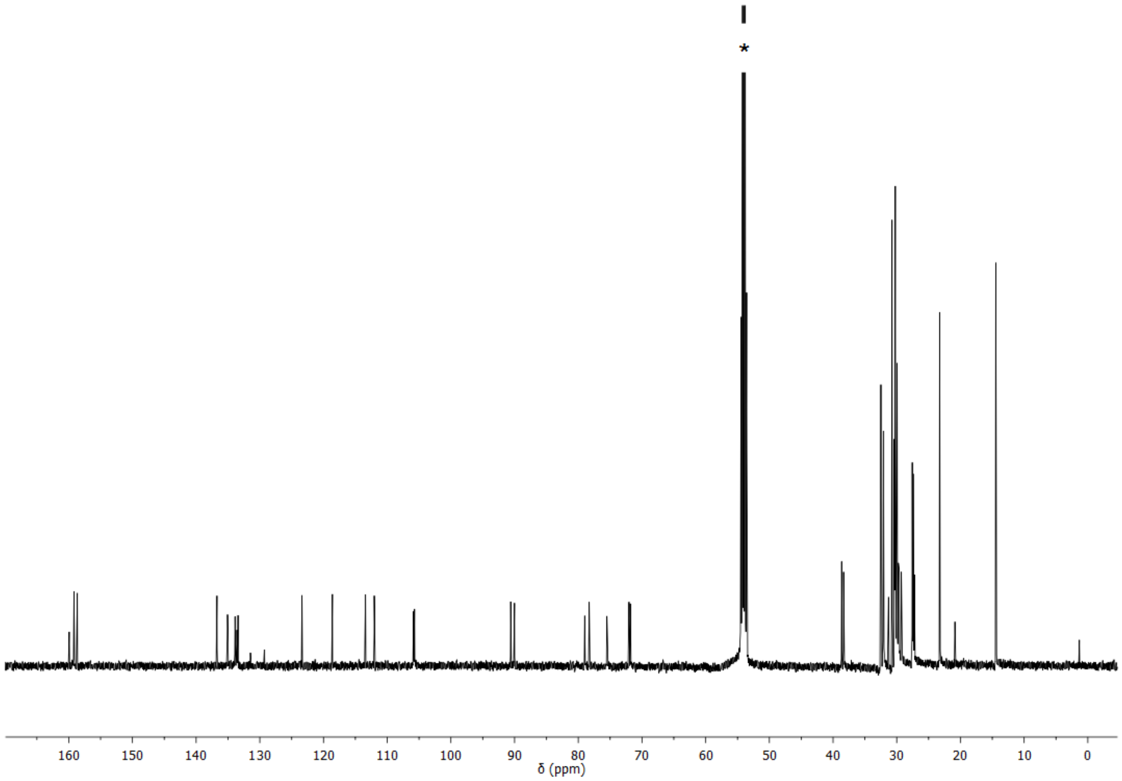

Figure S49: ^{13}C NMR (125 MHz, CD_2Cl_2) of **1b** (* = NMR solvent residual peak).

Synthesis of macrocycle 1c. Under an argon atmosphere, $Pd(PPh_3)_2Cl_2$ (17.2 mg, 24.50 µmol, 1.2 equiv) and CuI (5.5 mg, 28.9 µmol, 1.4 equiv) together with 1,4-benzoquinone (14.7 mg, 81.1 µmol, 4.1 equiv) were dissolved in piperidine (5 mL) and heated to 50 °C. While stirring vigorously, a solution of tetraacetylene **29** (68.8 mg, 20.0 µmol) in piperidine (5 mL) was slowly added to the catalyst/oxidant-solution. The mixture was stirred for 2:45 h at 50 °C. After allowing the reaction mixture to cool to room temperature, it was diluted with diethyl ether and water. The layers were separated and the aqueous phase was extracted trice with diethyl ether. The combined organic layers were washed with water (3×), acetic acid (10% v/v, 3×), water, NaOH (2 M), and brine, and subsequently dried over $MgSO_4$. The solvent was removed under reduced pressure. In a first cleaning step, the residue was separated from the inorganic impurities by column chromatography (silica gel, cyclohexane : CH_2Cl_2 = 3 : 1, R_f = 0.4). The resulting crude product was purified by preparative recycling GPC. The solvent of the product fraction was removed under reduced pressure. The residue was dissolved in CH_2Cl_2 and, by adding methanol, the product was precipitated from the BHT containing solution. The suspension was filtered through a PTFE membrane to yield **1c** as a slightly yellow solid (38.8 mg, 11.3 µmol, 56%). M.p. 89-105 °C (two polymorphs). ^{1}H NMR (400 MHz, CD_2Cl_2, 298 K): δ [ppm] = 8.05 (s, 4H), 7.86 (s, 4H), 7.34 (s, 4H), 7.05 (s,

4H), 7.03 (s, 4H), 4.57 (dd, J = 7.7, 5.9 Hz, 4H), 4.15 (dd, J = 7.7, 5.9 Hz, 4H), 4.11–4.00 (m, 16H), 3.96–3.85 (m, 8H), 2.32 (s, 6H), 2.03–1.91 (m, 8H), 1.74–1.15 (m, 256H), 0.91–0.79 (m, 48H). ^{13}C NMR (125 MHz, CD_2Cl_2, 298 K): δ [ppm] = 158.92, 158.51, 158.10, 136.78, 136.75, 135.27, 133.74, 132.89, 123.40, 118.02, 113.13, 111.84, 105.88, 105.63, 90.66, 90.07, 80.71, 78.21, 72.99, 72.15, 71.89, 71.49, 71.07, 70.47, 38.69, 38.33, 32.56, 32.52, 32.50, 32.49, 32.03, 32.00, 30.70, 30.37, 30.33, 30.32, 30.28, 30.26, 30.25, 30.23, 30.21, 30.01, 30.00, 29.94, 29.92, 27.53, 27.52, 27.45, 27.44, 23.29, 23.27, 23.25, 20.70, 14.46. MALDI-MS (DCTB): m/z (%) = 4190.0 (<5) $[M+3DCTB]^{\cdot+}$, 3940.0 (15) $[M+2DCTB]^{\cdot+}$, 3689.9 (50) $[M+DCTB]^{\cdot+}$, 3439.5 (100) $[M]^{\cdot+}$, 3172.5 (6) $[M–C_{19}H_{39}]^{\cdot+}$. $C_{238}H_{370}O_{13}$ requires 3436.83. GPC (PS calibration): single peak at $M_{peak} = 4.65 \times 10^3$ g mol^{-1}.

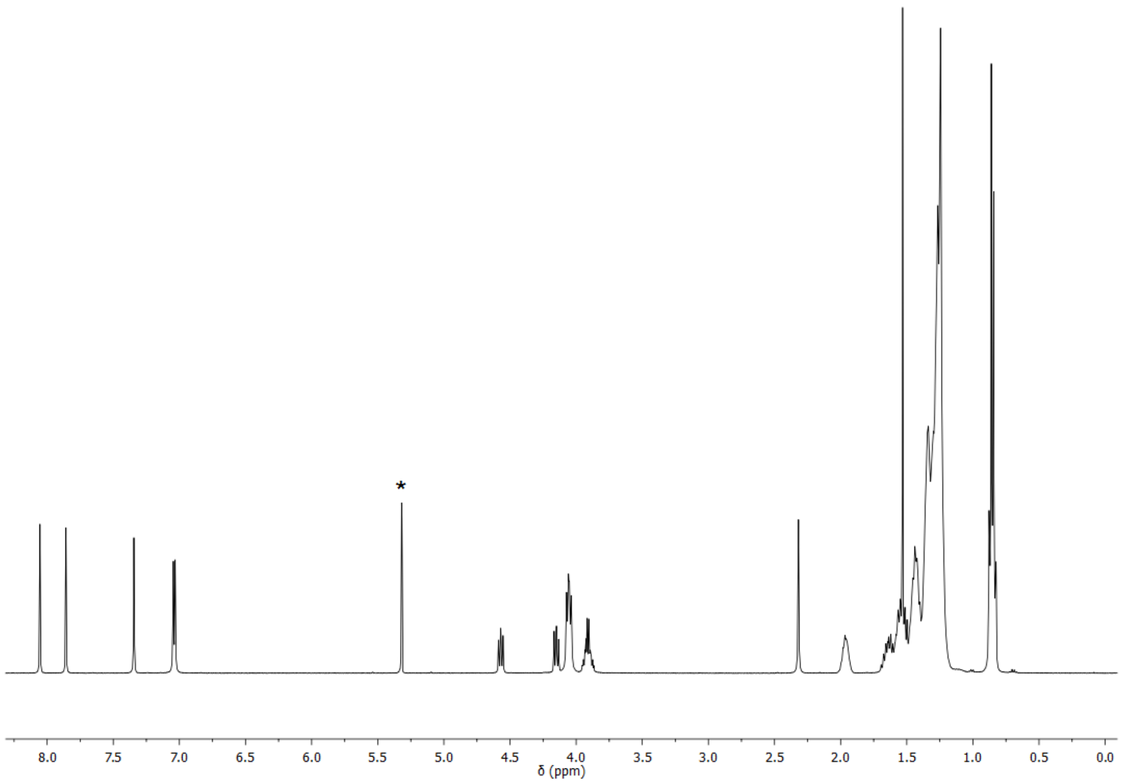

Figure S50: ^{1}H NMR (400 MHz, CD_2Cl_2) of **1c** (* = NMR solvent residual peak).

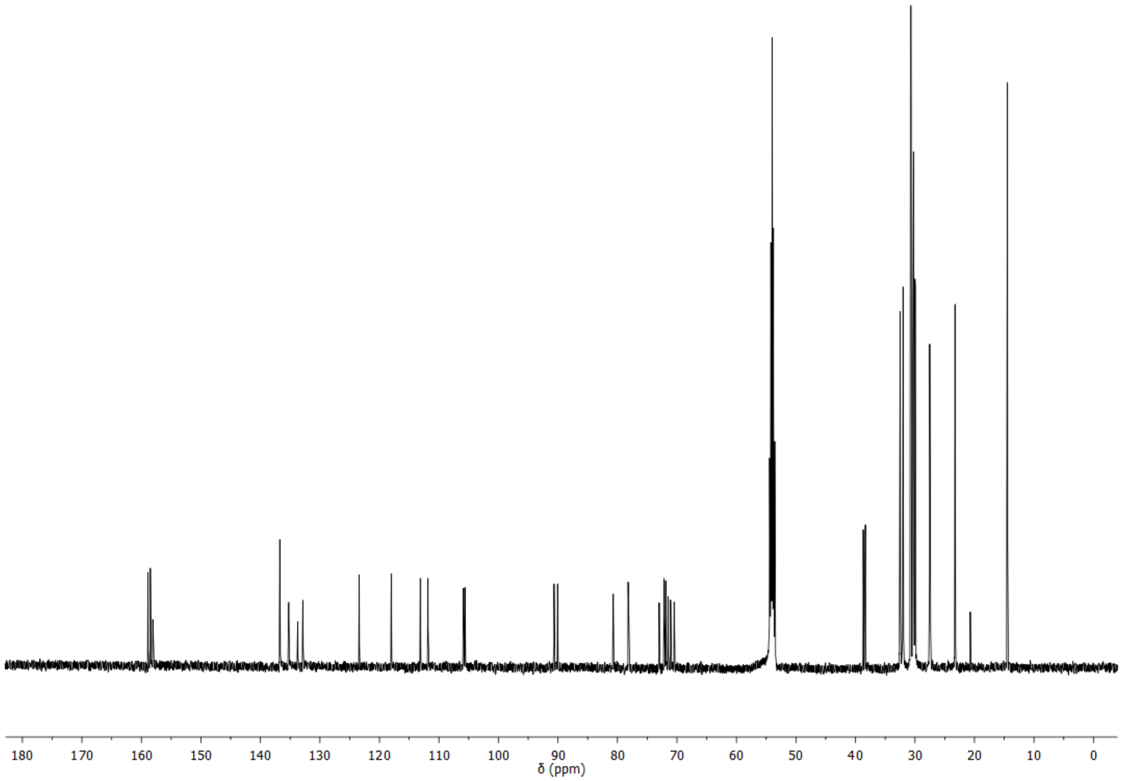

Figure S51: ^{13}C NMR (125 MHz, CD_2Cl_2) of **1c** (* = NMR solvent residual peak).

2.3 Synthesis and characterization of macrocycle 1d

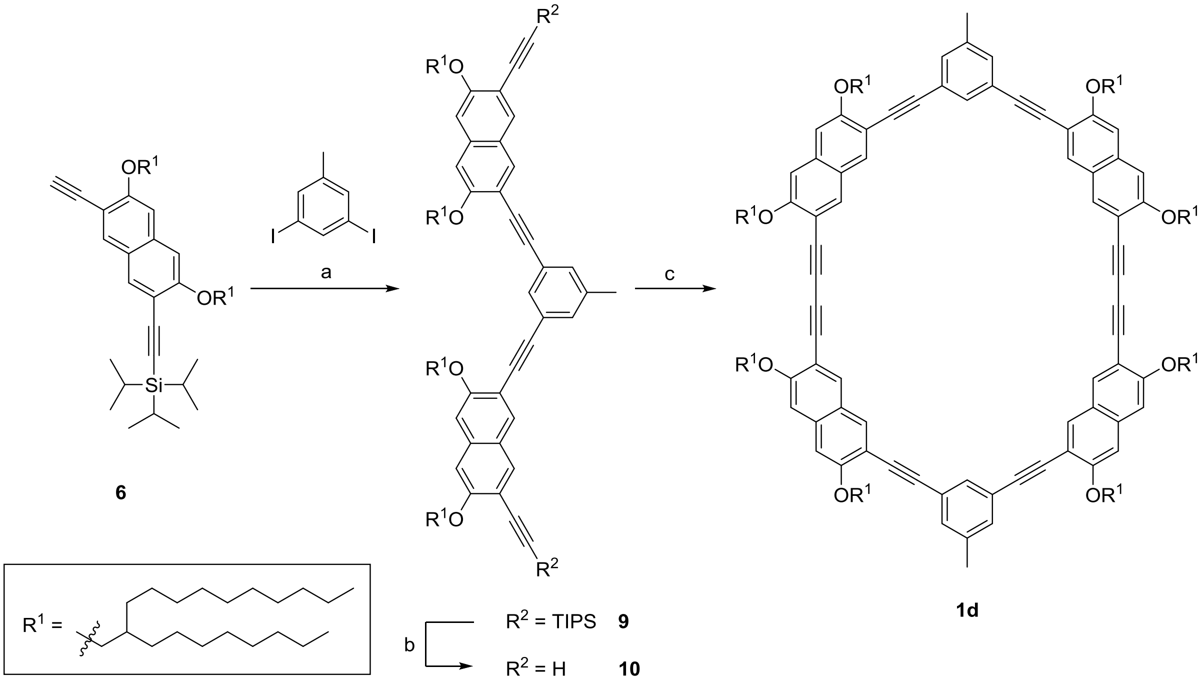

Scheme S5: Reaction pathway towards macrocycle **1d**.

Synthesis of the TIPS-protected half-ring 9. Dissolved in CH_2Cl_2, **6** (137 mg, 148 µmol) was poured into a Schlenk tube and the solvent was subsequently removed under reduced pressure. After applying an argon atmosphere, $Pd(PPh_3)Cl_2$ (3.4 mg, 4.84 µmol), PPh_3 (4.2 mg, 16.0 µmol), CuI (1.9 mg, 10.0 µmol), 3,5-diiodotoluene (20.2 mg, 58.7 µmol) and dry piperidine (1.0 mL) were added. The mixture was stirred at 40 °C for 23 h. After allowing the reaction mixture to cool to room temperature, water and CH_2Cl_2 were added. The organic layer was separated and washed with water (3×), aqueous acetic acid (10%, v/v, 3×), aqueous NaOH (10%, w/w), and brine and dried over $MgSO_4$. The solvent was removed under reduced pressure, and the residue was purified by column chromatography (silica gel, petroleum ether : CH_2Cl_2 = 15 : 1, R_f = 0.53) yielding **9** as a colorless oil (112 mg, 57.8 µmol, 98%). ^{1}H NMR (300 MHz, $CDCl_3$): δ [ppm] = 7.83 (s, 4H), 7.55 (s, 1H), 7.33 (d, J = 0.7 Hz, 2H), 6.97 (s, 2H), 6.95 (s, 2H), 4.01 (d, J = 5.6 Hz, 4H), 3.96 (d, J = 5.1 Hz, 4H), 2.37 (s, 3H), 2.04–1.77 (m, 4H), 1.70–1.12 (m, 170H), 0.94–0.79 (m, 24H). ^{13}C NMR (75 MHz, $CDCl_3$): δ [ppm] = 158.41, 158.07, 138.06, 135.86, 134.02, 132.86, 132.05, 131.68, 124.10, 122.87, 112.80, 112.53, 105.23, 105.02, 103.40, 94.27, 92.74, 86.37, 71.56, 71.09, 38.27, 38.20, 32.07, 31.73, 31.32, 30.29, 30.24, 29.86, 29.84, 29.82, 29.80, 29.54, 29.52, 27.16, 27.04, 22.84, 22.82, 21.23, 18.93, 14.26, 11.60. MALDI-MS (DCTB): m/z = 2187.8 (<5) $[M+DCTB]^{\cdot+}$, 1937.6 $[M]^{\cdot+}$. $C_{133}H_{220}O_4Si_2$ requires 1937.66.

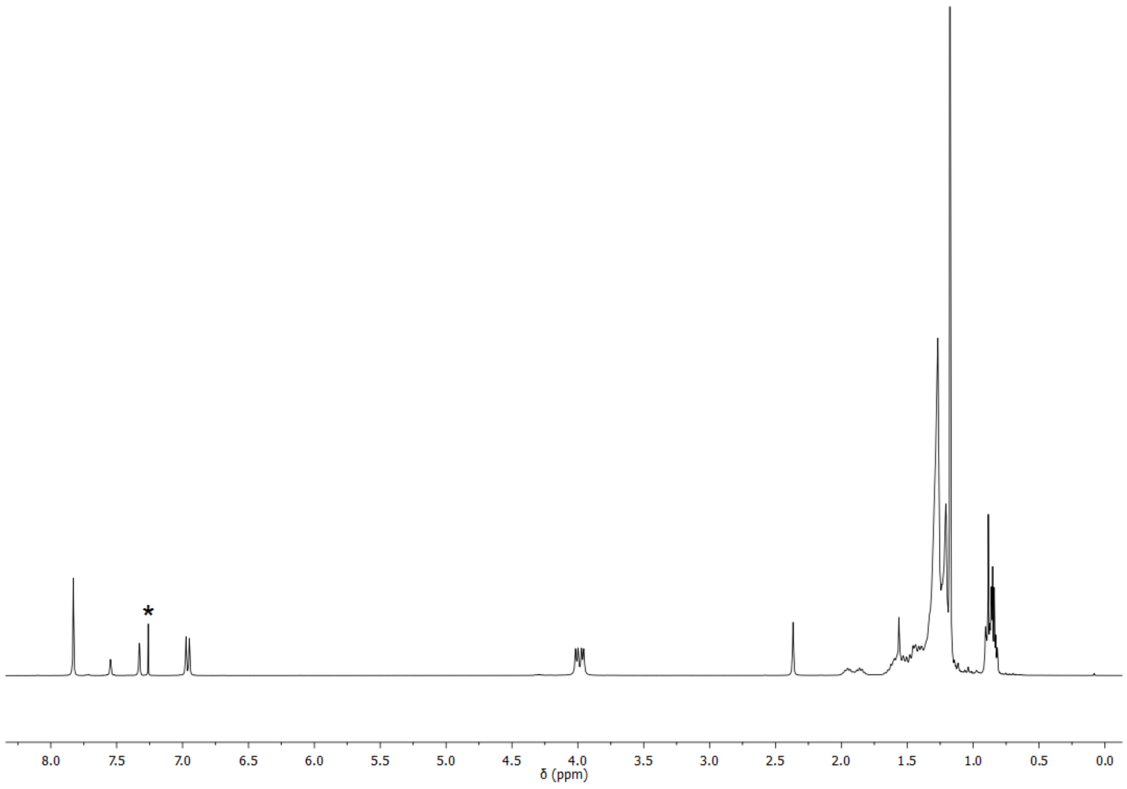

Figure S52: ^{1}H NMR (300 MHz, $CDCl_3$) of **9** (* = NMR solvent residual peak).

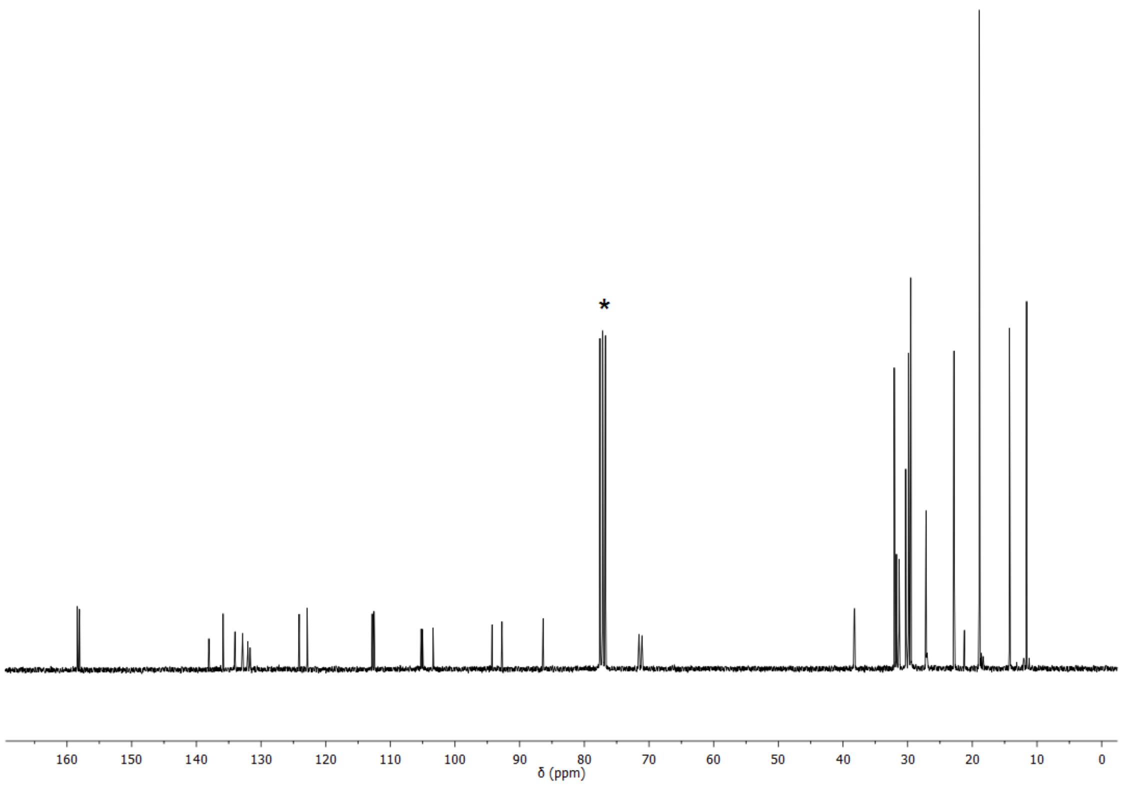

Figure S53: ^{13}C NMR (75 MHz, $CDCl_3$) of **9** (* = NMR solvent residual peak).

Synthesis of the bisacetylene 10. 9 (110 mg, 56.7 µmol) was dissolved in THF (1 mL), and TBAF (1 M in THF, 0.3 mL) was added. After stirring for 28 h at room temperature, the solvent was removed under reduced pressure. The residue was purified by column chromatography (silica gel, petroleum ether : CH_2Cl_2 = 5 : 1, R_f = 0.50) yielding **10** as a colorless solid (92.2 mg, 56.7 µmol, >99%). ^{1}H NMR (300 MHz, $CDCl_3$, 298 K): δ [ppm] = 7.85 (s, 2H), 7.83 (s, 2H), 7.55 (s, 1H), 7.33 (d, J = 0.6 Hz, 2H), 6.98 (s, 4H), 4.08–3.93 (m, 8H), 3.25 (s, 2H), 2.36 (s, 3H), 2.04–1.84 (m, 4H), 1.68–1.14 (m, 128H), 0.95–0.77 (m, 24H). ^{13}C NMR (75 MHz, $CDCl_3$): δ [ppm] = 158.37, 158.24, 138.10, 136.05, 134.08, 132.95, 132.06, 131.74, 124.04, 122.82, 112.78, 111.47, 105.38, 105.23, 92.86, 86.25, 80.71, 80.25, 71.74, 71.60, 38.18, 37.93, 32.08, 32.06, 31.71, 31.62, 30.29, 30.19, 29.85, 29.81, 29.79, 29.75, 29.52, 27.15, 27.01, 22.84, 22.83, 22.82, 21.23, 18.29, 14.27, 13.55. MALDI-MS (DCTB): m/z = 1625.4 $[M]^{\cdot+}$. $C_{115}H_{180}O_4$ requires 1625.39). GPC (PS calibration): single peak at $M_{peak} = 2.55 \times 10^3$ g mol^{-1} ($M_w = 2.62 \times 10^3$ g mol^{-1}).

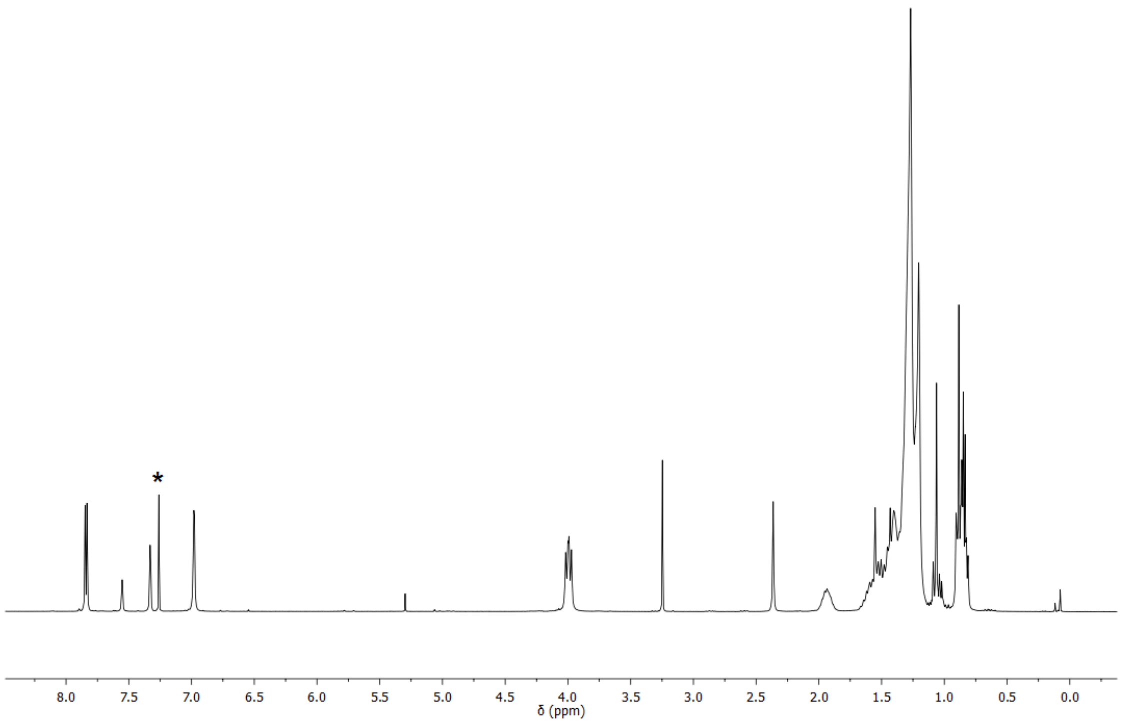

Figure S54: ^{1}H NMR (300 MHz, $CDCl_3$) of **10** (* = NMR solvent residual peak).

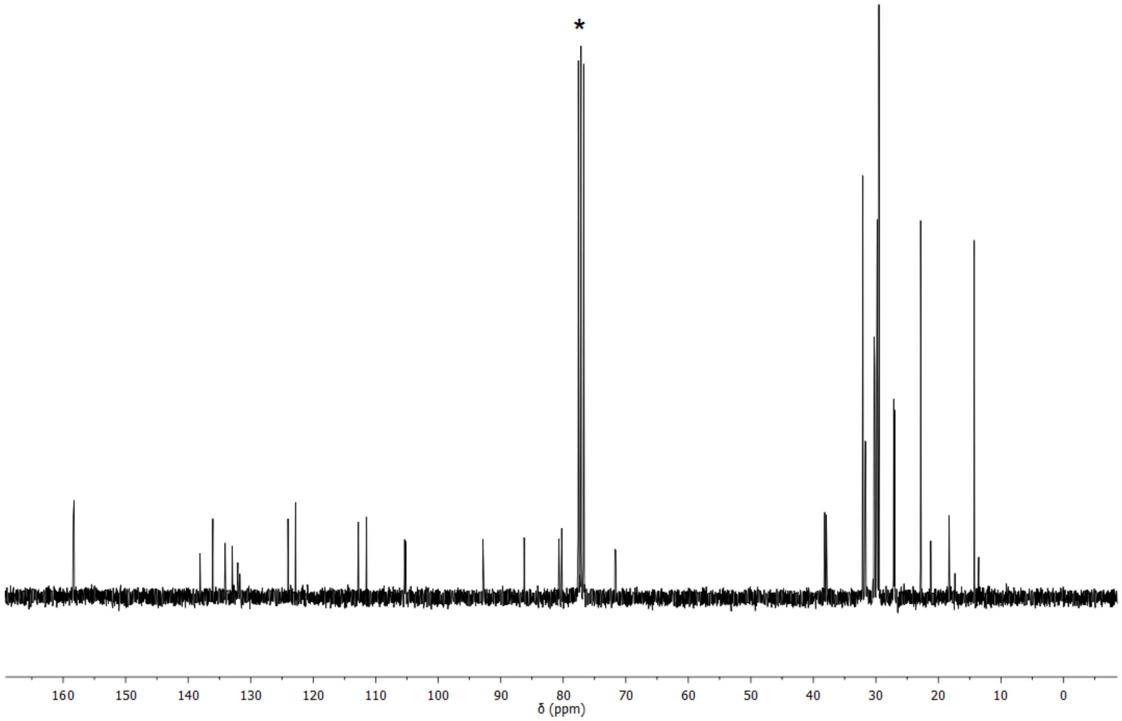

Figure S55: ^{13}C NMR (75 MHz, $CDCl_3$) of **10** (* = NMR solvent residual peak).

Synthesis of macrocycle 1d. Under an argon atmosphere, a 50 ml Hamilton syringe was charged with a solution of tetraacetylene **10** (78.1 mg, 48.0 µmol) in THF (40 mL). In a Schlenk flask, $Pd(PPh_3)_2Cl_2$ (17.5 mg, 24.9 µmol, 0.5 equiv), CuI (6.7 mg, 35.2 µmol, 0.7 equiv) and 1,4-benzoquinone (19.5 mg, 180.43 µmol, 3.8 equiv) were dissolved in THF (30 mL) and piperidine (30 mL) and heated to 50 °C. While stirring vigorously, the acetylene solution was added dropwise to the catalyst/oxidant solution over a period of 72 h. The mixture was stirred for additional 48 h at 50 °C. After allowing the reaction mixture to cool to room temperature, CH_2Cl_2 and water were added. The organic layer was washed with water, acetic acid (10% v/v, 3×), water, NaOH (10% w/w), and brine, and subsequently dried over $MgSO_4$. The solvent was removed under reduced pressure. In a first cleaning step, the residue was separated from inorganic impurities by column chromatography (silica gel, petroleum ether : CH_2Cl_2 = 4 : 1, R_f = 0.5). The resulting crude product was further purified by preparative recycling GPC. The solvent of the monodisperse product fraction was removed under reduced pressure. The residue was dissolved in CH_2Cl_2 and, by adding methanol, the product was precipitated from the BHT containing solution. The suspension was filtered through a PTFE membrane to yield **1d** as a slightly yellow solid (41.0 mg, 13.8 µmol, 57%). M.p. 62°C (LC), 130 °C (T_{cl}). ^{1}H NMR (500 MHz, CD_2Cl_2, 298 K): δ [ppm] = 7.96 (s, 4H), 7.92 (s, 4H), 7.64 – 7.61 (m, 2H), 7.35 – 7.31 (m, 4H), 7.04 (s, 4H), 7.03 (s, 4H), 4.11 – 4.00 (m, 16H), 2.41 – 2.36 (m, 6H), 2.04 – 1.91 (m, 8H), 1.72 – 1.13 (m, 256H), 0.93 – 0.79 (m, 48H). ^{13}C NMR (126 MHz, CD_2Cl_2): δ [ppm] = 159.12, 158.75, 138.95, 136.76, 135.26, 133.60, 132.22, 124.43, 123.37, 113.08, 111.92, 105.84, 105.66, 93.09, 86.83, 79.17, 78.14, 71.99, 71.81, 38.66, 38.33, 32.54, 32.49, 32.09, 30.72, 30.59, 30.38, 30.35, 30.30, 30.26, 30.22, 30.00, 29.93, 27.55, 27.43, 23.26, 21.48, 14.45. MALDI-MS (DCTB): *m/z* (%) = 6498.5 (<5) $[2M]^{\cdot+}$, 3750.7 (<5) $[M+2DCTB]^{\cdot+}$, 3500.3 (36) $[M+DCTB]^{\cdot+}$, 3250.0 (100) $[M]^{\cdot+}$. $C_{230}H_{356}O_8$ requires 3246.75. GPC (PS calibration): single peak at $M_{peak} = 4.45 \times 10^3$ g mol^{-1} (M_w = 4.84×10^3 g mol^{-1}).

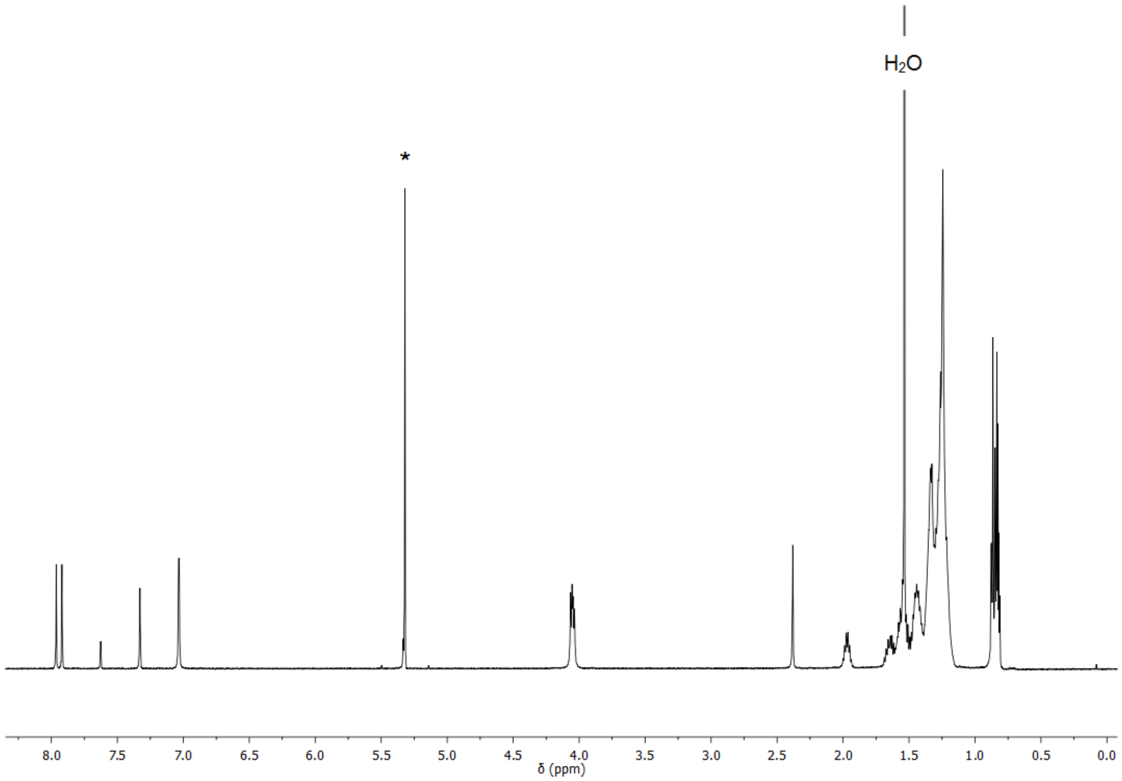

Figure S56: ^{1}H NMR (500 MHz, CD_2Cl_2) of **1d** (* = NMR solvent residual peak).

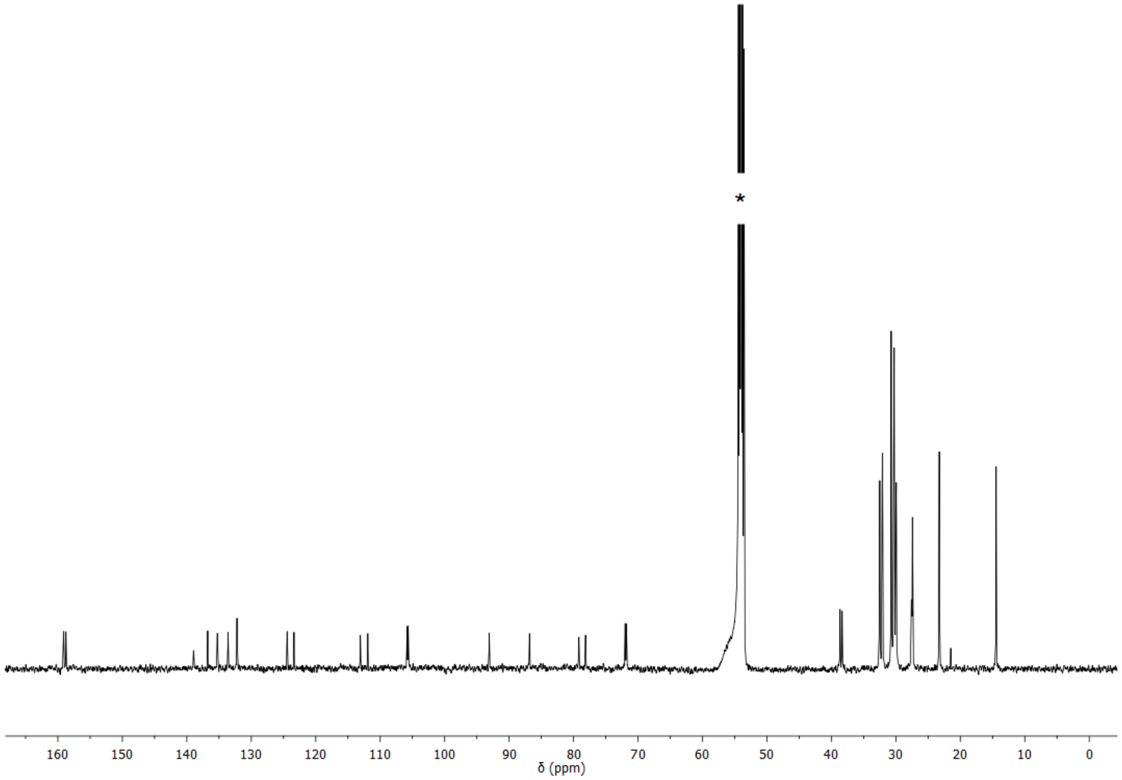

Figure S57: ^{13}C NMR (125 MHz, CD_2Cl_2) of **1d** (* = NMR solvent residual peak).

References

1. Vollmeyer, J.; Jester, S.-S.; Eberhagen, F.; Prangenberg, T.; Mader, W.; Höger, S., *Chem. Commun.* **2012,** *48*, 6547-6549.

2. Cooke, R. G.; Johnson, B. L.; Owen, W. R., *Aust. J. Chem.* **1960,** *13*, 256-260.

3. Roedig, A., Methoden der organischen Chemie. In *Houben-Weyl*, 4 ed.; Thieme: Stuttgart, 1960; Vol. 5, p 517.

4. Wheeler, H. L.; Liddle, L. M., *Amer. Chem. J.* **1909,** *42*, 441-461.

5. Potts, K. T., *Journal of the Chemical Society (Resumed)* **1953,** *42*, 3711-3712.
